# Calcification of the abdominal aorta is an under-appreciated cardiovascular disease risk factor in the general population

**DOI:** 10.3389/fcvm.2022.1003246

**Published:** 2022-10-06

**Authors:** Anurag Sethi, D. Leland Taylor, J. Graham Ruby, Jagadish Venkataraman, Elena Sorokin, Madeleine Cule, Eugene Melamud

**Affiliations:** Calico Life Sciences LLC, South San Francisco, CA, United States

**Keywords:** abdominal aorta calcification, cardiovascular disease(s), myocardial infarction, Dual-Energy X-ray Absorptiometry (DEXA), genome-wide association study (GWAS), machine learning

## Abstract

Calcification of large arteries is a high-risk factor in the development of cardiovascular diseases, however, due to the lack of routine monitoring, the pathology remains severely under-diagnosed and prevalence in the general population is not known. We have developed a set of machine learning methods to quantitate levels of abdominal aortic calcification (AAC) in the UK Biobank imaging cohort and carried out the largest to-date analysis of genetic, biochemical, and epidemiological risk factors associated with the pathology. In a genetic association study, we identified three novel loci associated with AAC (*FGF9, NAV9*, and *APOE*), and replicated a previously reported association at the *TWIST1/HDAC9* locus. We find that AAC is a highly prevalent pathology, with ~ 1 in 10 adults above the age of 40 showing significant levels of hydroxyapatite build-up (Kauppila score > 3). Presentation of AAC was strongly predictive of future cardiovascular events including stenosis of precerebral arteries (HR~1.5), myocardial infarction (HR~1.3), ischemic heart disease (HR~1.3), as well as other diseases such as chronic obstructive pulmonary disease (HR~1.3). Significantly, we find that the risk for myocardial infarction from elevated AAC (HR ~1.4) was comparable to the risk of hypercholesterolemia (HR~1.4), yet most people who develop AAC are not hypercholesterolemic. Furthermore, the overwhelming majority (98%) of individuals who develop pathology do so in the absence of known pre-existing risk conditions such as chronic kidney disease and diabetes (0.6% and 2.7% respectively). Our findings indicate that despite the high cardiovascular risk, calcification of large arteries remains a largely under-diagnosed lethal condition, and there is a clear need for increased awareness and monitoring of the pathology in the general population.

## Introduction

Vascular calcification—pathological deposition of hydroxyapatite crystals can occur throughout the vascular system, including large arteries such as the aorta, carotids, and tibial arteries, as well as in smaller vessels such as coronary arteries and skin capillaries. It commonly occurs in the elderly and in individuals with pre-existing conditions such as end-stage renal disease (ESRD) and diabetes (1). The etiology of vascular calcification is not well understood, but at least two distinct forms are known to exist—intimal layer calcification associated with atherosclerotic plaque and medial layer calcification internal to the tunica media layer of blood vessels. Intimal layer calcification is attributed to inflammatory hypercholesterolemia and calcification of plaque deposits, while medial layer calcification is attributed to the growth of hydroxyapatite crystals within the blood vessel walls as a consequence of hyperphosphatemia and/or hypercalcemia (2, 3).

Although calcification can be found in all major arterial vessels, it has been most extensively measured in high CVD risk subpopulations in the coronary arteries *via* computed tomography measurement and in the carotid artery *via* ultrasound measurement. At these sites, the severity of pathology was found to be a prognostic indicator of poor cardiovascular outcomes (4–6). However, largely due to the lack of routine monitoring, little is known about the prevalence of pathology at other sites. For example, abdominal artery calcification (AAC) is typically only detected as an incidental finding in the spine computed tomography (CT) or Dual-Energy X-ray Absorptiometry (DEXA) scans aimed at the measurement of spine bone mineral density (BMD) and fractures.

In this study, we present the largest epidemiological and genetic analysis of abdominal artery calcification (AAC) to date. Our analysis is based on imaging data from UK Biobank (UKBB). Unlike disease-enriched cohorts, the UKBB cohort is largely representative of the middle-older aged (40–70 yr) UK population, although with a bias toward healthier volunteers (7). To date, more than 40,000 DEXA scans have been collected and up to 100,000 will be collected in the next 5 years. Although these scans were not originally designed to look for pathological calcification, using machine learning (ML), we developed a fully automated methodology for detection and quantification of AAC in lateral DEXA scans. The baseline and imaging visits in UKBB are on average 8 years apart, thus the dataset provides a unique opportunity to investigate factors that contribute to the development of AAC pathology, as well as the possibility to look at outcomes that are associated with the presence of pathology.

Our analysis shows that AAC (i) is a highly prevalent pathology, (ii) occurs in the absence of traditional risk factors such as hyperphosphatemia and CKD, (iii) is a strong predictor of multiple cardiovascular and respiratory diseases, (iv) contributes to MI events independently of LDL and statin usage. In addition, we also perform a genome-wide genetic association study (GWAS) of AAC in the UKBB and meta-analysis with data from the CHARGE consortium, identifying three novel genetic risk factors linked to AAC pathology.

## Materials and methods

### Study population: DEXA imaging subcohort of UK Biobank

**Figure 1:**
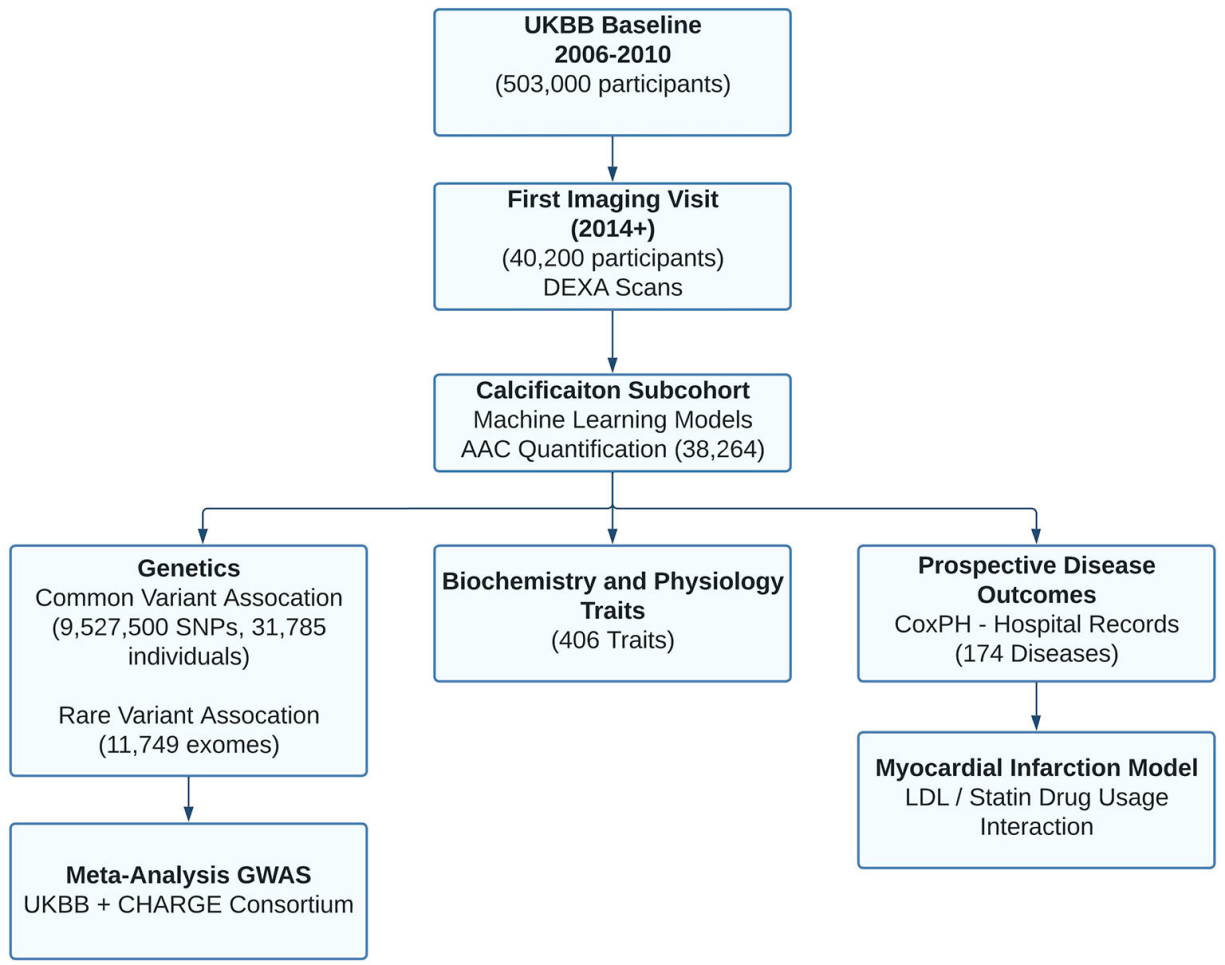
Overview of analysis and study population. This study focuses on the subset of ~40K individuals with lateral spine DEXA scans collected during the first imaging visit to UK Biobank. Machine learning algorithms enabled automatic quantification of aortic calcification across the majority of participants (~38K). We characterized genetics (GWAS, RVAS, fine mapping, colocalization, genetic correlations), epidemiological, and prospective disease outcomes post-imaging visit. Using statin prescription data and LDL measurements, we further investigate the contribution of AAC as an independent predictor of Myocardial infarction.

The UKBB imaging study aims to generate detailed images on 100,000 of the ~500,000 total UKBB participants by 2023 (8). These images span a variety of modalities including lateral spine DEXA scans. From 2014 to February 2020, 48,705 participants participated in the imaging cohort (Figure 1). The current analysis includes a subset of 38,264 participants for whom lateral spine DEXA imaging scans were available at the first imaging visit (Lunar iDXA densitometer; GE Healthcare, Chicago, Illinois)—subsequently referred to as the calcification subcohort. We characterized the entire UKBB cohort and calcification subcohort across a variety of biochemical and physiological measures ascertained at the baseline visit (2006–2010) and at the imaging visit (2014+).

We calculated the percentage of participants with comorbidities based on inpatient hospital records (ICD10 only; main or secondary diagnoses; Field 41270) at any time prior to the baseline visit, and also at any time prior to the imaging visit for the calcification subcohort. Compared to the entire UKBB cohort, the calcification subcohort exhibited slightly fewer comorbidities (Table 1). Within the calcification subcohort, comorbidities increased in the ~8 years that spanned between recruitment and imaging recall. Overall, the calcification subcohort was mostly free of inpatient diagnosis with only 2.7% of participants diagnosed with diabetes and 0.6% with CKD (Table 1).

**Table 1:** Baseline characteristics of the UK Biobank (UKBB) cohort and the calcification subcohort.

	**UKBB cohort baseline visit (2008–2010)**	**Imaging subcohort baseline visit (2008–2010)**	**Imaging subcohort first imaging visit (2014+)**
n	502,604	38,264	38,264
Age (years)	56.3 (8.1)	54.9 (7.5)	63.6 (7.6)
% Females	54.40%	48.26%	48.26%
BMI	27.43 (4.80)	26.63 (4.23)	26.56 (4.41)
SBP	139 (20)	137 (19)	138 (18.5)
Pulse	68 (11.7)	68.0 (11.0)	69.0 (12.1)
Smoker (current)	10.50%	6.50%	3.55%
Smoker (previous)	34.50%	32.50%	33.02%
**Comorbidities**			
% Hypertension	7.88%	4.13%	13.23%
% T1D	0.42%	0.15%	0.26%
% T2D	1.83%	0.71%	2.46%
% MI	2.12%	1.12%	3.85%
% Stroke	0.14%	0.07%	0.10%
% CAD	0.99%	0.57%	1.36%
% CKD	0.13%	0.02%	0.61%
**Biomarkers**			
Glucose (mmol/L)	5.12 (1.24)	5.00 (0.98)	
HbA1c (mmol/mol)	35.2 (6.78)	35.0 (5.07)	
Trig (mmol/L)	1.75 (1.45)	1.67 (0.98)	
LDL (mmol/L)	3.56 (0.87)	3.58 (0.83)	
HDL (mmol/L)	1.45 (0.38)	1.47 (0.37)	
Cholesterol (mmol/L)	5.69 (1.14)	5.72 (1.09)	
Serum Phosphate (mmol/L)	1.16 (0.16)	1.15 (0.16)	
Serum Calcium (mmol/L)	2.38 (0.09)	2.37 (0.09)	
Serum Creatinine (umol/L)	72.31 (18.55)	72.63(14.16)	
Cystatin C (mg/L)	0.91 (0.18)	0.87 (0.13)	

The following second level ICD10 codes from the electronic health records of all participants were used to identify the number of participants with different comorbidities prior to the appropriate visit to the center: I10 - Hypertension, E10 - Type 1 Diabetes (T1D), E11 - Type 2 Diabetes (T2D), I25 - Myocardial Infarction (MI), I64 - Stroke, I21 - Coronary Artery Disease (CAD), and N18 - Chronic Kidney Disease (CKD).

BMI, body mass index; SBP, systolic blood pressure; HbA1c, glycated hemoglobin; Trig, triglycerides; LDL, low density lipoprotein; HDL, high density lipoprotein.

The decline in number of current smokers at the second visit (2014+) is likely due to adaptation of a healthier lifestyle by the participants.

### Manual annotation of abdominal aortic calcification in DEXA

**Figure 2:**
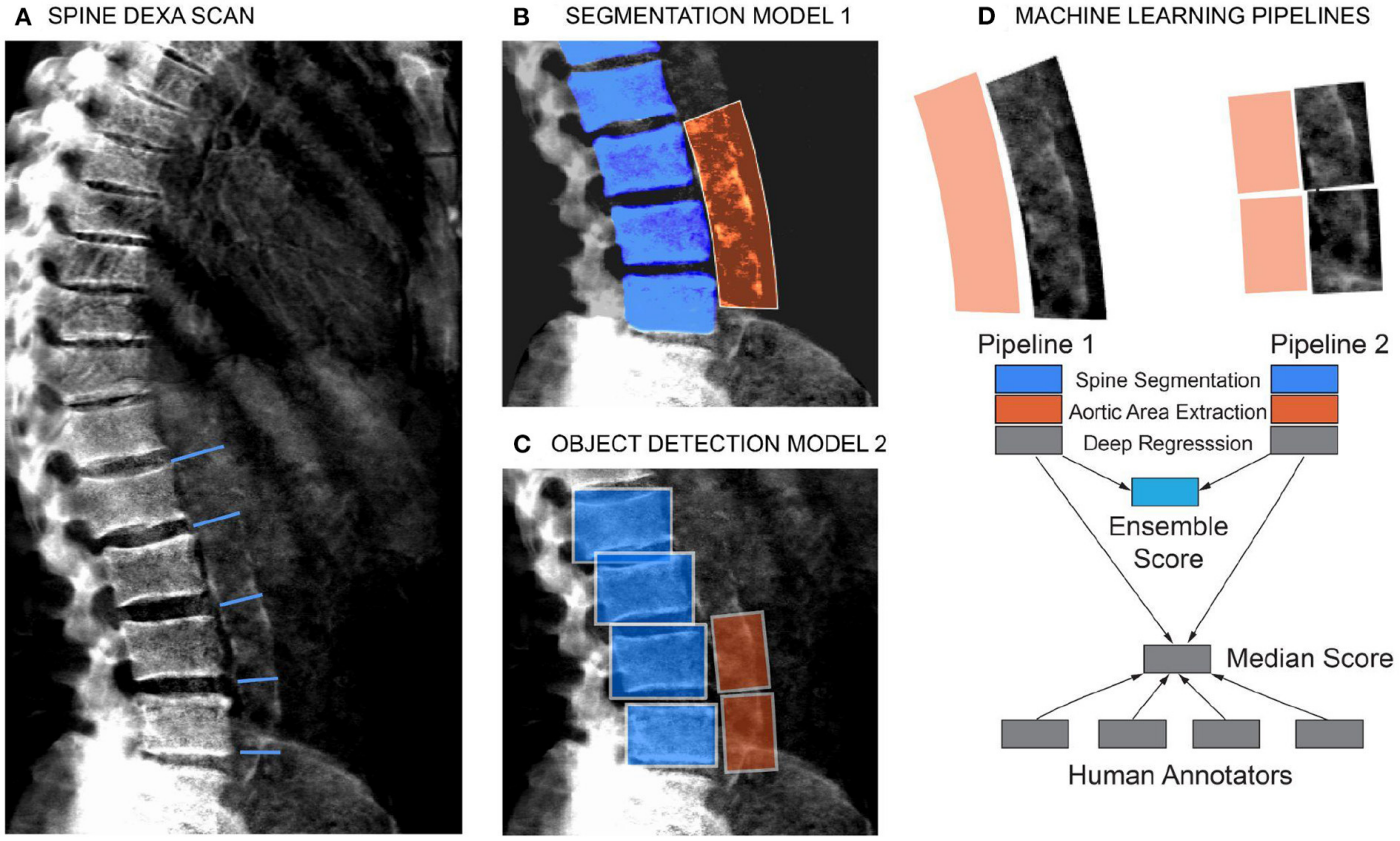
Schematic of the machine learning pipeline to quantify AAC. Overview of the two independent machine learning pipelines used to quantify AAC. Lumbar vertebra detection (blue). And extracted aortic regions are highlighted in orange. An ensemble AAC score, combining predictions from both pipelines, performed the best on the validation dataset. **(A)** Representative DEXA scan (manual annotation score = 15). **(B)** Pipeline 1: Segmentation of the lower spine using U-Net architecture (blue). **(C)** Pipeline 2: Object detection–box segmentation of individual lumbar vertebrae (blue). **(D)** AAC scores are quantified using a neural network (NN) based regression against human-derived aortic calcification scores.

To quantitate the extent of calcification in the abdominal aorta we adopted a scoring system developed by Kauppila and coworkers (9). Briefly, the calcification score is computed in the lumbar spine area (L1 to L4 vertebra), by manually scoring the visible amount of calcification in the anterior and posterior wall of the abdominal aorta (Figure 2A). The calcification score of each vertebral section ranges from 0 to 3. Score 0 represents without any calcification; score 1 represents calcification length <1/3 of the vertebra; score 2 represents the calcification length spanned from 1/3 to 2/3 of the vertebra; score 3 represents calcification length greater than 2/3 of the vertebra. The scores from L1 to L4 are summed to calculate the total AAC score with a maximum of 24. In practice, the level of calcification in the L1-L2 region can be hard to ascertain/distinguish from the background, and scores are driven by calcification in the L3-L4 region.

Four annotators independently scored 1,300 random images using the 24-point scheme as described above. Quantitation of AAC in noisy DEXA imaging is a challenging task and there was significant variability between annotators (mean absolute error ranging from 0.66 to 1.49, Supplementary Figure S1). However, intra-annotator variability did not have a large effect on the median annotation scores (Pearsons' correlation 0.93, Supplementary Figure S2). Subsequently, the median annotation values for 1,000 images were used for training and testing machine learning models, and the remaining 300 images were used for validation (see Supplementary material).

### Scoring AAC using machine learning models

For the ML models, we first considered a single-step regression model to directly predict AAC scores from the images; however, such an approach performed poorly due to substantial background noise within the DEXA images (Supplementary Figure S3). To address the noisy image issue, we developed a step-wise approach to score aortic calcification levels only in the lumbar spine region. The steps in the pipeline were as follows:

1. Segmentation of the lower spine region—pelvis and lumbar vertebrae.
2. Localization of the aortic region using a spine-curve fitting method.
3. Regression on the localized region to predict the calcification levels from the aortic region.

**Figure 3:**
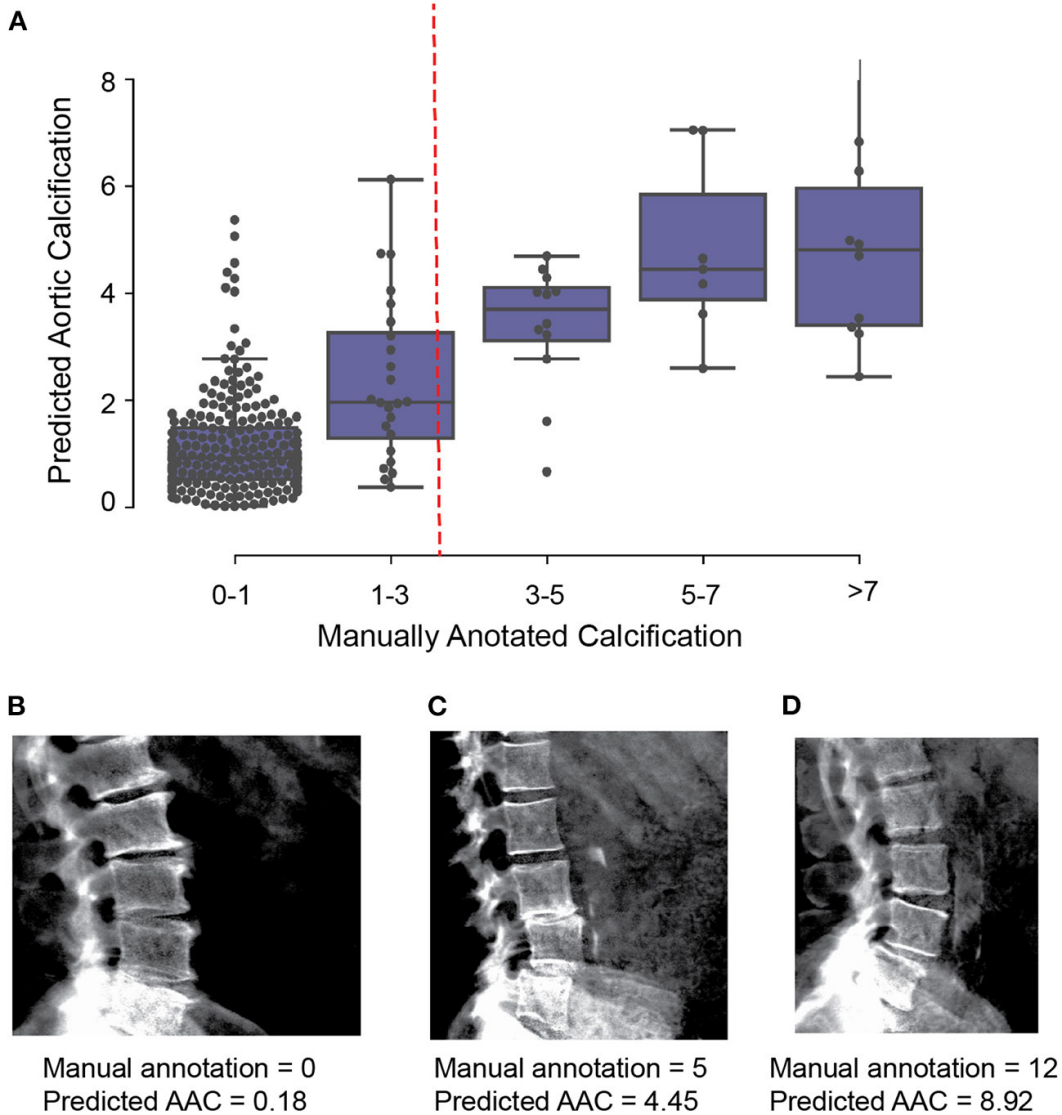
Comparison of machine learning based AAC scores to manually annotated AAC scores. **(A)** Correlation of manually annotated calcification scores with ensemble machine learning (ML) scores on the 300 images in the validation set. **(B–D)** Comparison of ML and manual scores for participants with low, medium and high levels of calcification within the test dataset.

We evaluated two independent machine learning approaches for each step, the first was based on an image segmentation model and the second was based on an object detection model (Figure 2). The overall performance of each pipeline was dependent on the accuracy of each step in the pipeline (Supplementary Figures S4–S9), but both approaches achieved similar accuracy on the test dataset (Pearson's correlation ~ 0.6). The ensemble score, generated by averaging the score of each pipeline, exhibited the best performance on the test dataset (Pearson's correlation ~ 0.67 and mean absolute error ~1.27; Figure 3, Supplementary Table S3). We therefore used the ensemble method to predict AAC scores in the whole imaging dataset.

Full details of the machine learning methodology can be found in the supplement. Open source code is freely available in the GitHub repository https://github.com/calico/AAC_scoring.

## Results

### Calcification of abdominal aorta is a highly prevalent pathology

**Figure 4:**
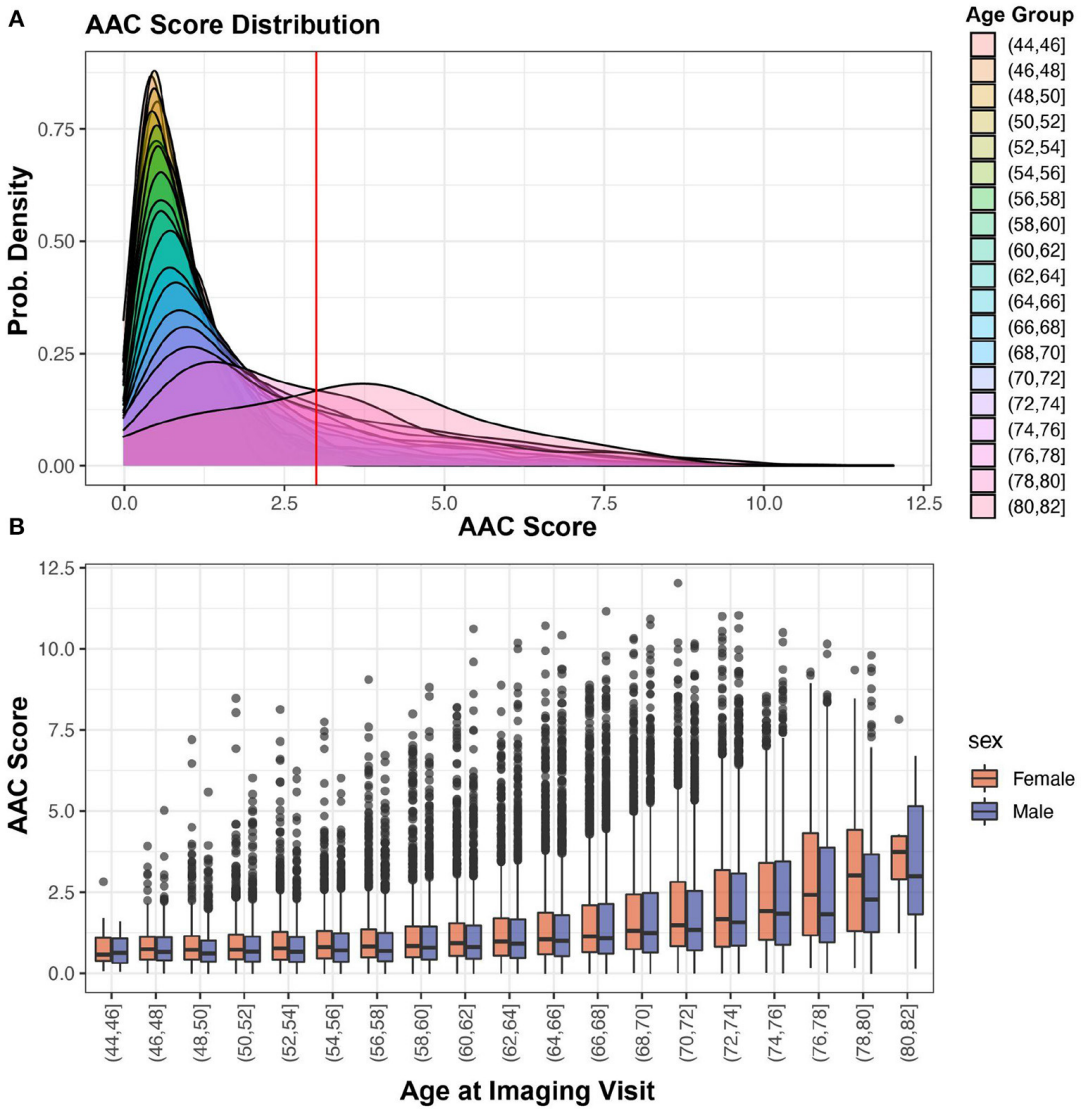
AAC Prevalence. **(A)** Distribution of predicted AAC scores across 38,264 participants. The red line shows calcification score at mean plus one standard deviation (score ~ 2.93). **(B)** Relationship between predicted AAC scores and age of the participants. There is a clear age-dependent increase in calcification in each age strata, with the median value, variance, and the number of extreme cases increasing across both sexes.

The distribution of the calcification score across 38,264 participants is shown in Figure 4A—the distribution is skewed toward low values (mean ~1.47, std.dev ~1.49), with the majority of participants (~88%) having no detectable or mild calcification (score <3). We find that ~11.6% of the population falls into a long tail above 1 standard deviation (score > 3). To make sure that ML derived prevalence distribution was not biased, we compared it to the distribution of manually annotated calcification levels from 1,300 randomly chosen scans. Similar to ML, in manually annotated scans a similar fraction (~10.4%) had moderate to high calcification (AAC score > 3).

Both the severity of calcification and the number of extreme cases increase sharply as a function of age. The trend is similar for both men and women across age groups (Figure 4B). While it is known that the prevalence of medial layer calcification in patients with end-stage renal disease (ESRD) and diabetes can be as high as 41% (2), we were surprised to observe such high prevalence in the general population given that only 3.3% of the UKBB cohort has kidney disease and diabetes at baseline.

### AAC is a prognostic risk factor of future cardiovascular events

We further investigated the effect of AAC as a prognostic factor for the development of a broad spectrum of diseases in EHR records. In the UKBB, EHR records are encoded using ICD10 diagnostic codes and extend to approximately 2.9 years post imaging visit (median follow-up time). To get a more functional categorization of diseases we converted ICD10 to Phecode disease codes (10). We estimated the hazard of AAC using a Cox proportional hazard (CoxPH) model (11) using multivariate models. In total 174 disease classes were evaluated (Supplementary Table S14).

**Figure 5:**
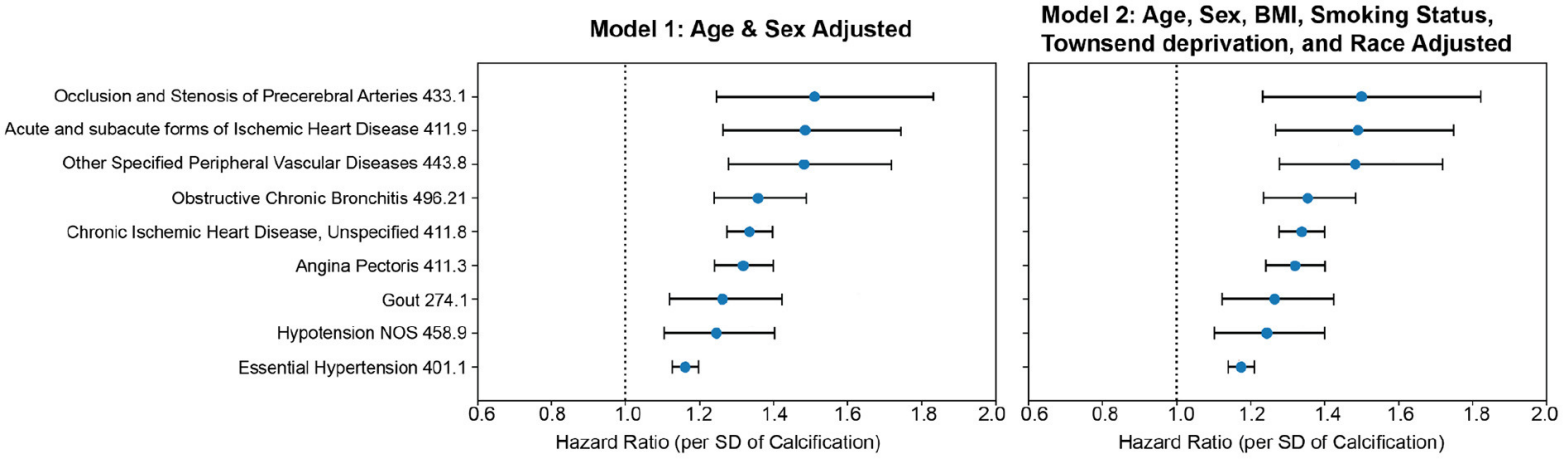
CoxPH association of AAC with prognosis outcomes. Forrest plot shows hazard ratios for statistically significant disease associations after multiple hypothesis testing. The blue dots represent the mean hazard ratio, the error bars represent 95% confidence intervals. We tested 174 diseases with more than 25 events post-baseline for association with AAC using the Cox PH model. Only 10 associations that passed multiple hypothesis tests in model 1 were reported here. The number of events post-baseline for all tested diseases can be found in Table S14. (Methods Section “Prognostic analyses of aortic calcification”).

**Figure 6:**
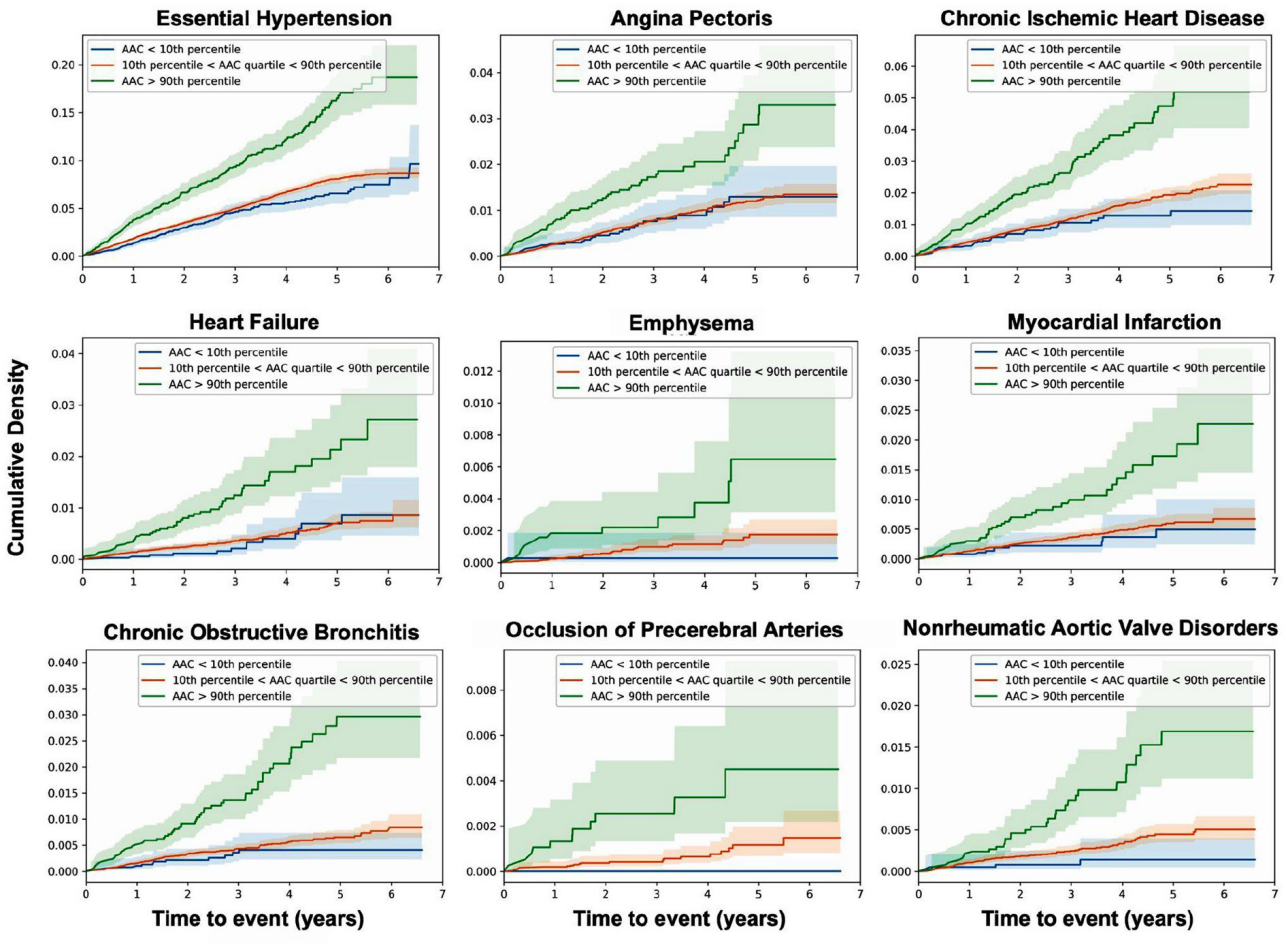
AAC is a prognostic risk factor for a large class of cardiovascular and respiratory outcomes. Cumulative density plots for statistically significant (*p*_Bf_ < 0.05) AAC disease associations—post imaging visit follow-up time. Population stratified into risk groups based on AAC levels—green (highest 10%, AAC score > 3), red (lowest 10%, AAC score ~0), blue (10–90% AAC). The shaded regions represent the 95% confidence interval for each subset of participants.

We calculated time-to-event as the time between the time of imaging visit and the first occurrence of disease (for participants without prior history of disease), thus allowing us to establish a predictive value of AAC for time to disease occurrence. We considered two models: (i) adjusted for age and sex (Model 1) and (ii) adjusted for age, sex, BMI, socioeconomic status, race, and smoking (Model 2). Among environmental factors, both former and current smoking was the strongest predictor of AAC with an effect size comparable to age (beta ~ 0.12, Supplementary Figure S14). As smoking is also a well-known risk factor in multiple diseases, it was important that we include this adjustment in assessing the independence of AAC signal in disease outcomes. All p-values were calculated using the two-tailed t-statistic and corrected for multiple testing using Bonferroni correction. Significantly associated diseases (*p*_Bf_ < 0.05) are shown in Figure 5, and representative survival curves in Figure 6.

We find that calcification is a significant risk factor for 10 disease conditions, including multiple cardiovascular diseases such as occlusion of precerebral arteries (HR~1.5), nonrheumatic aortic valve disorders (HR~1.35), myocardial infarction (HR~1.36), chronic ischemic heart disease (HR~1.3), heart failure (HR~1.25), angina pectoris (HR~1.2), hypertension (HR~1.15), emphysema (HR~1.45), COPD (HR~1.3), and emphysema (HR~1.5). While some conditions such as stroke, aortic valve disorders, and MI have been linked to AAC before (12–16), to our knowledge this is the first time AAC has been implicated as a prognostic predictor of respiratory disorders. The mechanism by which lung associations are arising is not clear, while it is possible that calcification of arteries might be affecting lung function, it is also possible that a shared environmental risk factor such as smoking is independently contributing to both development of AAC, CVD, and lung function decline.

### Physiological and biochemical predictors of AAC levels

**Figure 7:**
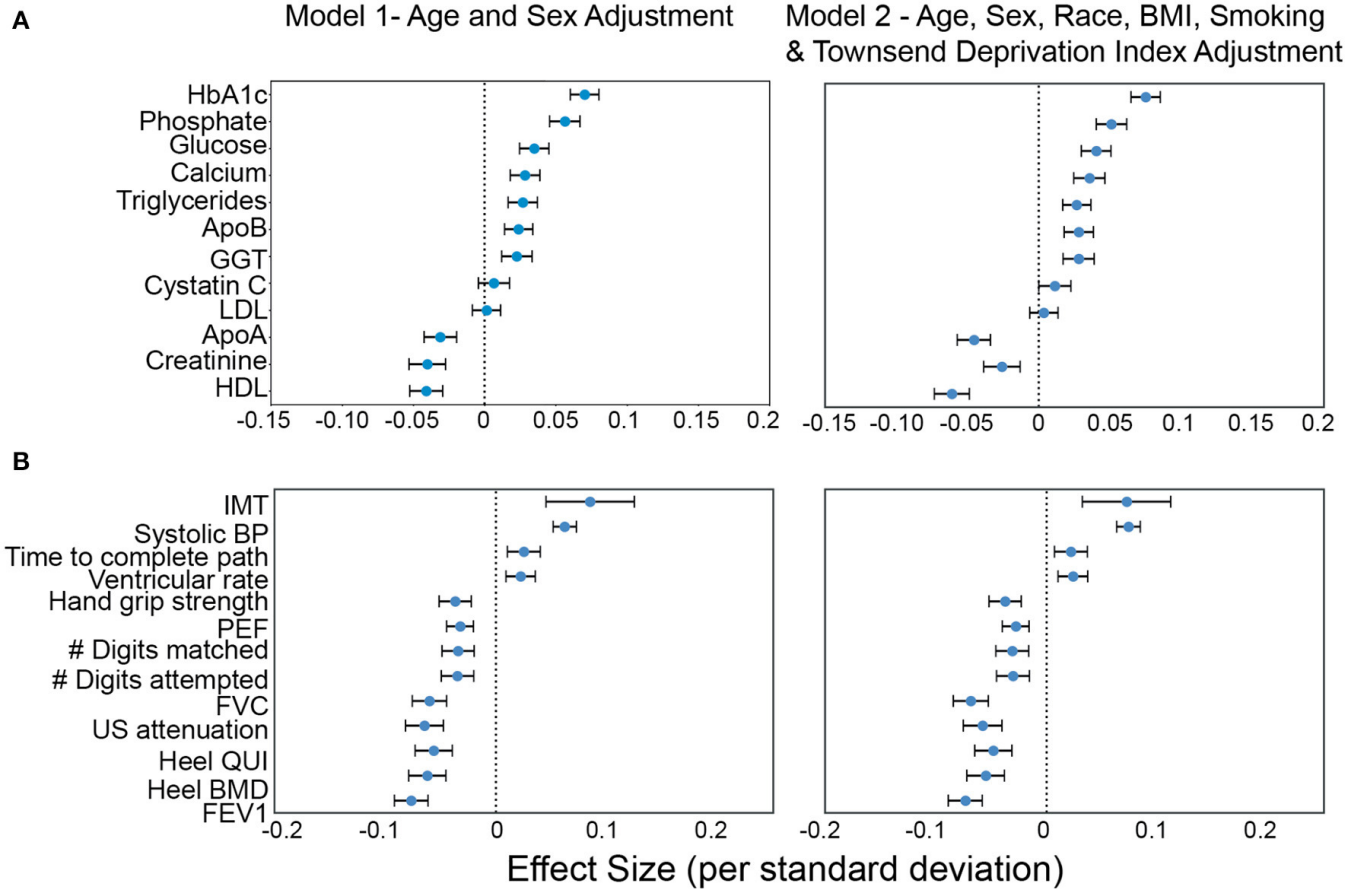
Association of AAC with **(A)** molecular biomarkers, and **(B)** physiological parameters. Briefly, AAC is modeled as a linear function of each covariate adjusted for age + sex (model1) and age+sex+confounders (model2). The blue dot represents the mean effect size per standard deviation of different covariates while error bars represent the 95% confidence interval. With the exception of LDL and cystatin C, only covariates that passed multiple hypothesis testing in model 1 are shown (Bonferroni corrected *p* <1.2e-4; see Methods Section Results). We include LDL and cystatin C as they are discussed in the text. Age during imaging visits was used to adjust for calcification levels. All other covariates and measurements were taken during the baseline visit.

To get further insight into physiology that might be contributing to the development of AAC, we evaluate all blood biochemistry, CBC, and physiological measures collected at the UKBB baseline for an association with AAC at the imaging visit (~ 8 year follow up). All statistically significant associations (*p*_Bf_ < 0.05), adjusted for age and sex, are shown in Figure 7 and Supplementary Figures S6, S12.

Increased intima medial thickness (IMT) of the carotid artery was the strongest risk factor linked to AAC. It is possible that increases in IMT is due to a combination of plaque buildup and/or buildup of calcium deposits within this artery, but at this point, it is hard to know which component is the causal factor. We also observed a strong association between increased Systolic BP and AAC. The combination of higher IMT and Systolic BP provides a plausible mechanism by which AAC might be contributing to increasing cardiovascular events.

**Figure 8:**
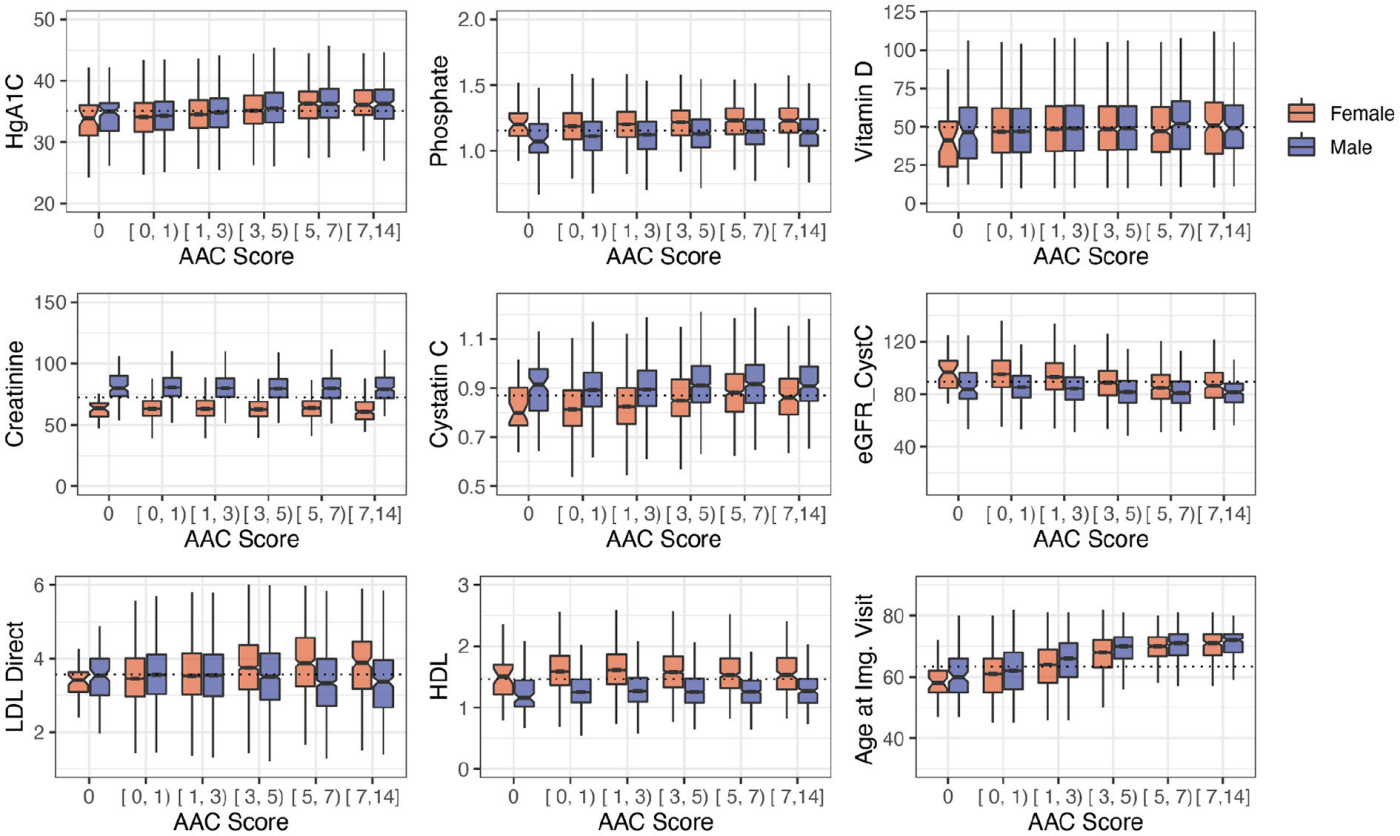
Distribution plots for a selected set of biomarkers plotted as a function of calcification severity. Elevated HgA1c and phosphate were the strongest biochemical predictors of AAC identified in adjusted regression models. LDL and kidney biomarkers (Vitamin D, Creatinine, Cystatin C, eGFR are not statistically significantly associated with AAC after age adjustment (see Figure 7).

Among biochemical factors, increased levels of Hemoglobin A1C, glucose, phosphate, and triglycerides were the strongest predictors of AAC (Figure 7, Supplementary Figures S12, S13). While we cannot rule out reverse causation, this finding suggests that diabetes is a significant contributor to the development of AAC (Supplementary Table S5). In contrast to other studies that looked at calcification in the CKD context, we find that the majority of participants (~96.8%) with significant AAC do not have elevated levels of creatinine and cystatin C levels, and are not hyperphosphatemic (serum phosphate > 1.46mM/liter, Figure 8, Supplementary Figure S16). In fact, we observe that AAC is anticorrelated with creatinine levels (*p*_Bf_ = 3.03e−7).

To further elucidate the relationship between kidney function and AAC, we estimated glomerular filtration rate (eGFR) from the cystatin C levels for all participants (*p*_Bf_ = 1) (Supplementary material). Across the first four quintiles of AAC, eGFR estimates were within normal kidney function ranges (eGFR ~90, Supplementary Figure S17). The participants in the highest quintile of AAC showed slightly reduced kidney function (eGFR ~80). Given the low prevalence of CKD in this cohort (< 0.5%), this observation suggests that the majority of people who develop calcification do so in the background of normal functional kidneys.

To further confirm the validity of these findings, we carried out a replication study of biochemical associations in the Osteoporotic Fractures in Men (MrOS) cohort (17). The MrOS study is a multi-center prospective, longitudinal, observational study designed to examine risk factors associated with longer term health outcomes in eldery men. In MrOS, AAC was evaluated for the risk of a hip fracture among 5,400 men with baseline lateral DXA scans using the same Kauppila score measure as in this manuscript (17). All major UKBB findings were confirmed in the MrOS cohort, including: (i) increased levels of AAC with phosphate, (ii) increased levels of AAC with HbA1C, glucose, triglycerides, (iii) increased levels of AAC with systolic blood pressure, and (iv) anticorrelation with creatinine and HDL (Supplementary Figure S13).

The overall picture that emerged from this analysis is that aging of arteries, diabetes/pre-diabetes, and elevated serum phosphate are the greatest risk factors for the development of AAC.

### AAC is an LDL-independent risk factor for myocardial infarctions

Another possibility is that calcification might be occurring secondary to hypercholesterolemia. In the analysis of biochemical factors, we observed that AAC is anticorrelated with HDL (*p*_Bf_=2.46e−9) and is not associated with LDL (*p*_Bf_ = 1). The prevalence of diagnosed hypercholesterolemia in the cohort was <6.5%, strongly suggesting that calcification can occur independently of LDL. However, it is also possible that lack of association might be due to the use of statins—which lowers observed LDL levels in high-risk CVD population.

**Figure 9:**
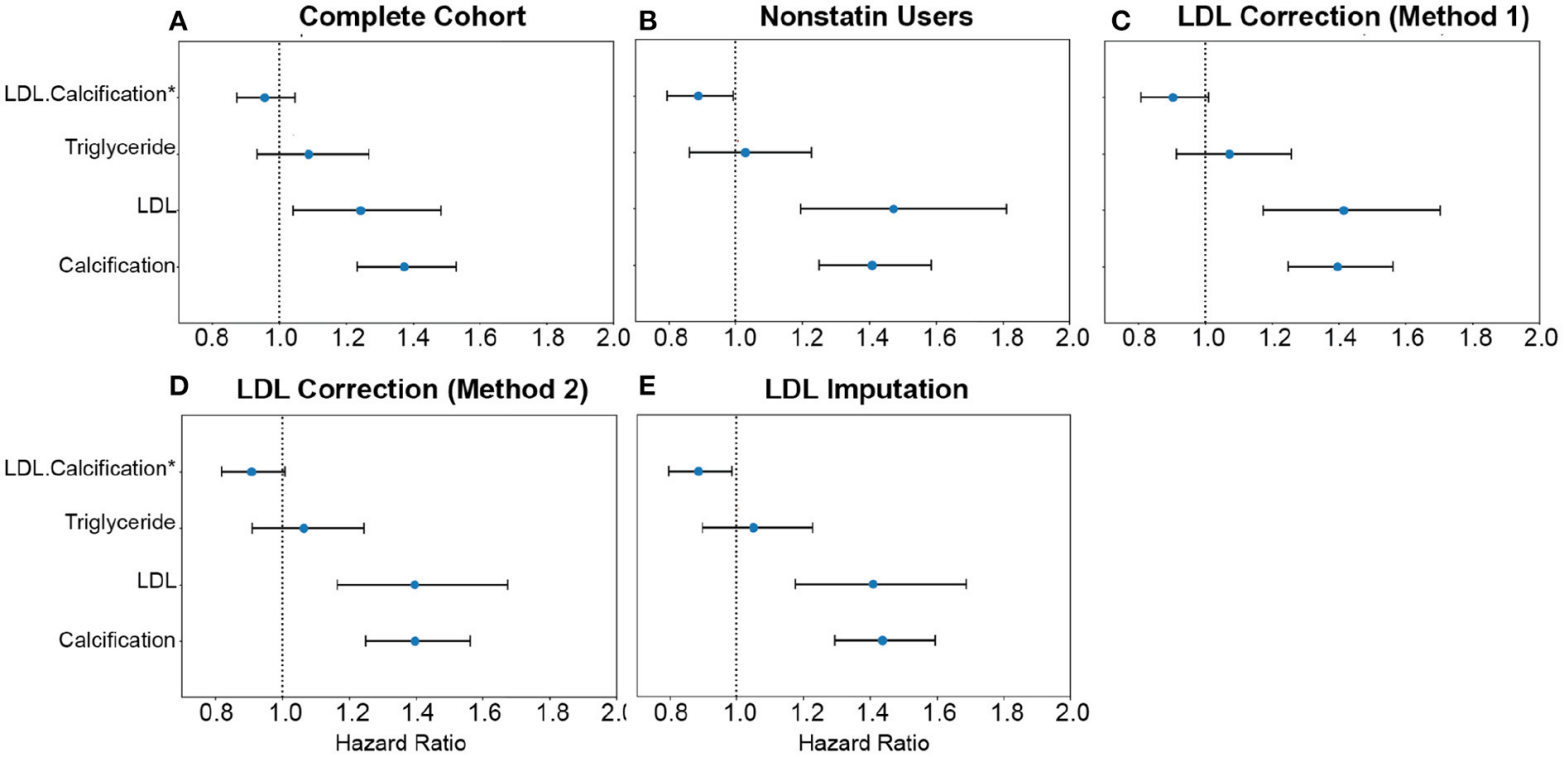
Comparison of AAC and statin-adjusted LDL hazards on Myocardial Infarction Outcome. We compared the hazard ratios for AAC, LDL, and triglycerides using a multivariate CoxPH model with age and sex covariates. **(A)** Complete cohort- a whole cohort with no statin adjustment. **(B)** Non-statin users only. **(C)** The whole cohort after adjusting for statin using method 1—assumes that LDL levels are reduced by 1.25 mmol/L in statin users. **(D)** The whole cohort method 2—assumes that LDL levels are reduced by 35% in statin users. **(E)** The whole cohort imputation of LDL levels is based on remaining biomarkers and physiological measurements. The blue dots represent the mean hazard ratio, while error bars represent 95% confidence intervals. The interaction term between LDL and calcification is starred. This analysis was done with 11.8 years of median follow-up time post-baseline visit during which LDL was measured.

To investigate this possibility, we developed a number of multivariate survival models of myocardial infarction (MI) with and without statin adjustments, and jointly model MI risk with adjusted LDL levels and AAC. Since LDL was measured at the baseline visit and AAC was assessed at the imaging visit, we repeated the analysis starting from both the baseline visit (Figure 9) as well as the imaging visit, and observed similar results (Supplementary Figure S26).

Approximately 15% of the UKBB cohort is taking statins. To obtain an unbiased estimate of LDL effect on MI risk, we estimated LDL risk after adjusting for statin usage using three different models (Supplementary material, Supplementary Figure S23). In model #1, we adjusted the LDL and triglycerides levels for statin users by increasing measured LDL and triglycerides by 1.25 mmol/L (18) (Figure 9C). In model 2, similar to the model #1, LDL and triglycerides were increased by a relative increase of 54% in statin users (18) (Figure 9D). In model #3, we imputed the untreated LDL and triglyceride levels for statin users based on blood pressure, pulse, age, sex, and non-lipid molecular biomarkers (Figure 9E). For non-statin users, LDL and triglyceride levels were maintained as measured at the baseline (Figure 9B).

**Figure 10:**
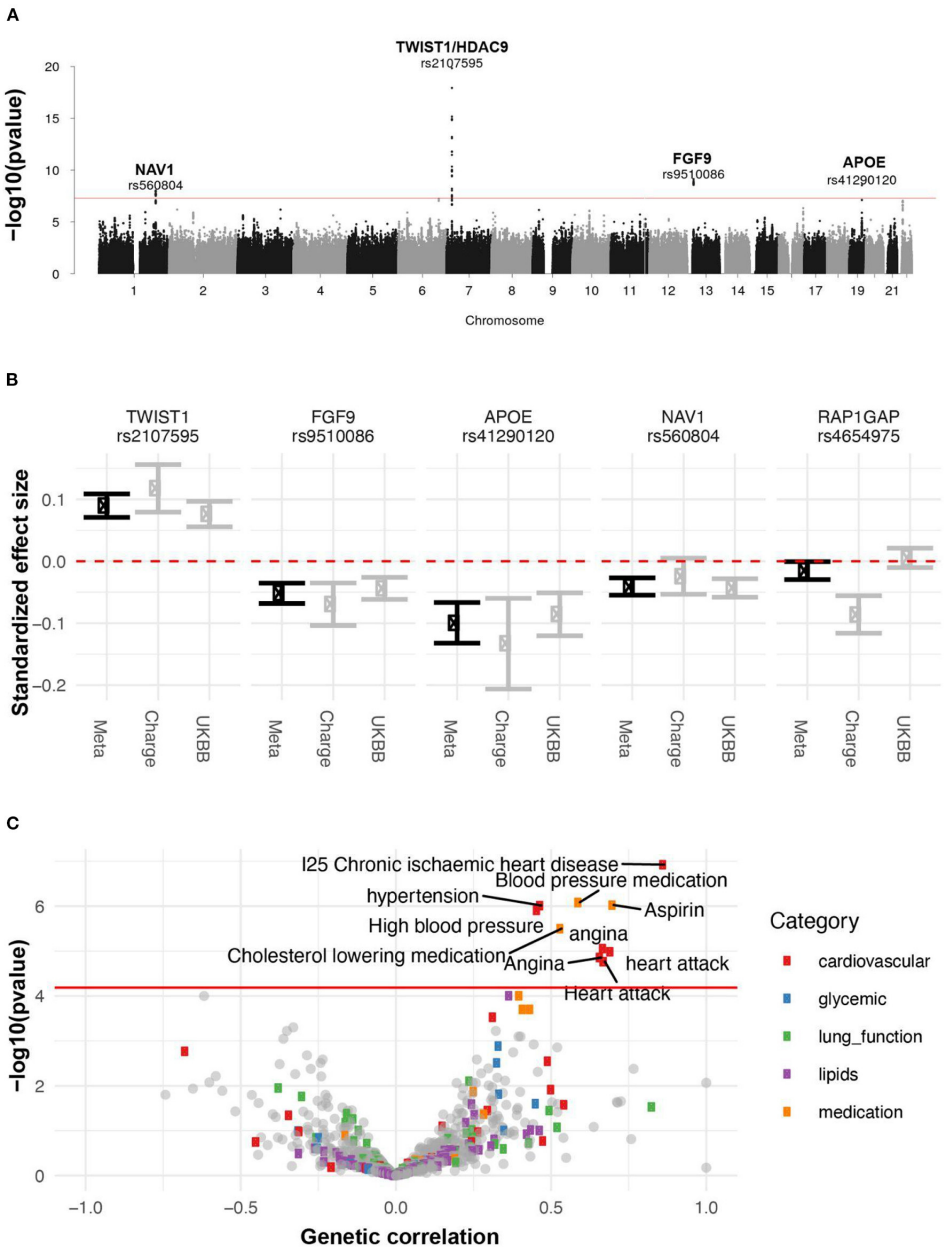
Genetic analysis of AAC. **(A)** GWAS Manhattan plot. Association of each SNP -log(p-values) ordered by chromosome. Genome-wide significance cut-off (p-value 5 x 10^−8^) is shown as a red line. **(B)** Approximate standardized effect size estimates for the UK Biobank data, CHARGE consortium data, and the meta-analysis at the four genome-wide significant loci in this study, and one additional locus previously reported by CHARGE. **(C)** Genetic correlation and p-value for correlation between AAC and other complex traits. X-axis: Estimated correlation. Y-axis: -log10(p-value). The horizontal line is at the Bonferroni-corrected *p*-value threshold.

In all statin-adjusted models, MI risk associated with severity of AAC (HR~1.4 per standard deviation) was comparable to LDL risk (HR~1.4 per standard deviation). We did not find evidence for an interaction between LDL and AAC (*p* > 0.05) in any of the models, suggesting that AAC is a hypercholesterolemia-independent risk factor for MI. These results are also consistent with our genetic analysis which did not find a genetic correlation between AAC and lipid biomarkers (Figure 10C; Supplementary Table S10). In all statin-adjusted models, we found that the adjusted LDL and triglyceride levels deconfounded the effect of statins on MI (LDL HR ~1.4 without statin adjustment, and LDL HR ~1.2 with statin usage).

We also assessed the risk of AAC for MI events in a subgroup of participants who do not take statins. Nonusers are expected to represent a healthier CVD subpopulation. In this group, AAC is still a significant risk factor for MI (HR~1.4), a value that is comparable to LDL (HR~1.4), with no significant interaction detected between LDL and AAC (*p* > 0.05). Overall, all models of AAC-LDL interaction constantly showed calcification of the abdominal artery as an LDL-independent risk factor for acute MI outcomes, indicating that risk associated with calcification cannot be addressed by the use of statins.

### Identification of AAC genetic risk factors

To identify genetic factors that may contribute to AAC, we performed rare and common variant genome-wide association studies. For the common variant association study (Figure 10, Supplementary Figure S24), we tested 9,572,557 single-nucleotide variants or polymorphisms (SNPs) with minor allele frequency at least 1% across 31,786 participants (Methods). We estimated the SNP-based heritability to be 12.4% (s.e. 1.7%; Methods). To increase our power to identify genetic effects we combined these summary statistics with those from a previous AAC study (19), conducted by the Cohorts for Heart and Aging Research in Genome Epidemiology (CHARGE) consortium (20), and performed a meta-analysis spanning 6,360,639 SNPs and 41,203 participants (Supplementary Table S9).

We identified four loci associated with AAC (*p* <5x10-8; Figure 10A). We did not detect any rare variant associations. To gain insight into a potential biological mechanism(s), we carried out three types of genetic analysis. First, we fine-mapped independent signals and potential causal variants within each associated locus. Second, we identified other traits that likely share the same underlying causal variant, performing a colocalization analysis with other UKBB phenotypes. Third, we looked for colocalizing expression quantitative trait loci (eQTLs) across 48 tissues to identify candidate effector genes. The full list of methods can be found in the supplement.

A set of consistent associations between UKBB and CHARGE consortium are summarized in Table 2 and the Supplementary Functional Analysis of GWAS Associations section. We successfully replicated a strong association at the *TWIST1/HDAC9* locus (lead SNP, rs2107595 p = 1.47x10^−20^). *TWIST1* is a regulator *RUNX2*, a key transcription factor responsible for the differentiation of osteoblasts. This locus has been linked to several cardiovascular traits including ischaemic stroke (21, 22) in large and small vessels (23, 24), coronary artery disease (CAD) (23), peripheral artery disease (25, 26), blood pressure (25, 27–29), pulse pressure (30), and MoyaMoya disease (31). This signal colocalizes with the expression of *TWIST1* in the aortic artery, but not the expression of *HDAC9* in any of the tissues considered. Based on tissue colocalization and expression analysis, it is likely that the *TWIST1* gene is responsible for the association, but the role of *HDAC9* cannot be ruled out (Supplementary Figures S19–S21).

**Table 2:** Summary of genome-wide significant associations identified in a metaanalysis of 31,786 UK Biobank participants and 9,417 CHARGE participants.

**Lead SNP**	**Locus**	***p*-value**	**Tissue and diseases colocalization**	**Description**
rs2107595	HDAC9/ TWIST1	1.47x10^−20^	Aorta Diseases: CAD, Systolic BP, Pulse Pressure	Regulation of osteogenic master regulator RUNX2. Previously implicated in AAC and in a number of CVD traits.
rs9510086	FGF9	7.38e^−10^	Tibial Artery	Role in AAC is unknown. FGF9 expression has been shown to be increased in aneurysms compared to control tissue (43).
rs560804	NAV1	9.30x10^−9^	Heart—left ventricle and atrial appendage; Diseases: Diastolic BP	Role in AAC is unknown. Recent study (33) identified NAV1 as a candidate gene for aortic valve stenosis.
rs41290120	APOE	2.9x10^−9^	CAD	Role in AAC is unknown. The lead SNP at this locus is in LD (r^2^>0.99) with the APOE e2 allele, rs7412(44).

In addition to the *TWIST1/HDAC9* locus, we identified three novel loci (*FGF9, NAV1*, and *APOE*) associated with AAC, although it remains unclear mechanistically how they may be involved. We found signals for genetic colocalization of *FGF9* and *NAV1* with cardiovascular tissue. *FGF9* and *NAV1* have been implicated in the abdominal aortic aneurysm (32) and aortic valve stenosis (33) respectively, which may indicate the observed calcification is systemic and not isolated to the abdominal aorta.

To understand which tissues/cell types might be involved in the development of AAC, we partitioned the overall SNP-based genetic heritability across the genome using different tissue and cell type annotations derived using chromatin marks and marker genes (Supplementary material). After multiple hypothesis correction, we found enrichment of AAC heritability in blood vessels, vascular endothelial cells, arteries, fat-related tissues, as well as a number of other tissues (FDR <5%; Supplementary Figure S19).

Finally, we calculated the genetic correlation between AAC and 773 other traits to understand the extent to which genetic factors are shared between AAC and other phenotypes (Methods). After adjustment for multiple testing (*p* < 6 x 10^−5^), we found that AAC is genetically correlated with cardiovascular-related diseases (hypertension, angina, heart attack, and ischaemic heart disease) and medications (cholesterol-lowering medication, aspirin, and blood pressure medication) (Figure 10C, Supplementary Table S10). These results are largely consistent with the correlations we observe with physiological traits. For instance, we do not observe a phenotypic association between hyperlipidemia and AAC, nor do we observe a genetic correlation between AAC and lipid levels.

In summary, our genetic analysis implicates three novel genes (*FGF9, NAV1*, and *APOE*) in the pathogenesis of AAC and confirms one known association (*TWIST1/HDAC9*). Both heritability enrichment and expression colocalization analyses point to a key role for vascular tissue in the pathogenesis of vascular calcification.

## Discussion

In this study, using image-based machine learning methodology, we quantified AAC from 38,264 participants from a UK Biobank, the largest scale analysis of pathology to date. While the prevalence and heritability of AAC has been previously investigated in smaller longitudinal cohorts (34, 35), the pathology has never been studied at scale in healthy individuals. We found that calcification of the abdominal aorta is a common pathology with a relatively high prevalence in the general population (>10% of participants). Both men and women were affected to the same extent and with similar aging trajectories.

Consistent with previous reports on AAC, we found that the presentation of AAC is strongly predictive of poor outcomes across disease space—linked to increased incidence of multiple diseases, including future myocardial infarctions, chronic ischemic heart disease, and heart failures (36). Intriguingly, we also observed an association of AAC with poor respiratory outcomes such as COPD and emphysema, suggesting that consequences of AAC extend beyond the cardiovascular space. It is unclear how such a broad set of negative outcomes can be explained. It is possible that calcification of large arteries causes decreased vessel plasticity, resulting in increased systolic blood pressure, and broad stress across the entire cardiovascular system. It is also possible that AAC is a readout of calcification in other smaller blood vessels, and as such, it is not a direct contribution to CVD, but rather an indicator of calcification in other sites. Unfortunately, due to the high noise and low resolution of the DEXA scans, detection of calcification in the smaller vesicles cannot be easily achieved.

**Figure 11:**
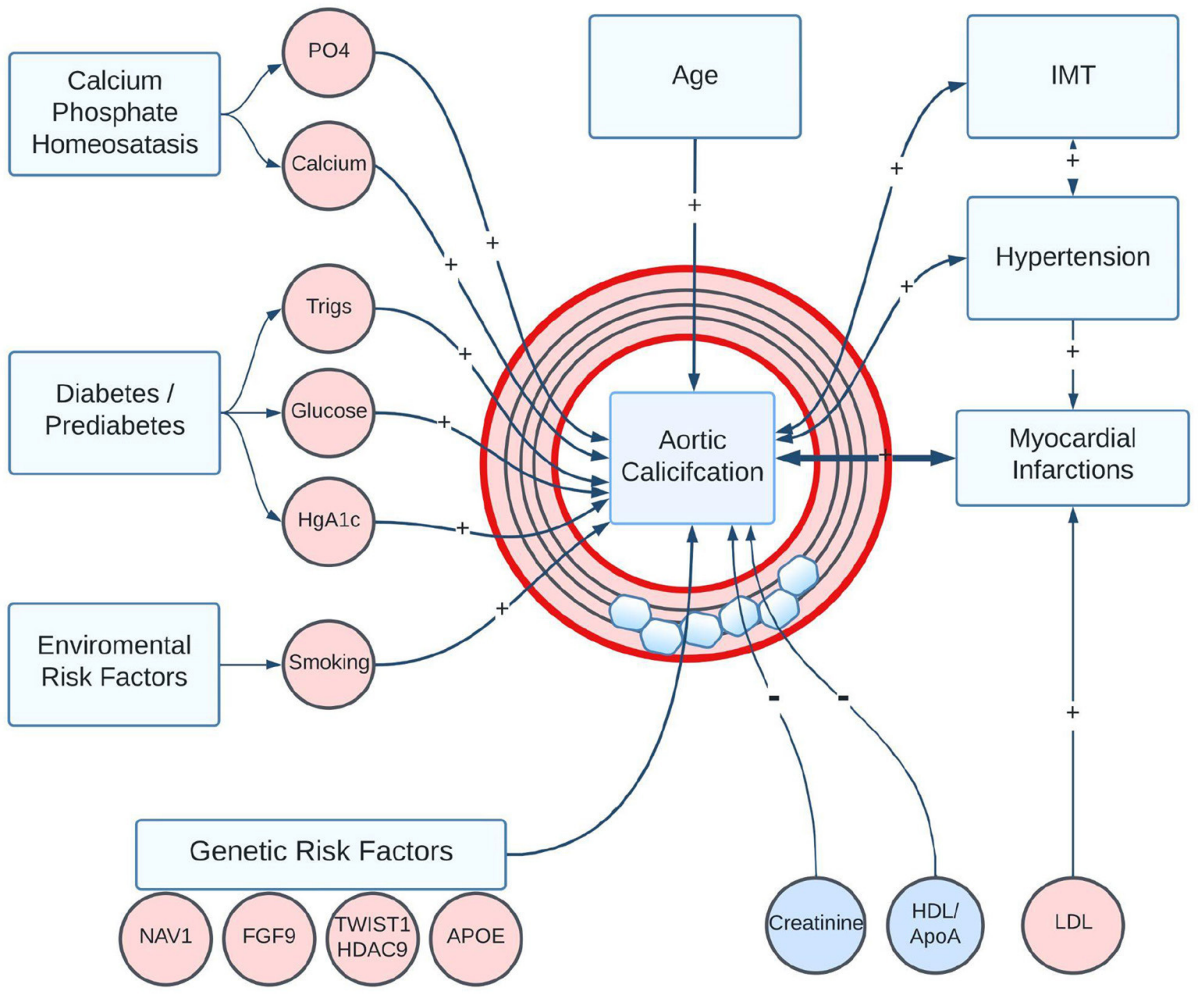
Summary of statistically significant risk factors associated with the development of AAC in the UK Biobank Imaging Cohort. Positive associations are shown as “+” arrows, and negative associations are shown as “-” arrows. Age, smoking, diabetic/pre-diabetic state, and elevated plasma levels of Ca/PO4 are the biggest risk factors for the development of AAC. Notably, reduced kidney function and elevated LDL were not associated with increased AAC. A strong positive association between AAC and poor future CVD outcomes could arise through a combination of direct effects on vascular structure, indirect effects such as loss of vessel compliance and increased hypertension, or through induced correlation with calcification in other vascular sites.

The etiology of AAC is not well understood. Vascular calcification can arise as a complication of diabetes and chronic kidney disease (1); however, in this study, we find that the vast majority of individuals with calcified arteries do not display signs of kidney damage. We observed that the severity of AAC is strongly associated with elevated serum phosphate, calcium, and diabetic biomarkers, even though the levels of these biomarkers would be considered within clinically normal ranges. The association of normal but elevated phosphate to AAC is consistent with similar observations made in the Framingham Offspring Study (37) that the risk of cardiovascular disease increases with phosphate levels even in the absence of hyperphosphatemia. It is plausible that over the lifetime of individuals even small but systemic elevation of phosphate contributes to the development of AAC. An overall summary of the core identified associations is illustrated in Figure 11.

Two distinct forms of arterial calcification are known to exist—intima layer calcification associated with plaque deposits and medial layer calcification (38). The resolution of DEXA images is not sufficient to distinguish between different forms of calcification and it is possible that AAC has a shared component of both. In this study, we did not find a statistically significant association between AAC and LDL. We believe we were sufficiently powered with ~38K observations to find an association if it existed; thus, it is likely to conclude that hypercholesterolemia is not a contributing factor to AAC. We further considered whether the lack of association might be due to confounding with statins usage. In every statin-adjusted LDL model, we observed that AAC was an independent contributor to MI outcomes, further supporting the hypothesis that AAC is unlikely to be a plaque-driven phenomenon. Considering the elevated phosphate association, we believe that our observations are more consistent with the medial layer calcification hypothesis, although confirmation would require large-scale histological evaluation.

The other plausible explanation for the development of AAC is genetic risk factors. It has been observed that smooth muscle cells transform into an osteoblast-like state and hydroxyapatite is progressively deposited across various parts of the cardiovascular system including heart valves, coronary arteries, aorta, abdominal aorta, tibial arteries, and kidneys (39, 40). In support of this “phenotypic-switch” hypothesis, we saw a robust replication of an AAC association at the *TWIST1/HDAC9* locus. Investigation of which of these genes potentiate osteogenic states in the vasculature is an active research area, with experimental evidence supporting the role of both (19, 41).

Overall, our findings highlight the importance of monitoring calcification in large arteries. The contribution of AAC to cardiovascular disease risk is similar to that of hypercholesterolemia, but the condition remains severely underdiagnosed. Although there are currently no medical interventions that could be used to treat or slow the progression of calcification, there is ongoing work to develop therapeutics in this space (42). Based on the evidence collected here, we believe that anti-calcification therapies would broadly be helpful, and likely would be complementary to existing lipid-lowering strategies in reducing cardiovascular risk.

## Data availability statement

Data used in this research can be obtained by qualified researchers by application from the UK Biobank. Summary statistics for the genome-wide association study conducted in the UK Biobank cohort are available from the NHGRI-EBI GWAS catalog under accession number GCST90134614. Summary statistics for the genome-wide association study can be obtained by application to dbGaP accession phs000930.v9.p.

## Ethics statement

The studies involving human participants were reviewed and approved by UKBB. Data were accessed under the approval of UKBB within project 18448. The study was conducted following the principles of the declaration of Helsinki and all participants gave prior written informed consent to participate in this study.

## Author contributions

AS and EM carried out epidemiological analysis. DT, ES, and MC carried out genetic analysis. JR and JV developed machine learning models. All authors contributed to the article and approved the submitted version.

## Funding

This work was supported by Calico Life Sciences LLC. Support for the CHARGE consortium infrastructure was provided by the NIH grant R01 HL105756 (B Psaty). Support for establishing and curation of the dbGaP CHARGE Summary site (phs000930) was provided by the University of Virginia. The funders were not involved in the study design, collection, analysis, interpretation of data, the writing of this article or the decision to submit it for publication.

## Conflict of interest

Authors AS, JR, ES, MC, and EM are employed by Calico Life Sciences LLC. Authors DT and JV were employed by Calico Life Sciences LLC. The remaining authors declare that the research was conducted in the absence of any commercial or financial relationships that could be construed as a potential conflict of interest.

## Publisher's note

All claims expressed in this article are solely those of the authors and do not necessarily represent those of their affiliated organizations, or those of the publisher, the editors and the reviewers. Any product that may be evaluated in this article, or claim that may be made by its manufacturer, is not guaranteed or endorsed by the publisher.

## Abbreviations

HbA1c: glycated hemoglobin
GGT: gamma glutamyl transferase
ApoA: apolipoprotein
HDL: high density lipoprotein
LDL: low density lipoprotein
WBC: White blood cell
MCV: mean corpuscular volume
MCHC: mean corpuscular hemoglobin concentration
RBC, red blood cell, Imm. Reticulocyte: immature reticulocyte
IMT: intima media thickness
SBP: systolic blood pressure
PEF: peak expiratory flow
FVC: forced vital capacity
QUI, quantitative ultrasound index, US: ultrasound
BMD: bone mineral density
FEV1: forced expiratory volume in 1 second
CVD: Cardiovascular Disease
CT: Computed tomography
DEXA: Dual-Energy X-ray Absorptiometry
BMD: bone mineral density
CKD: Chronic Kidney Disease
MI: Myocardial Infarction
eGFR: Estimated Glomerular Filtration rates
CHARGE Consortium: Cohorts for Heart and Aging Research in Genomic Epidemiology
MrOS: The Osteoporotic Fractures in Men Study.
