# Supplementary material for: Calcification of the abdominal aorta is an under-appreciated cardiovascular disease risk factor in the general population
Source: Front Cardiovasc Med. 2022 Oct 6;9:1003246. doi: 10.3389/fcvm.2022.1003246 (PMC9582957; doi:10.3389/fcvm.2022.1003246)
Supplement: Supplementary file 1 [file Data_Sheet_1.pdf]

AAC Analysis Supporting Figures and Supplementary Methods

Supplementary Figures **2**

Figure S1: Distribution of variability in calcification scores between four annotators. 2

Figure S2: Reproducibility of Manually Annotated Calcification Scores 3

Figure S3: Poor performance of convenation non-segmented ML Model 4

Figure S4: Segmentation Pipeline Overview (Pipeline #1) 5

Figure S5: Accuracy of segmentation approach on spine segmentation task 7

Figure S8: Object Detection Pipeline Overview (Pipeline #2) 9

Figure S9: Performance of Object Detection ML Pipeline #2 10

Figure S10: Bland Altman Plot. 12

Figure S11: Association of AAC environmental risk factors. 13

Figure S12 Association of AAC with plasma biomarkers. 14

Figure S13: Association of Blood Biochemistry Measures with AAC in UKBB and MrOS Cohorts 16

Figure S14: Association of AAC with CBC measures. 17

Figure S15: Association of AAC with physiological parameters. 20

Figure S16: Kidney and Diabetic diseases function measures stratified by AAC. 21

Figure S17: Association with AAC with glomerular filtration rate. 22

Figure S18:   Heritability Enrichment 23

Figure S19: Genetic colocalization analysis with tissue expression 24

Figure S20: Genetic colocalization analysis with physiological and disease measures. 25

Figure S21: TWIST1 transcript expression in aortic single cell data 26

Figure S22: Rare variant analysis 27

Figure S23: Effect of statins on LDL-Blood Pressure relationship. 29

Figure S24: Myocardial infarction model of LDL, statins, and aortic calcification. 30

Figure S25: Consistency of LDL imputation procedure for comparing LDL risk to Calcification risk. 32

Figure S26: CoxPH association of AAC and LDL for CVD events post imaging visit. 33

Supplementary Methods **34**

Machine Learning Methods: 34

Machine Learning Pipeline#1 34

Detection of the Vertebrae Using Segmentation 34

Aortic Region Extraction 35

Regression 35

Machine Learning Pipeline#2 36

Detection of the Vertebrae Using Segmentation 36

Aortic Region Extraction 37
Regression 38
Ensemble Model 38
Biomarker Analysis Methods 39
Survival Analysis Methods 39
Comparison of risk from Aortic Calcification and LDL 39
Genetics Methods 40
Rare variant association study (RVAS) 40
Common variant genome wide association study (GWAS) 40
GWAS Meta-analysis 40
Genetic architecture of AAC 41
Identification of distinct association signals 41
Construction of genetic credible sets 41
Heritability estimates 42
Genetic correlations 42
Partitioning of AAC heritability 42
Genetic colocalization of AAC with other phenotypes 43
Functional Analysis of GWAS Hits 43
Follow up analysis at the rs2107595 locus 43
References **45**

Supplementary Figures

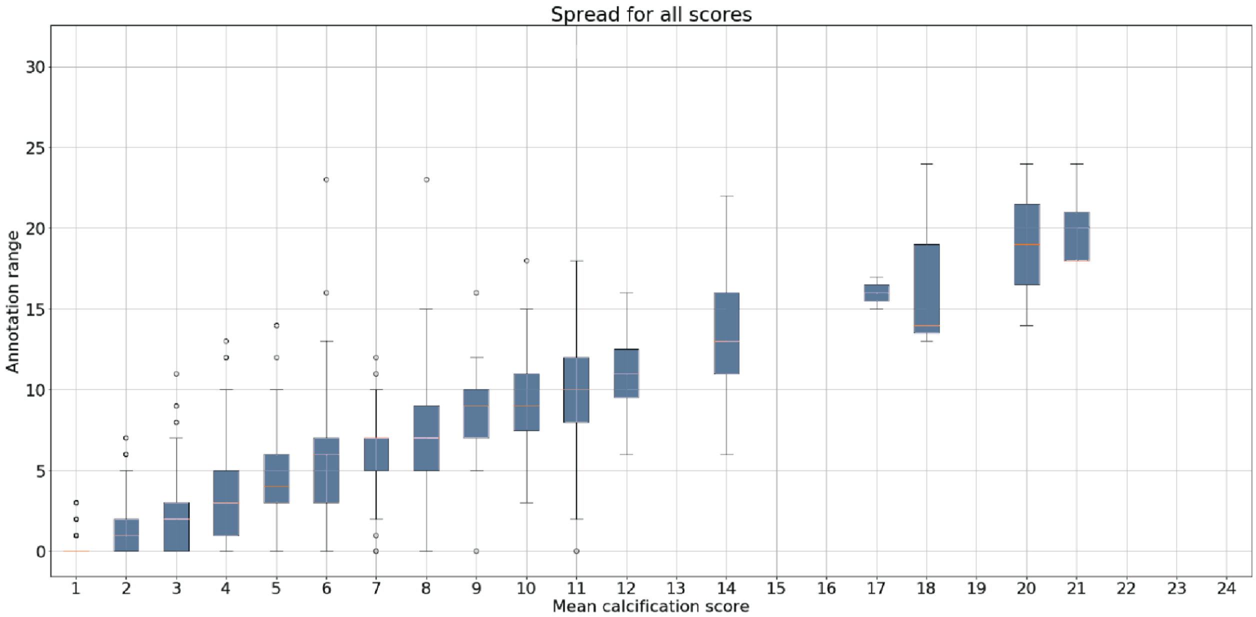

Figure S1: Distribution of variability in calcification scores between four annotators.

There is strong agreement between annotations in assignment of calcification value. The task becomes harder in the more severe cases, and the variability increases as the severity of calcification increases (n=1000).

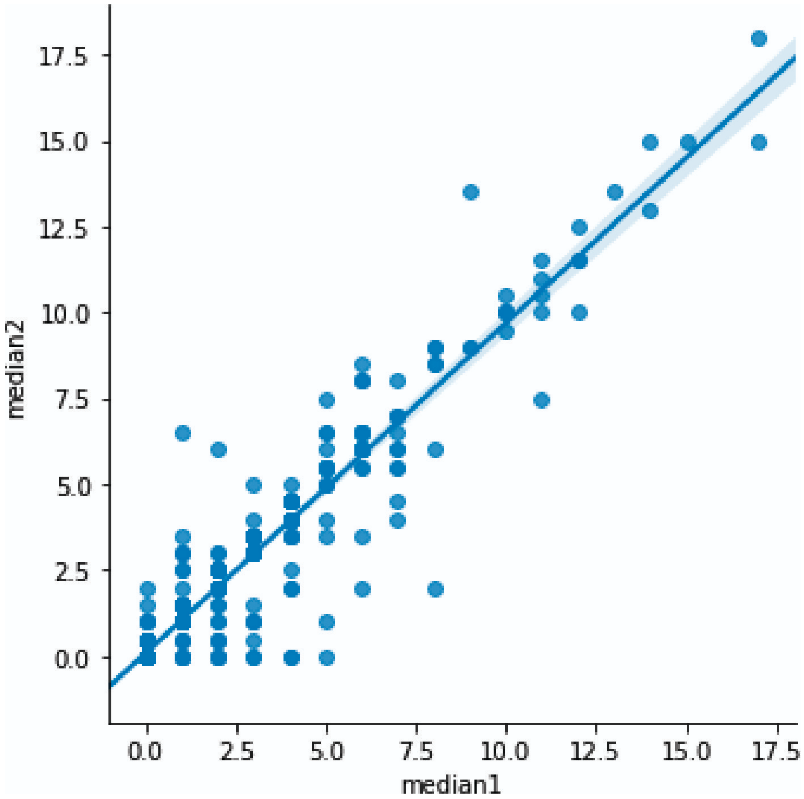

Figure S2: Reproducibility of Manually Annotated Calcification Scores

The median scores for 136 manually annotated scans from two rounds of annotation are highly correlated.

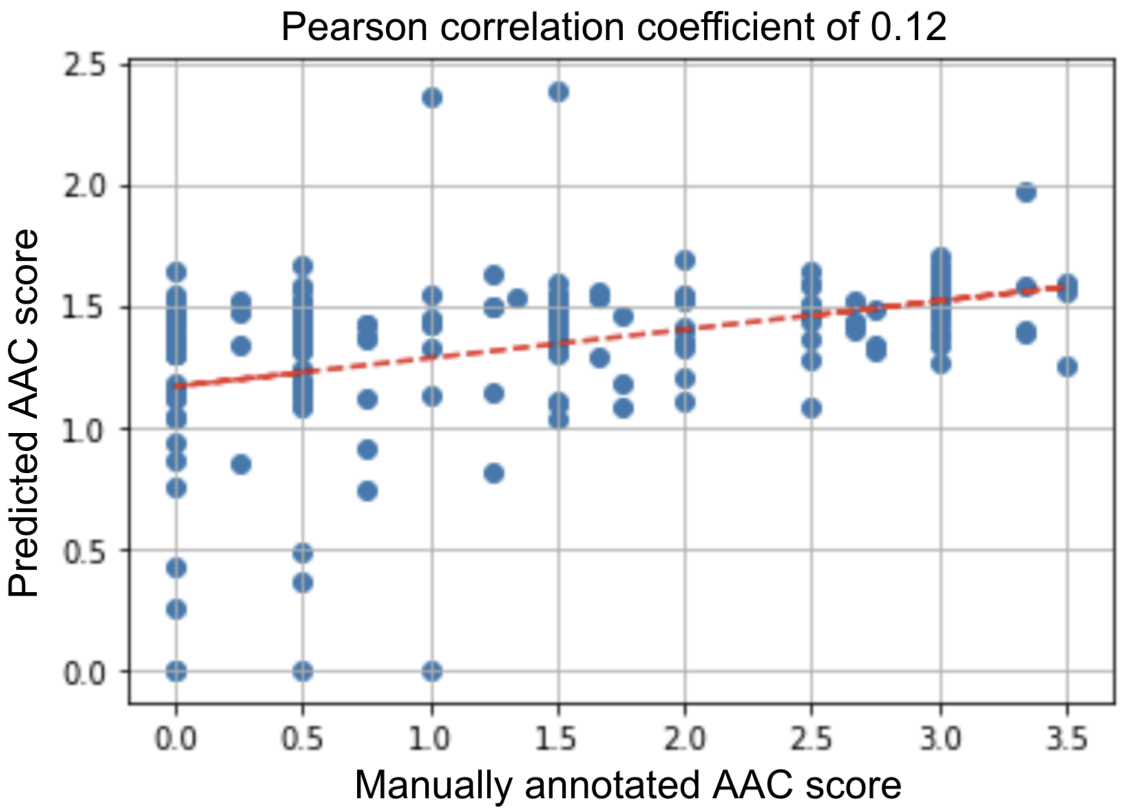

Figure S3: Poor performance of convenation non-segmented ML Model

Conventional deep learning regression approaches with ResNet50 architecture performed poorly on a test set while quantifying aortic calcification from the complete lumbar spine DEXA scans (n=300).

A.

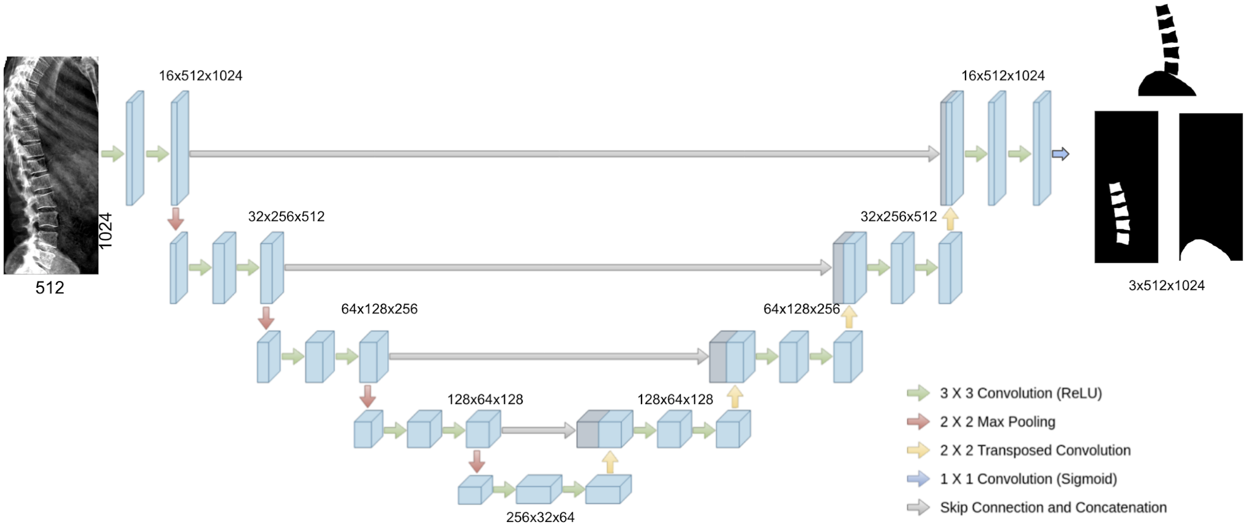

B.

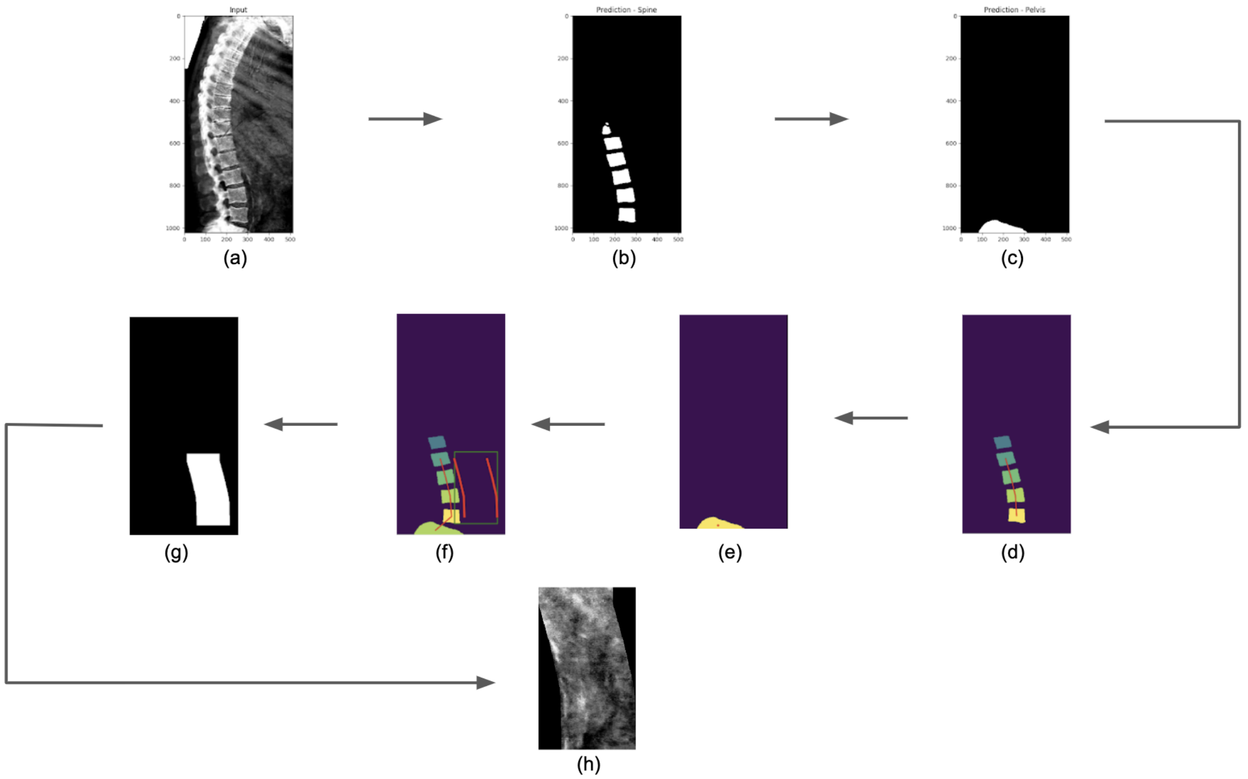

Figure S4: Segmentation Pipeline Overview (Pipeline #1)

A. U-net architecture used for segmenting relevant spine and pelvis regions in pipeline. The input DEXA image is shown on the left and the binary masks for the three output classes - background, lower spine and pelvis - are shown on the right.

B. Schematic representation of the steps to extract abdominal aortic region from lumbar spine DEXA scans. (a) shows the input image. (b)-(c) show the predicted segmentation masks of the lower spine and the pelvis. (d)-(f) show the predicted centroids of the different vertebrae (connected), predicted centroid of the pelvis and the overall spinal curvature together with the estimated aortic region to the right of the spine. (g) shows the binary mask of the predicted aortic region. (h) shows the aortic region extracted from the original input image. This is then fed into a regression model to score the calcification level.

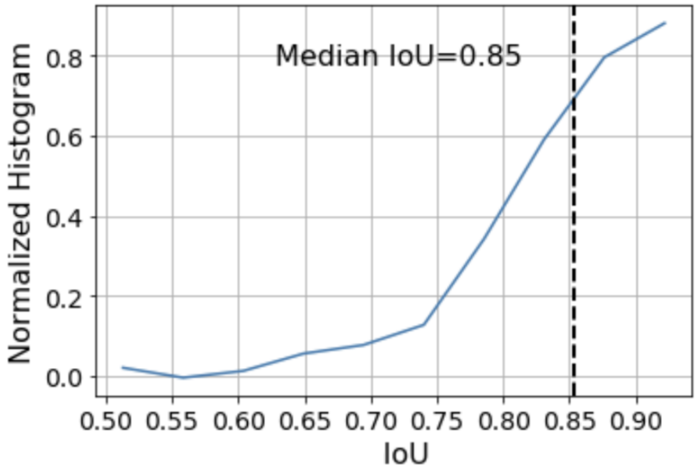

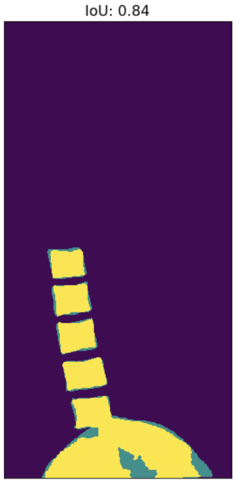

Figure S5: Accuracy of segmentation approach on spine segmentation task

A. Probability density of IoU values obtained across the test set (n=300). The median IoU value is shown with a dashed line. Exactly 50% of all images in the datasets had IoU (intersection over union) score of above 0.85.

B. shows an overlay of the ground truth segmentation mask and the predicted segmentation mask for a typical example image whose IoU is close to the median value. The yellow regions show the regions of overlap while the blue regions show gaps in the prediction mask.

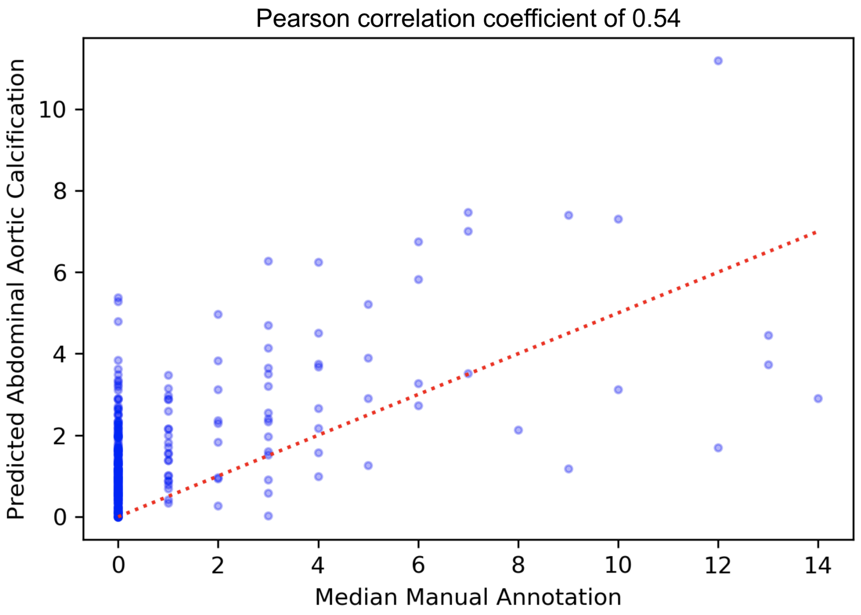

Figure S7: Accuracy of Segmentation Pipeline #1 on Validation Dataset: The predicted AAC from Segmentation Pipeline as compared to manually annotated calcification scores for the validation dataset (n=300).

A.

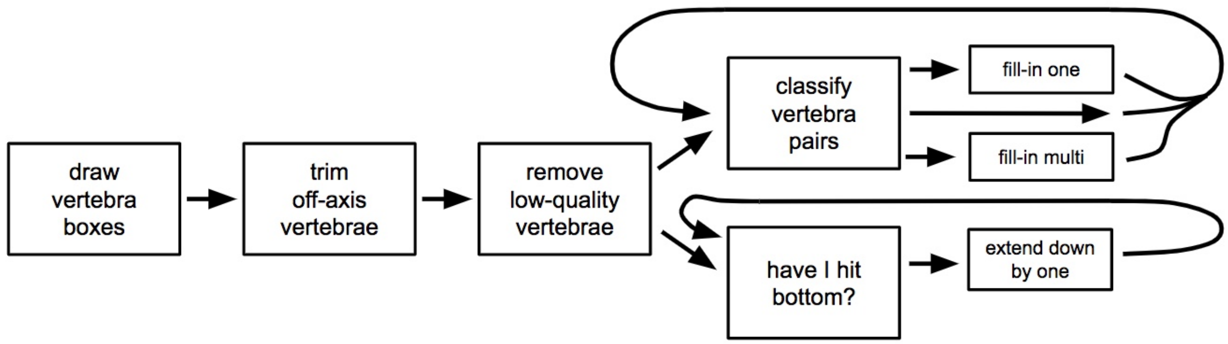

B.

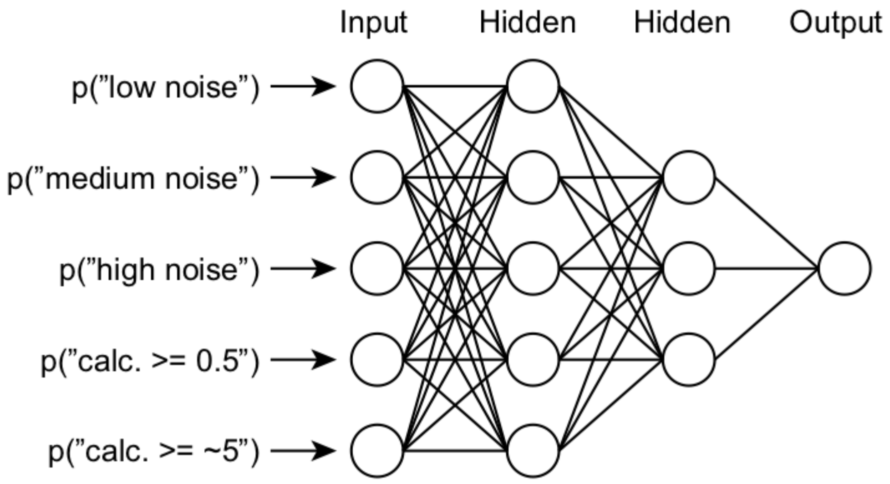

Figure S8: Object Detection Pipeline Overview (Pipeline #2)

A. Segmentation steps within machine learning pipeline to detect lumbar region vertebra.

B. Regression of aortic region next to lumbar vertebrae to aortic calcification score. Neural network was used to compare level of calcification in aorta compared to background noise in the overall image to estimate calcification score.

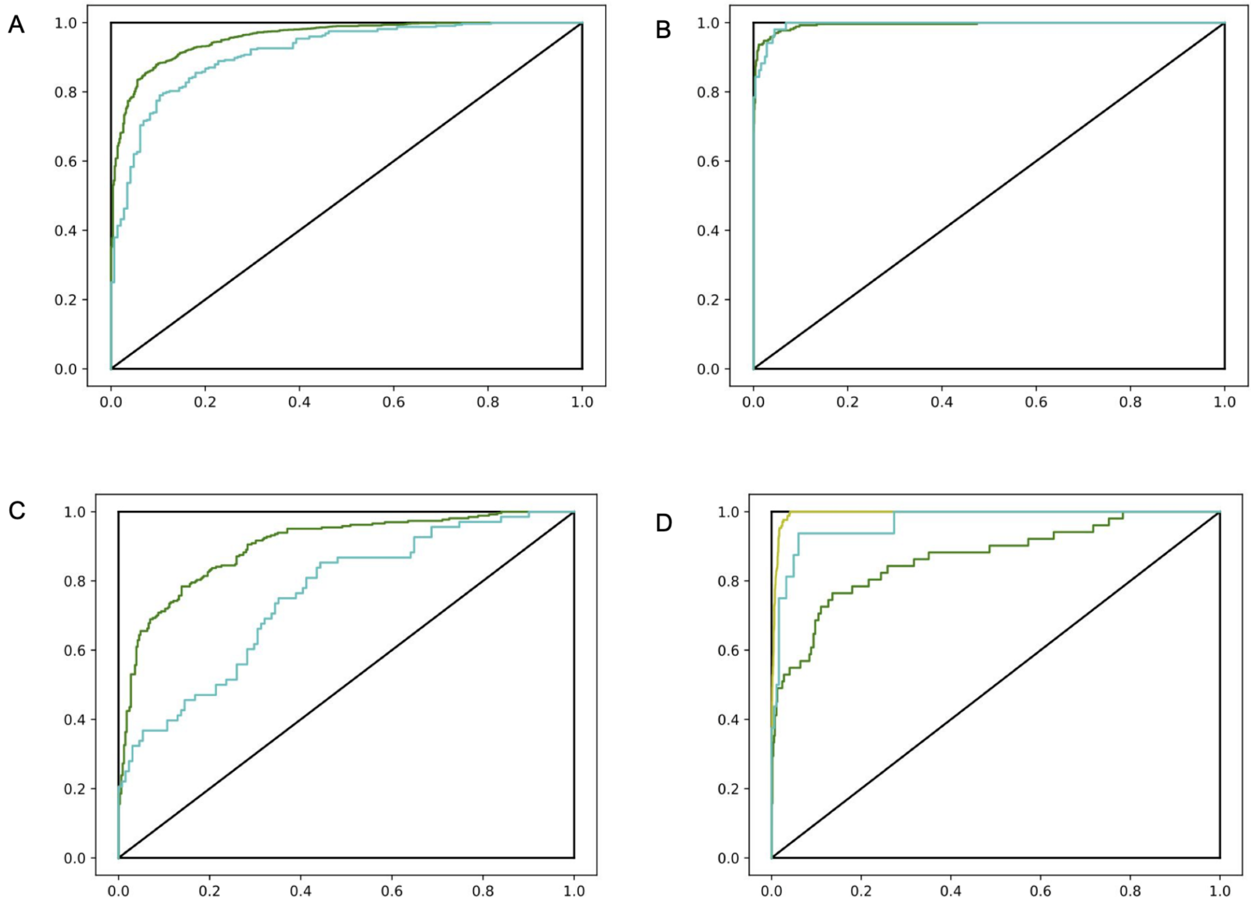

Figure S9: Performance of Object Detection ML Pipeline #2

A. Vertebrae Detection: ROC curve measuring the accuracy of the models for eliminating bad vertebrae in step b of pipeline 2 for (n=1000) training dataset (green) and (n=300) test dataset (green). Cohen's kappa values for its performance on the training set (green) and test set (blue) are 0.743 and 0.638 respectively.

B. Lumbar Region Detection: ROC curve measuring the accuracy of the models for extending vertebra to the bottom of the spine in step e of the segmentation step of pipeline 2 for training dataset (green) and test dataset (green). Cohen's kappa values for its performance on the training set (green) and test set (blue) are 0.917 and 0.874 respectively.

C. Aorta Detection: ROC curve measuring the accuracy of the model 1 that classifies calcified aorta from non-calcified aorta. Cohen's kappa values for training (green) and test sets (blue) are 0.58 and 0.33 respectively (test set, for "calcified": sensitivity = 0.75, specificity = 0.83, precision = 0.62).

D. **Calcified Region Detection:** ROC curve measuring the accuracy of the model 2 that classifies high-threshold aortic calcification. The original (n=1000) training and (n=300) test sets are shown in green and blue respectively while the sandbox enriched training set is shown in yellow in B. Cohen's kappa values for original training, sandbox

enriched training and test sets are 0.53, 0.71, and 0.73 respectively (test set, for “calcified”: sensitivity = 0.75, specificity = 0.98, precision = 0.75).

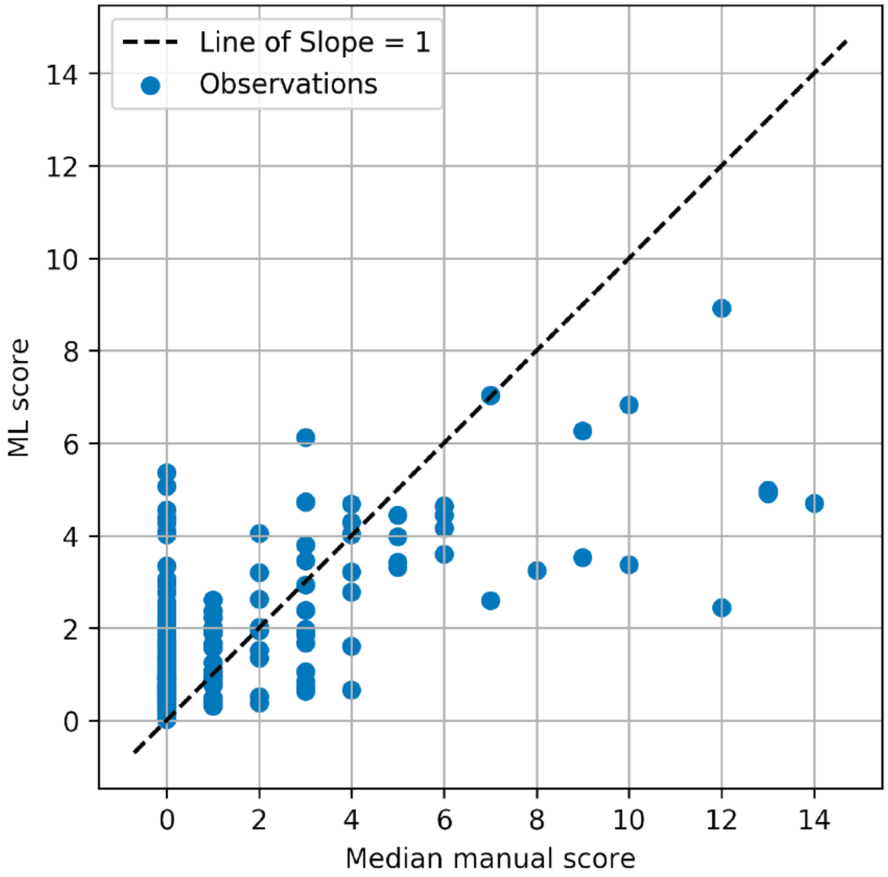

Figure S10: Bland Altman Plot.

The comparison of predicted AAC to median annotation scores for the (n=300) validation dataset from machine learning pipeline. In comparison, the mean absolute error of the manually annotated images from all four annotators varied between 0.67 and 1.45.

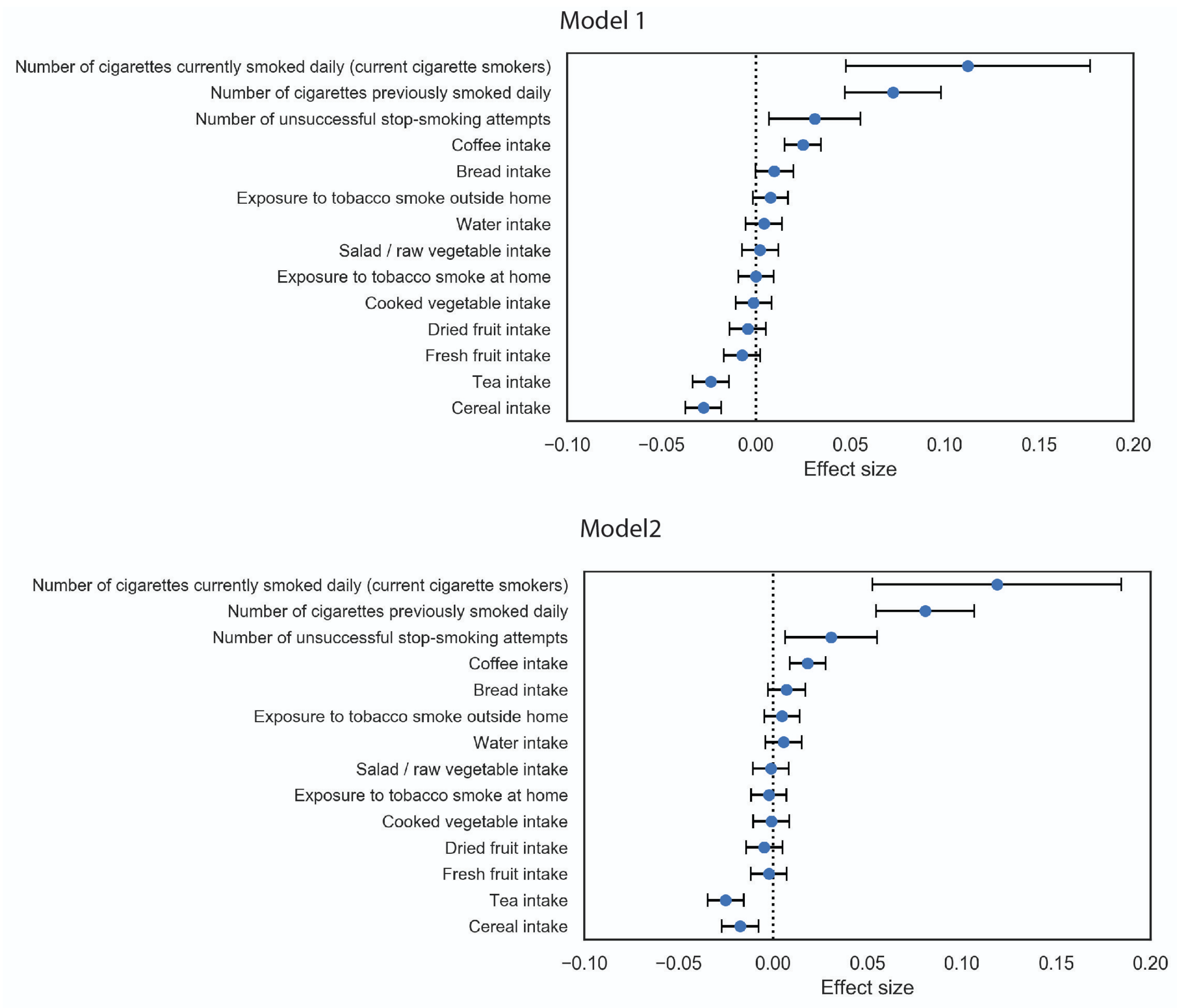

Figure S11: Association of AAC environmental risk factors.

Univariate regression analysis of risk factors at baseline for predicted AAC after adjusting for age and sex in model 1 and after adjusting for socioeconomic factors, BMI, and smoking status in addition to adjusting for age and sex in model 2 ((n=38,264). The blue dots represent mean effect size while the intervals represent standard errors for the effect size (see Methods Section 3).

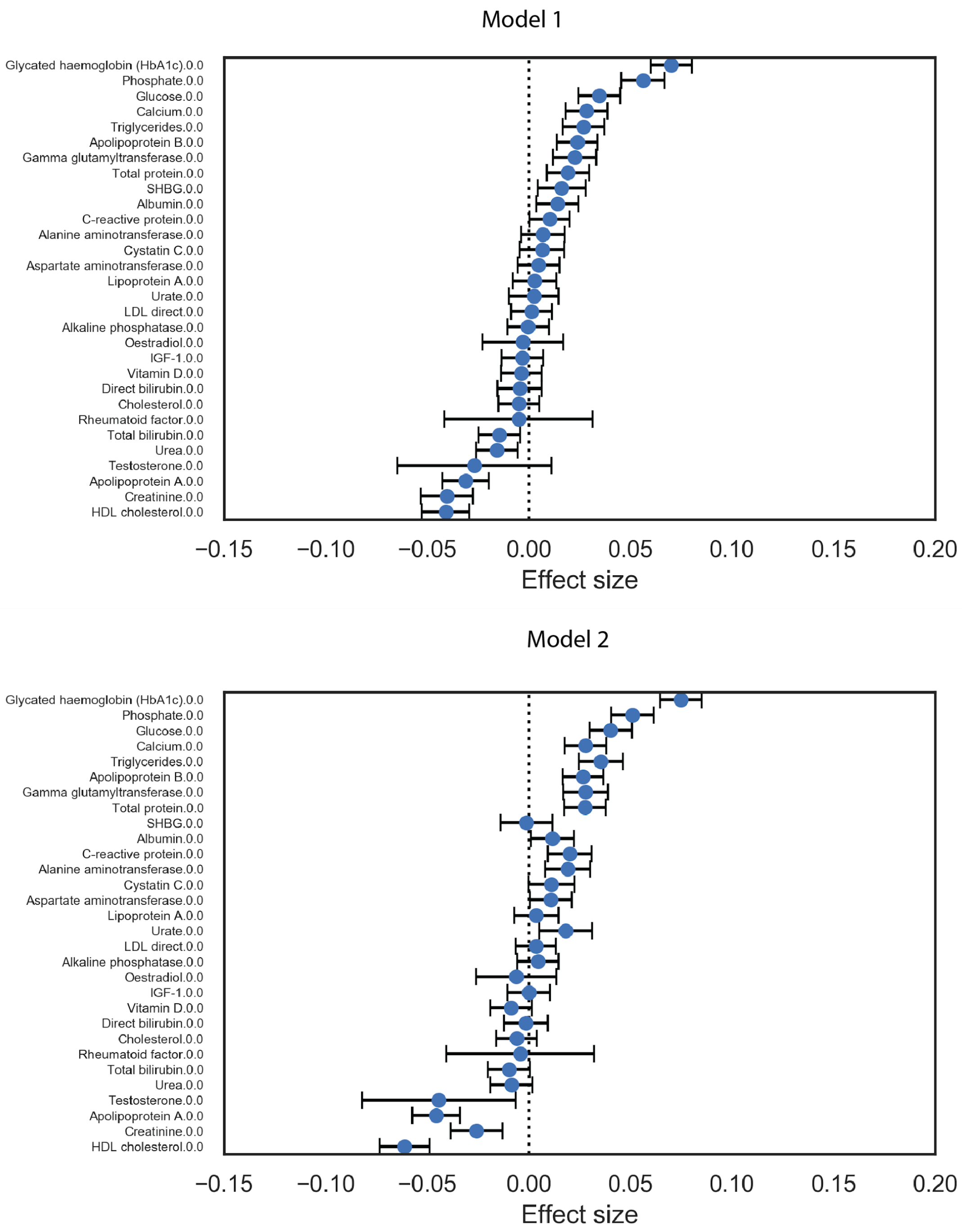

Figure S12 Association of AAC with plasma biomarkers.

Briefly, AAC is modeled as a function of each covariate adjusted for confounders (see Methods Section 3). The blue dot represents the mean effect size per standard deviation of different covariates while error bars represent 95% confidence interval. All the covariates were measured

during the baseline visit while AAC is estimated based on DEXA scans collected during the imaging visit.

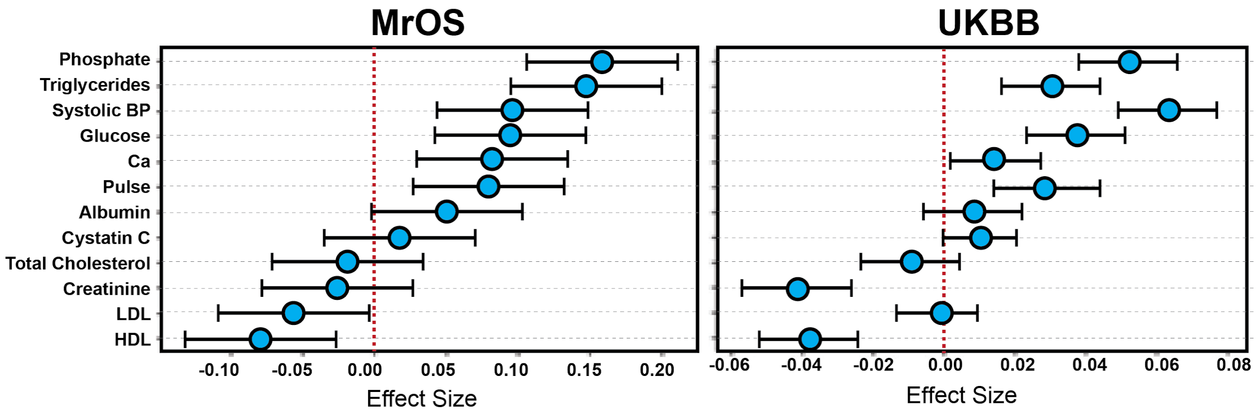

Figure S13: Association of Blood Biochemistry Measures with AAC in UKBB and MrOS Cohorts

The biomarkers that are significantly associated with AAC in MrOS cohort (n=4,800) are also significantly associated with predicted AAC in this study even though the effect sizes are slightly lower in the UK biobank cohort (n=38,264). All associations are calculated after adjusting for age and sex.

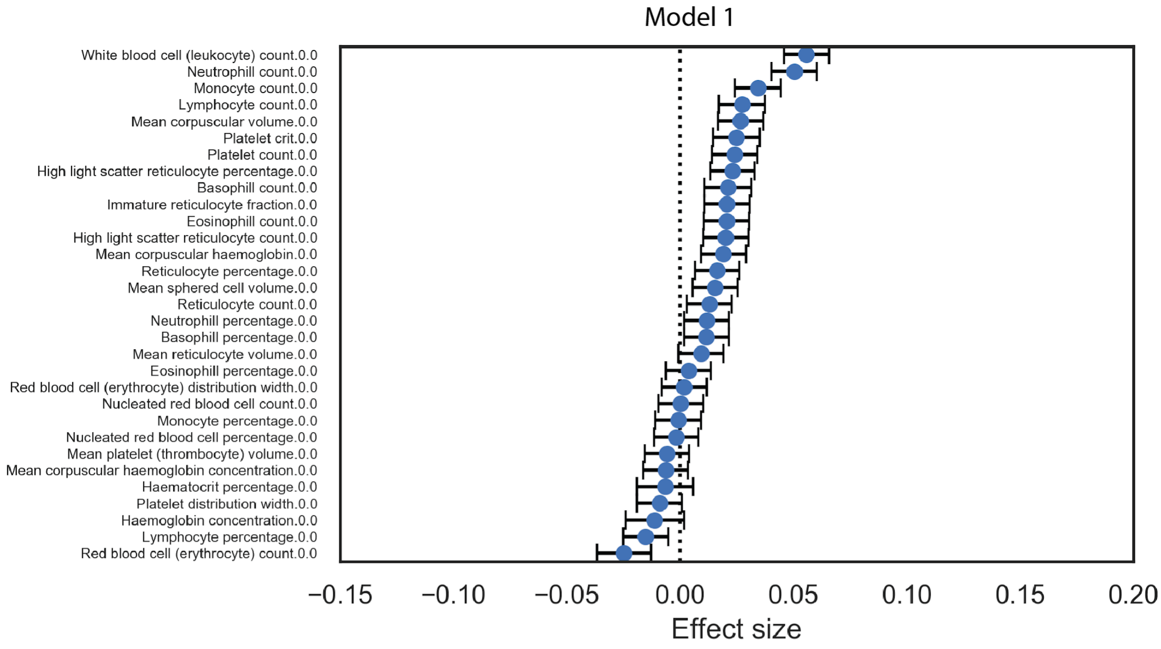

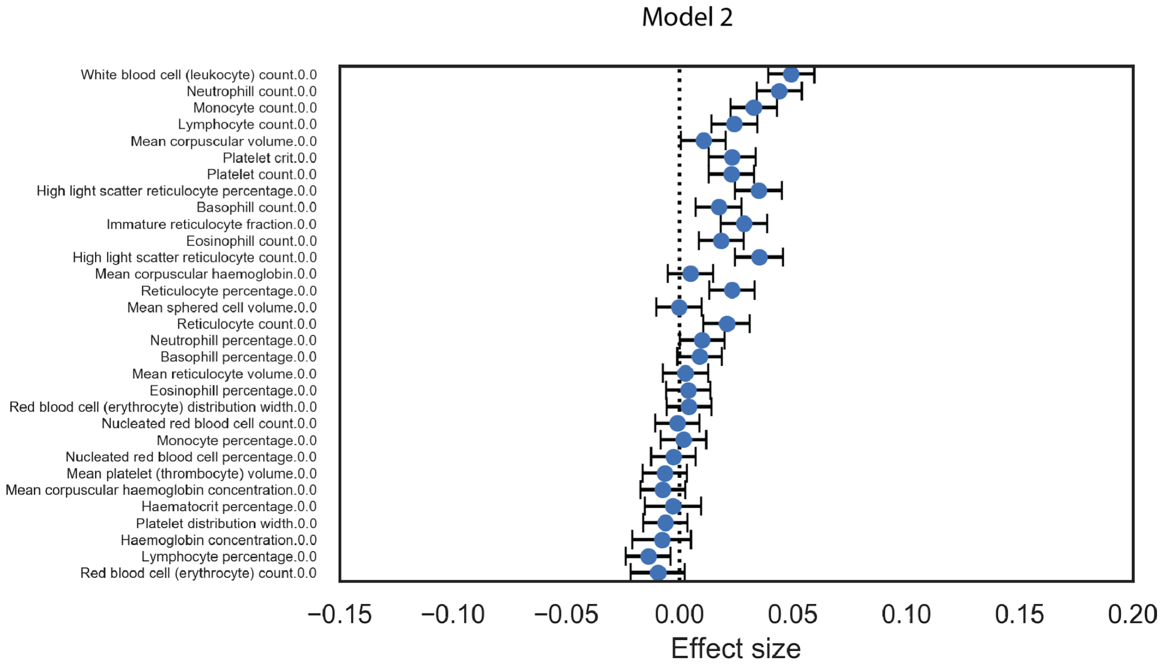

Figure S14: Association of AAC with CBC measures.

Briefly, AAC is modeled as a function of each covariate adjusted for confounders (see Methods Section 3). The blue dot represents the mean effect size per standard deviation of different

covariates while error bars represent 95% confidence interval. All the covariates were measured during the baseline visit while AAC is estimated based on DEXA scans collected during the imaging visit.

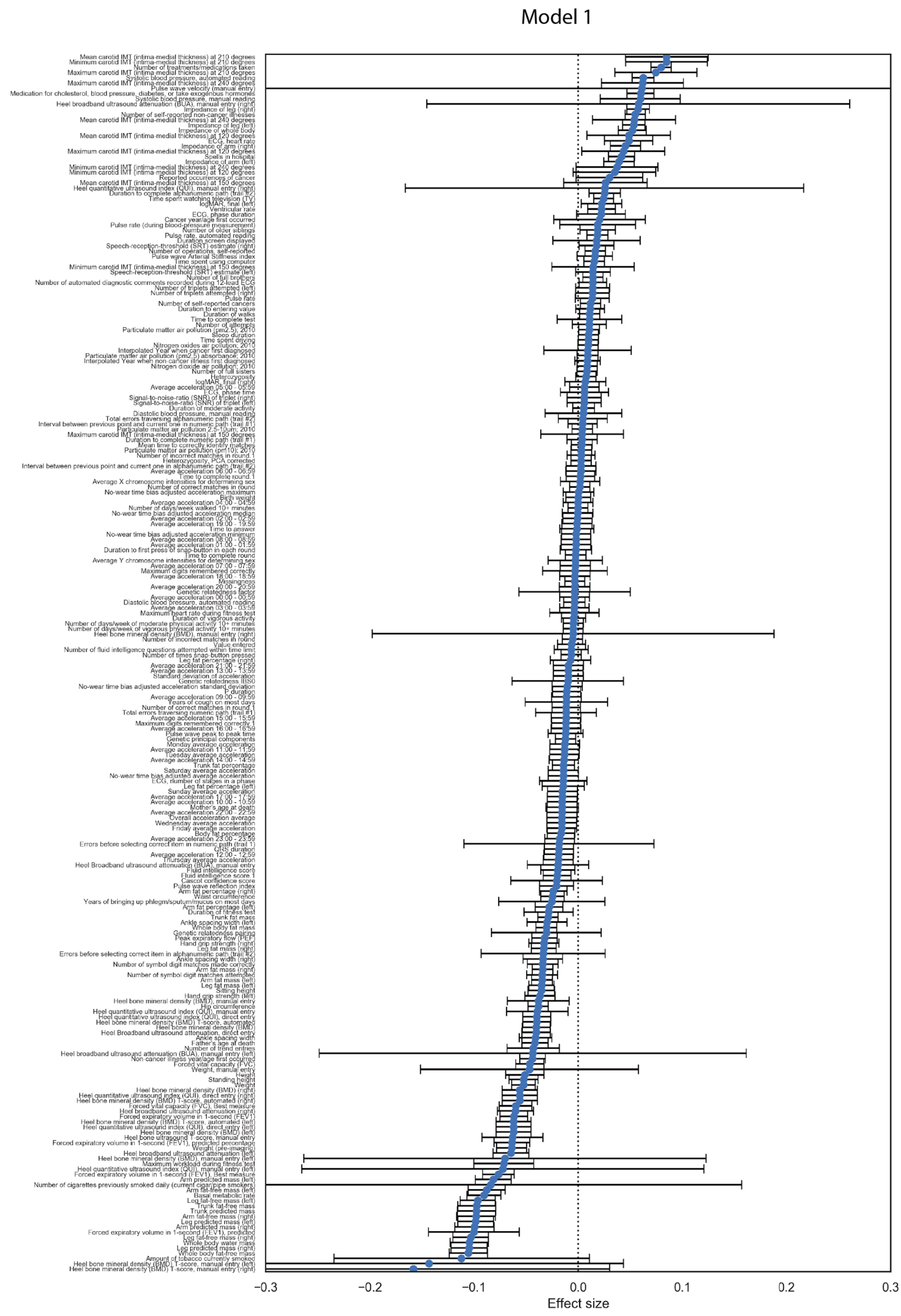
Model 1
Mean carotid IMT (intima-medial thickness) at 210 degrees
Minimum carotid IMT (intima-medial thickness) at 210 degrees
Number of treatments/medications taken
Maximum carotid IMT (intima-medial thickness) at 210 degrees
Systolic blood pressure, automated reading
Maximum carotid IMT (intima-medial thickness) at 240 degrees
Pulse wave velocity (manual entry)
Medication for cholesterol, blood pressure, diabetes, or take exogenous hormones
Systolic blood pressure, manual reading
Heel broadband ultrasound attenuation (BUA), manual entry (right)
Impedance of leg (right)
Number of self-reported non-cancer illnesses
Mean carotid IMT (intima-medial thickness) at 240 degrees
Impedance of leg (left)
Impedance of whole body
Mean carotid IMT (intima-medial thickness) at 120 degrees
ECG, heart rate
Impedance of arm (right)
Maximum carotid IMT (intima-medial thickness) at 120 degrees
Spells in hospital
Impedance of arm (left)
Minimum carotid IMT (intima-medial thickness) at 240 degrees
Minimum carotid IMT (intima-medial thickness) at 120 degrees
Reported occurrences of cancer
Mean carotid IMT (intima-medial thickness) at 150 degrees
Heel quantitative ultrasound index (QUI), manual entry (right)
Duration to complete alphanumeric path (trail #2)
Time spent watching television (TV)
logMAR, final (left)
Ventricular rate
ECG, phase duration
Cancer year/age first occurred
Pulse rate (during blood-pressure measurement)
Number of older siblings
Pulse rate, automated reading
Duration screen displayed
Speech-reception-threshold (SRT) estimate (right)
Number of operations, self-reported
Pulse wave Arterial Stiffness index
Time spent using computer
Minimum carotid IMT (intima-medial thickness) at 150 degrees
Speech-reception-threshold (SRT) estimate (left)
Number of full brothers
Number of automated diagnostic comments recorded during 12-lead ECG
Number of triplets attempted (left)
Number of triplets attempted (right)
Pulse rate
Number of self-reported cancers
Duration to entering value
Duration of walks
Time to complete test
Number of attempts
Particulate matter air pollution (pm2.5); 2010
Sleep duration
Time spent driving
Nitrogen oxides air pollution; 2010
Interpolated Year when cancer first diagnosed
Particulate matter air pollution (pm2.5) absorbance; 2010
Interpolated Year when non-cancer illness first diagnosed
Nitrogen dioxide air pollution; 2010
Number of full sisters
Heterozygosity
logMAR, final (right)
Average acceleration 05:00 - 05:59
ECG, phase time
Signal-to-noise-ratio (SNR) of triplet (right)
Signal-to-noise-ratio (SNR) of triplet (left)
Duration of moderate activity
Diastolic blood pressure, manual reading
Total errors traversing alphanumeric path (trail #2)
Interval between previous point and current one in numeric path (trail #1)
Particulate matter air pollution 2.5-10um; 2010
Maximum carotid IMT (intima-medial thickness) at 150 degrees
Duration to complete numeric path (trail #1)
Mean time to correctly identify matches
Particulate matter air pollution (pm10); 2010
Number of incorrect matches in round.1
Heterozygosity, PCA corrected
Interval between previous point and current one in alphanumeric path (trail #2)
Average acceleration 06:00 - 06:59
Time to complete round.1
Average X chromosome intensities for determining sex
Number of correct matches in round
No-wear time bias adjusted acceleration maximum
Birth weight
Average acceleration 04:00 - 04:59
Number of days/week walked 10+ minutes
No-wear time bias adjusted acceleration median
Average acceleration 02:00 - 02:59
Average acceleration 19:00 - 19:59
Time to answer
No-wear time bias adjusted acceleration minimum
Average acceleration 08:00 - 08:59
Average acceleration 01:00 - 01:59
Duration to first press of snap-button in each round
Time to complete round
Average Y chromosome intensities for determining sex
Average acceleration 07:00 - 07:59
Maximum digits remembered correctly
Average acceleration 18:00 - 18:59
Missingness
Average acceleration 20:00 - 20:59
Genetic relatedness factor
Average acceleration 00:00 - 00:59
Diastolic blood pressure, automated reading
Average acceleration 03:00 - 03:59
Maximum heart rate during fitness test
Duration of vigorous activity
Number of days/week of moderate physical activity 10+ minutes
Number of days/week of vigorous physical activity 10+ minutes
Heel bone mineral density (BMD), manual entry (right)
Number of incorrect matches in round
Value entered
Number of fluid intelligence questions attempted within time limit
Number of times snap-button pressed
Leg fat percentage (right)
Average acceleration 21:00 - 21:59
Average acceleration 13:00 - 13:59
Standard deviation of acceleration
Genetic relatedness IBS0
No-wear time bias adjusted acceleration standard deviation
P duration
Average acceleration 09:00 - 09:59
Years of cough on most days
Number of correct matches in round.1
Total errors traversing numeric path (trail #1)
Average acceleration 15:00 - 15:59
Maximum digits remembered correctly.1
Average acceleration 16:00 - 16:59
Pulse wave peak to peak time
Genetic principal components
Monday average acceleration
Average acceleration 11:00 - 11:59
Tuesday average acceleration
Average acceleration 14:00 - 14:59
Trunk fat percentage
Saturday average acceleration
No-wear time bias adjusted average acceleration
ECG, number of stages in a phase
Leg fat percentage (left)
Sunday average acceleration
Average acceleration 17:00 - 17:59
Average acceleration 10:00 - 10:59
Mother's age at death
Average acceleration 22:00 - 22:59
Overall acceleration average
Wednesday average acceleration
Friday average acceleration
Body fat percentage
Average acceleration 23:00 - 23:59
Errors before selecting correct item in numeric path (trail 1)
QRS duration
Average acceleration 12:00 - 12:59
Thursday average acceleration
Heel Broadband ultrasound attenuation (BUA), manual entry
Fluid intelligence score
Fluid intelligence score.1
Cascot confidence score
Pulse wave reflection index
Arm fat percentage (right)
Waist circumference
Years of bringing up phlegm/sputum/mucus on most days
Arm fat percentage (left)
Duration of fitness test
Trunk fat mass
Ankle spacing width (left)
Whole body fat mass
Genetic relatedness pairing
Peak expiratory flow (PEF)
Hand grip strength (right)
Leg fat mass (right)
Errors before selecting correct item in alphanumeric path (trail #2)
Ankle spacing width (right)
Number of symbol digit matches made correctly
Arm fat mass (right)
Number of symbol digit matches attempted
Arm fat mass (left)
Leg fat mass (left)
Sitting height
Hand grip strength (left)
Heel bone mineral density (BMD), manual entry
Hip circumference
Heel quantitative ultrasound index (QUI), manual entry
Heel quantitative ultrasound index (QUI), direct entry
Heel bone mineral density (BMD) T-score, automated
Heel bone mineral density (BMD)
Heel Broadband ultrasound attenuation, direct entry
Ankle spacing width
Father's age at death
Number of trend entries
Heel broadband ultrasound attenuation (BUA), manual entry (left)
Non-cancer illness year/age first occurred
Forced vital capacity (FVC)
Weight, manual entry
Height
Standing height
Weight
Heel bone mineral density (BMD) (right)
Heel quantitative ultrasound index (QUI), direct entry (right)
Heel bone mineral density (BMD) T-score, automated (right)
Forced vital capacity (FVC), Best measure
Heel broadband ultrasound attenuation (right)
Forced expiratory volume in 1-second (FEV1)
Heel bone mineral density (BMD) T-score, automated (left)
Heel quantitative ultrasound index (QUI), direct entry (left)
Heel bone mineral density (BMD) (left)
Heel bone ultrasound T-score, manual entry
Forced expiratory volume in 1-second (FEV1), predicted percentage
Weight (pre-imaging)
Heel broadband ultrasound attenuation (left)
Heel bone mineral density (BMD), manual entry (left)
Maximum workload during fitness test
Heel quantitative ultrasound index (QUI), manual entry (left)
Forced expiratory volume in 1-second (FEV1), Best measure
Arm predicted mass (left)
Number of cigarettes previously smoked daily (current cigar/pipe smokers)
Arm fat-free mass (left)
Basal metabolic rate
Leg fat-free mass (left)
Trunk fat-free mass
Trunk predicted mass
Arm fat-free mass (right)
Leg predicted mass (left)
Arm predicted mass (right)
Forced expiratory volume in 1-second (FEV1), predicted
Leg fat-free mass (right)
Whole body water mass
Leg predicted mass (right)
Whole body fat-free mass
Amount of tobacco currently smoked
Heel bone mineral density (BMD) T-score, manual entry (left)
Heel bone mineral density (BMD) T-score, manual entry (right)
−0.3
−0.2
−0.1
0.0
0.1
0.2
0.3
Effect size

Figure S15: Association of AAC with physiological parameters.

Briefly, AAC is modeled as a function of each covariate adjusted for confounders (see Methods Section 3). The blue dot represents the mean effect size per standard deviation of different covariates while error bars represent 95% confidence interval. All the covariates were measured during the baseline visit while AAC is estimated based on DEXA scans collected during the imaging visit.

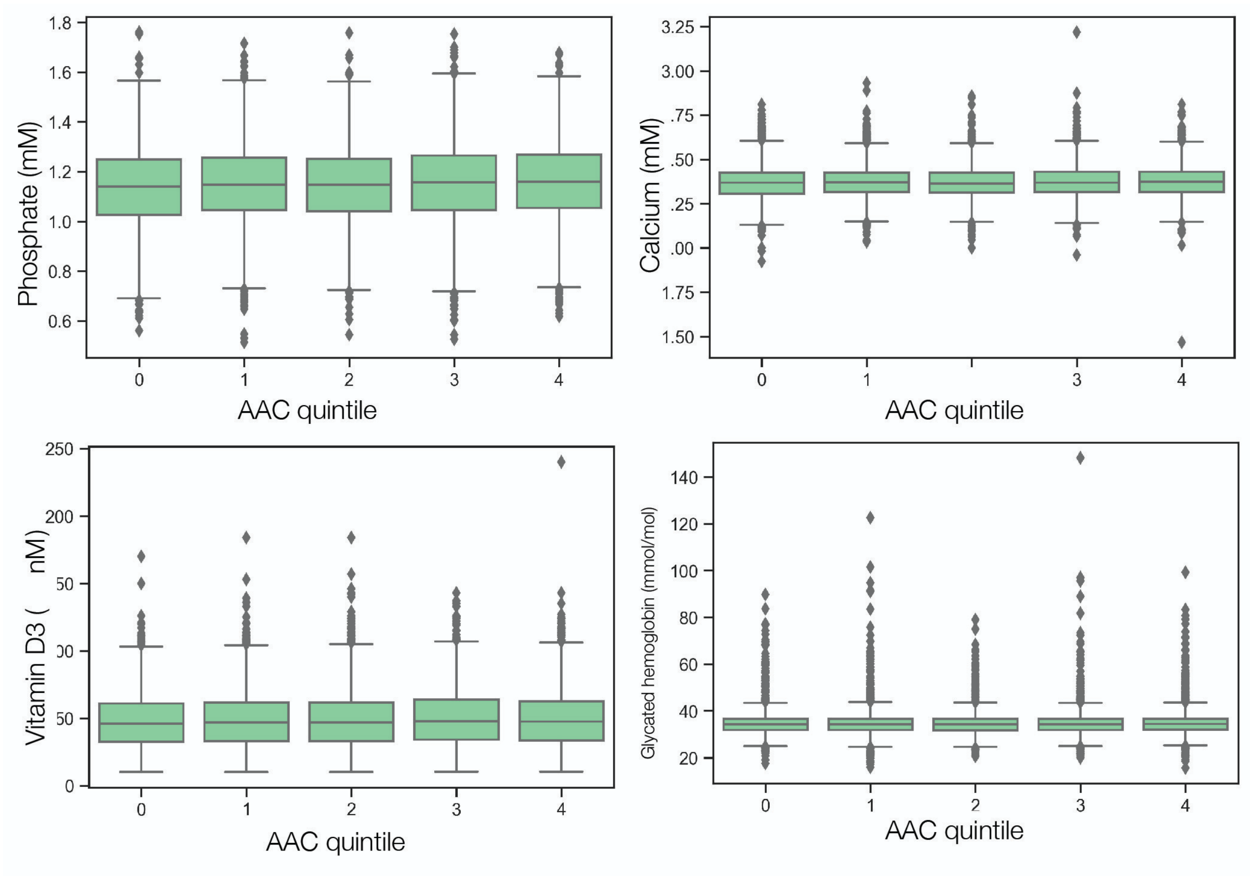

Figure S16: Kidney and Diabetic diseases function measures stratified by AAC.

Box plot showing the level of different risk factors (serum Phosphate, Calcium, Vitamin D3, and glycated Hemoglobin HbA1c) of 38,264 participants stratified according to level of calcification (higher AAC score represented by larger group number).

There is little change in the distribution of different biomarkers as a function of the AAC group (except for HbA1c levels in the highest quintile of AAC) and most participants would be within clinically healthy levels for these biomarkers.

The center line at each age represents the median biomarker level for all participants in that calcification category while the lower and upper boundaries of the box indicate the 25th and 75th percentile of the serum biomarker level for that calcification category. The lower and upper ends of the lines denote the 95% confidence interval of the serum biomarker level for that calcification category.

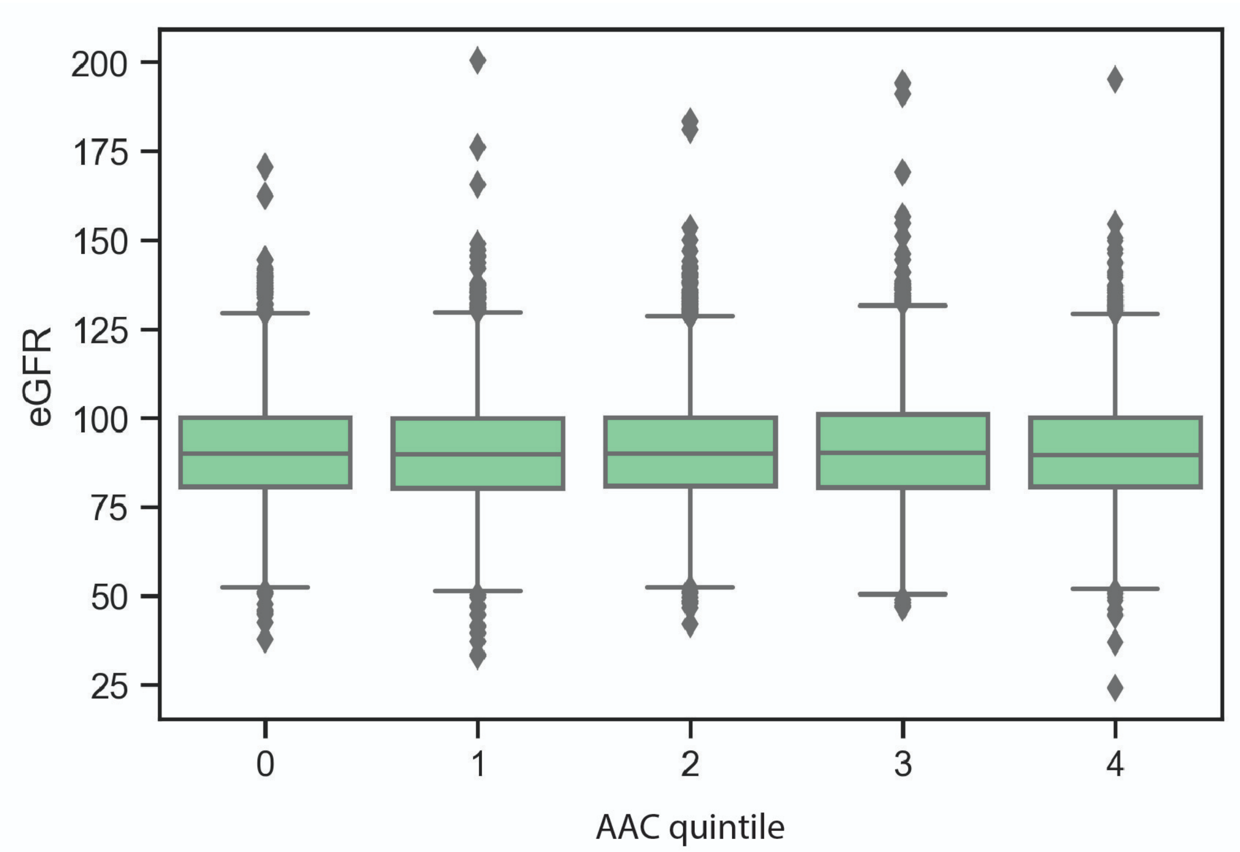

Figure S17: Association with AAC with glomerular filtration rate.

Box plot showing the variation of estimated glomerular filtration rate (eGFR) of 38,264 participants grouped according to level of (higher AAC score represented by larger group number). The eGFR was calculated from Cystatin-C levels using the formula (1). The eGFR for the participants with the highest levels of AAC (categories 3 and 4) tend to be lower, indicating that their kidneys are not functioning as efficiently as the other participants on average. The center line at each age represents the median biomarker level for all participants in that calcification category while the lower and upper boundaries of the box indicate the 25th and 75th percentile of the eGFR for that calcification category. The lower and upper ends of the lines denote the 95% confidence interval of the eGFR for participants within a particular calcification category.

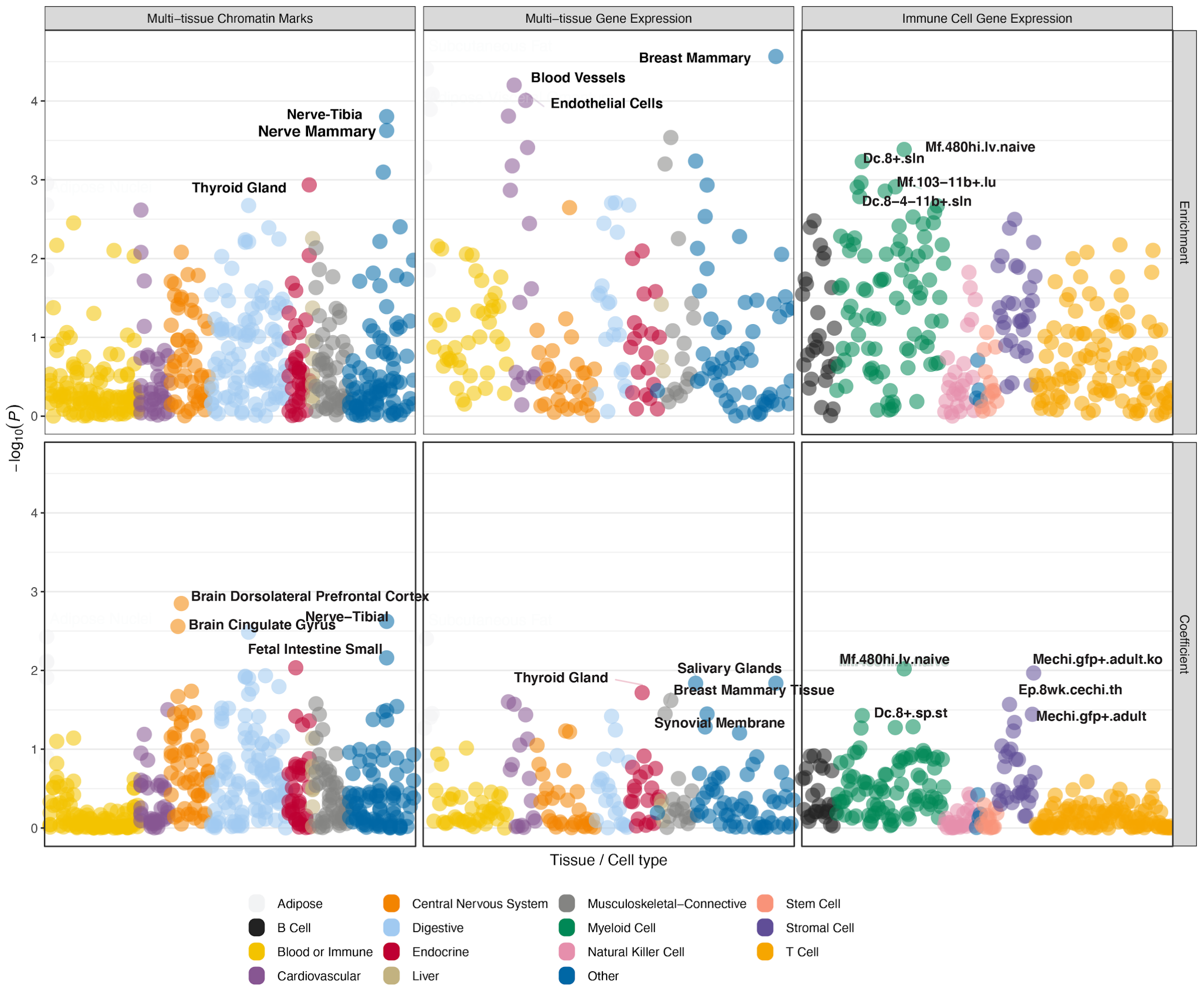

Figure S18: Heritability Enrichment

Heritability enrichment of AAC across different genomic annotations.
P-values (y-axis) of either total enrichment (top row facet) or positive regression coefficients (tau; bottom row facet) across annotations (x-axis) and datasets (column facets). Enrichment is calculated by dividing the proportion of heritability explained by SNPs in an annotation by proportion of SNPs in an annotation ($\text{proportion}_{h2}/\text{proportion}_{SNPs}$). Regression coefficients represent the average contribution of an annotation to per-SNP heritability, correcting for annotations in the baseline model (see Methods Section Partition of AAC heritability).

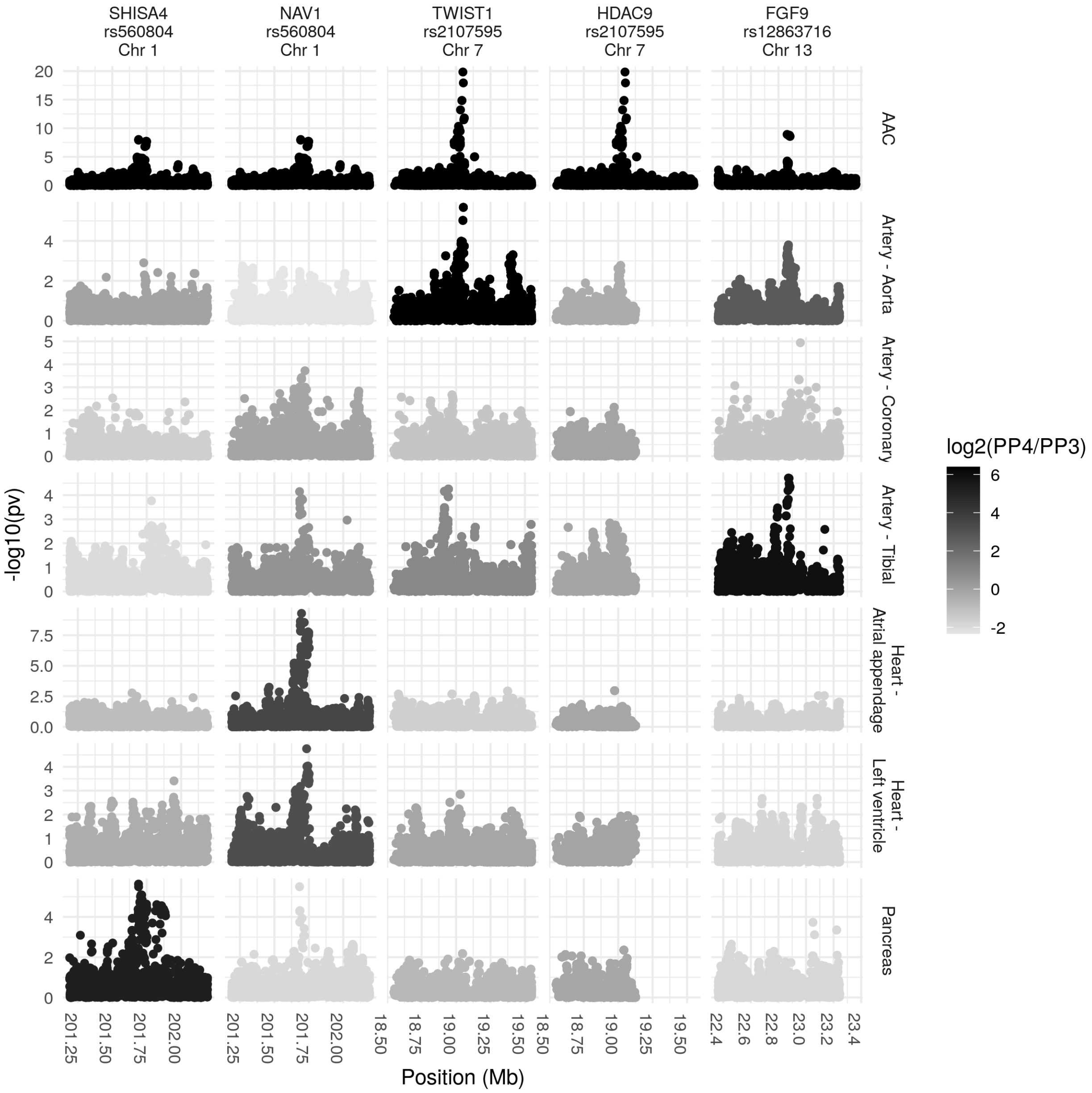

Figure S19: Genetic colocalization analysis with tissue expression

Colocalization analysis at the *NAV1/SHISA4*, *TWIST1/HDAC9*, and *FGF9* locus. Association (y-axis) of genetic variants (x-axis) with AAC and with expression of nearby genes in relevant tissues. Only the main signal at each locus is shown (see Methods section - "Genetic Colocalization with other Phenotypes")

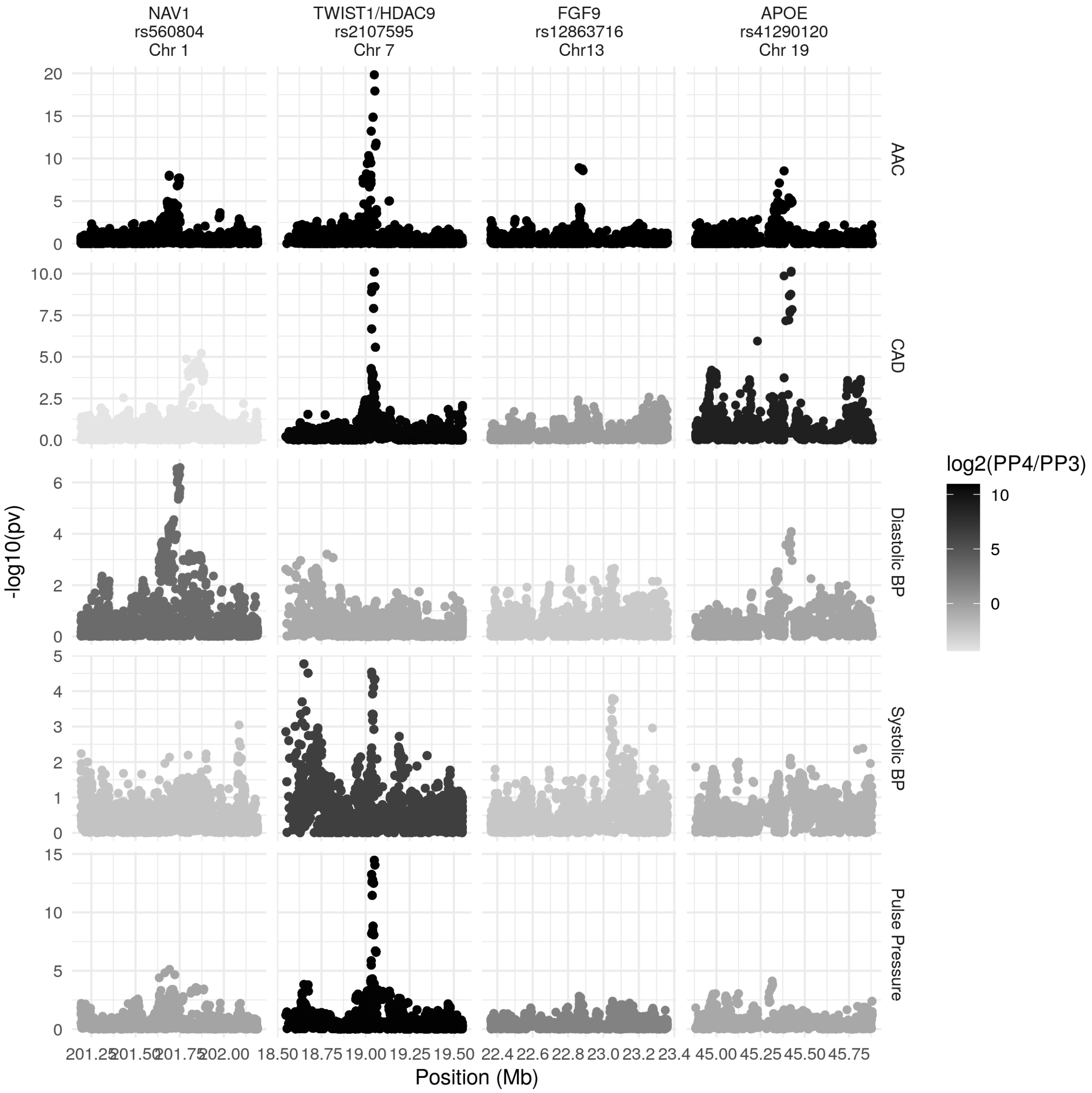

Figure S20: Genetic colocalization analysis with physiological and disease measures.

Colocalization of the lead signal at four loci where we find an association with AAC (top row) in non-UKBB cohorts. CAD=Coronary artery disease. BP=blood pressure. CAD summary statistics are from(2) and other traits are from (3) (see Methods Section Genetic Colocalization of AAC with other Phenotypes).

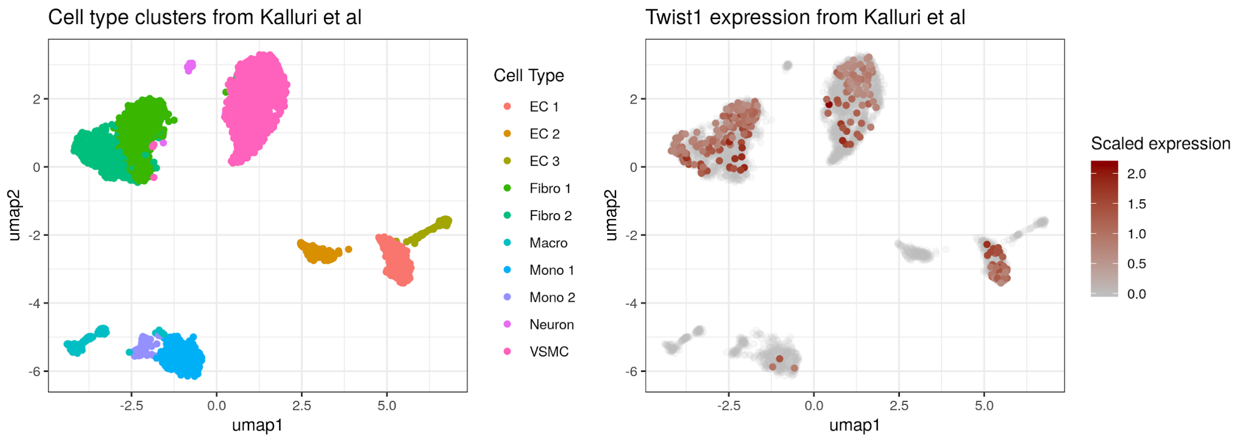

Figure S21: TWIST1 transcript expression in aortic single cell data

Expression of *Twist1* in mouse aorta cell subtypes, with clusters as identified in (4). EC1 corresponds to a subset of endothelial cells characterized by expression of genes involved in extracellular matrix organization. This corresponds to a cluster identified in (5) as having a mesenchymal phenotype, quiescence, and high mitochondrial content, preceding the others two more differentiated states in pseudotime. (see Methods Section Genetic Colocalization of AAC with other Phenotypes)

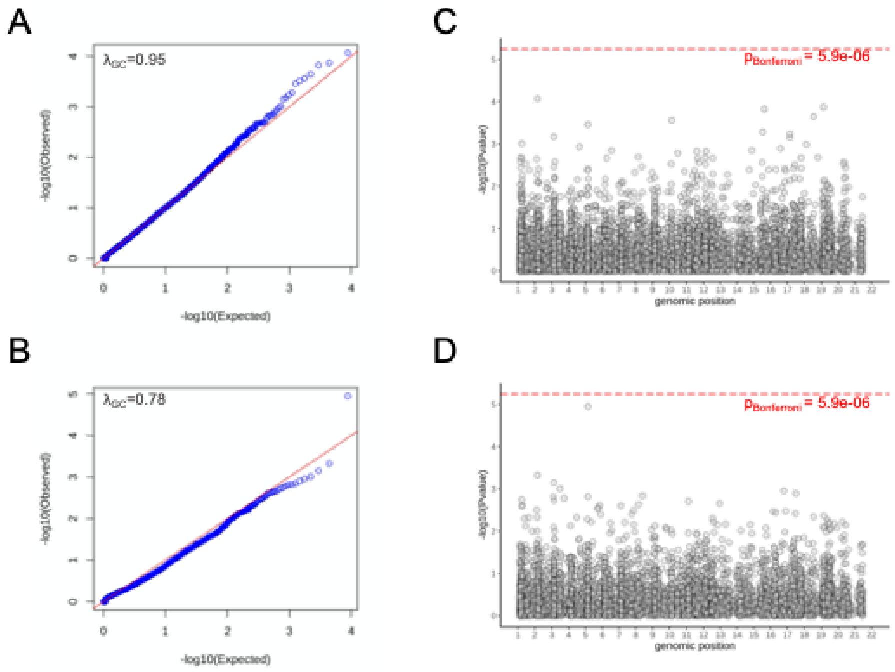

Figure S22: Rare variant analysis

Rare variant association study (RVAS) of abdominal aortic calcification (AAC) in the UK Biobank. (A) Q-Q plot demonstrates calibration of optimized test statistics combining burden test and SKAT, as implemented in SAIGE-GENE, for a model in which the outcome variable is rank normalized AAC values for n=11,749 samples of European ancestry with both AAC quantification and exome sequence data. Predicted loss-of-function variants were grouped by gene. For details on the regression modelling, see Supplementary Methods. (B) Q-Q plot depicting association study with dichotomized outcomes. In the dichotomous RVAS, cases were defined by raw AAC score >=3 and controls were defined by raw AAC score <3. There were 1,274 cases and 10,475 controls (see Methods Section RVAS). (C) Manhattan plot of association study from the continuous RVAS. (D) Manhattan plot of the association study from the dichotomous RVAS. There were no associations below Bonferroni significance threshold in either study.

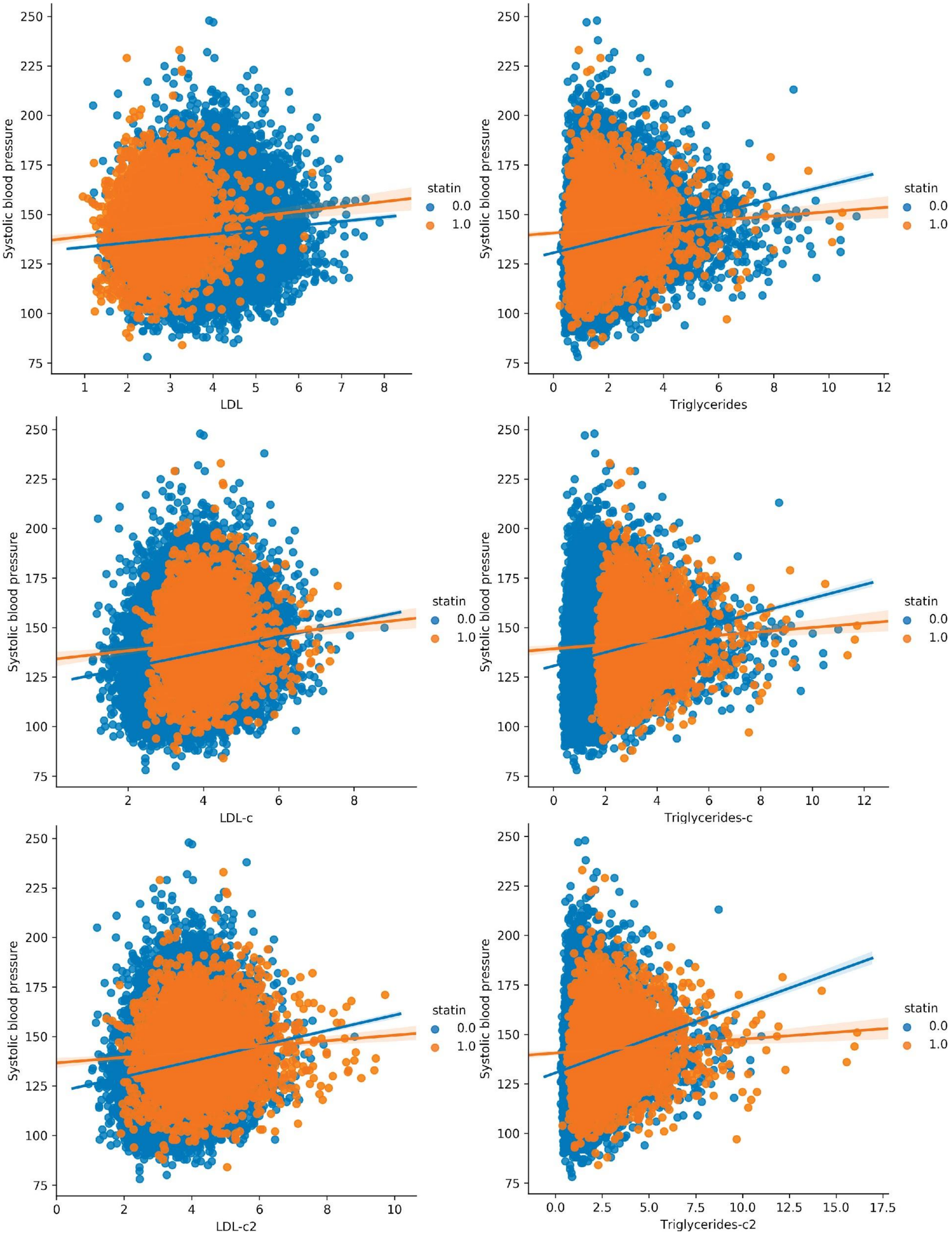
Systolic blood pressure
LDL
Triglycerides
LDL-c
Triglycerides-c
LDL-c2
Triglycerides-c2
statin
0.0
1.0
250
225
200
175
150
125
100
75
1 2 3 4 5 6 7 8
0 2 4 6 8 10 12
2 4 6 8
0 2 4 6 8 10
0.0 2.5 5.0 7.5 10.0 12.5 15.0 17.5

Figure S23: Effect of statins on LDL-Blood Pressure relationship.

The relationship between baseline LDL and Triglyceride levels to blood pressure is plotted for 38,264 UK biobank participants before and after statin correction (c1 and c2 represent the two statin correction methods). Blue represents people who don't take statin medication are shown in blue while statin takers are shown in orange. The uncorrected LDL levels are lower than that predicted by blood pressure for statin takers and the correction methods reduce this discrepancy.

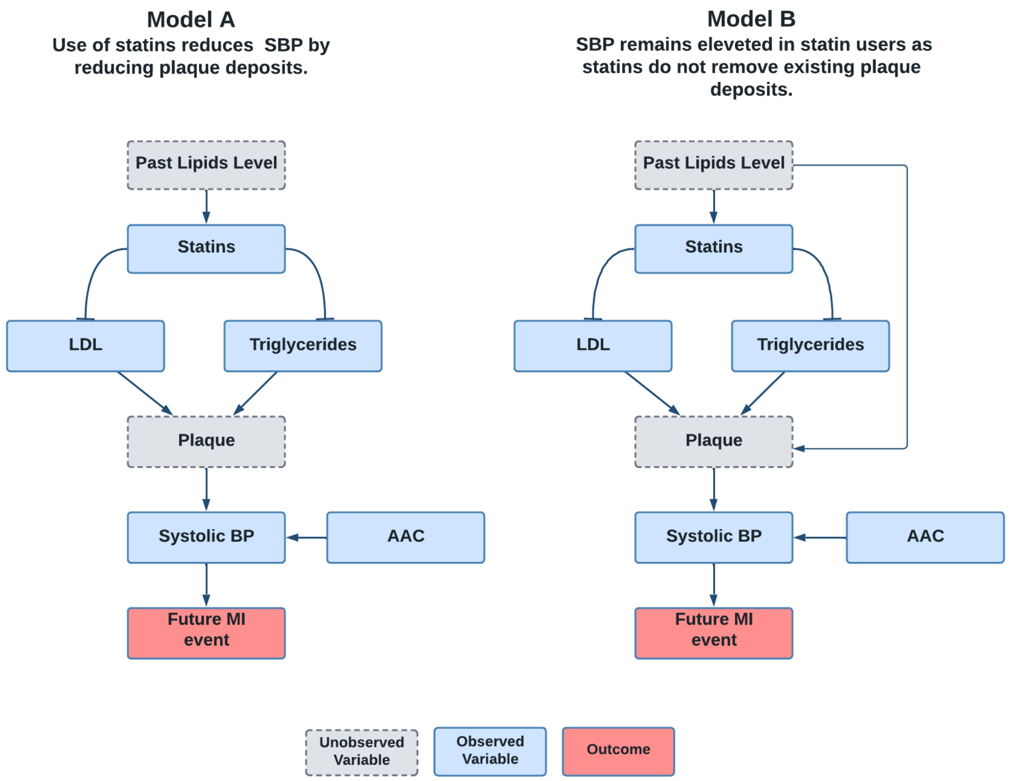

Figure S24: Myocardial infarction model of LDL, statins, and aortic calcification.

Graphical models for possible mechanism of association between use of statins, lipid levels, AAC, elevated SBP, and future MI events.

1. AAC forms an independent pathway for increased MI (AAC → reduced elasticity of arteries → higher SBP → increased MI)
2. LDL and triglyceride levels affect plaque formation → higher SBP → increased MI.
3. Statins prescribed based on previous diagnosis of elevated LDL and triglyceride levels reduce formation of plaque deposits through LDL and Trig reduction.
    a. Model# A: Statins do not affect acute MI events as they only affect SBP through lipid levels.
    b. Model#B: In **Figure S25,** we show that users of statin have higher SBP than would be predicted by lipid levels alone. This is likely because old plaque deposits are not cleared by statins and continue to affect blood pressure.

Hence, we apply statin correction to lipid levels to get an unbiased estimate of LDL and Trig risk and compare it to AAC.

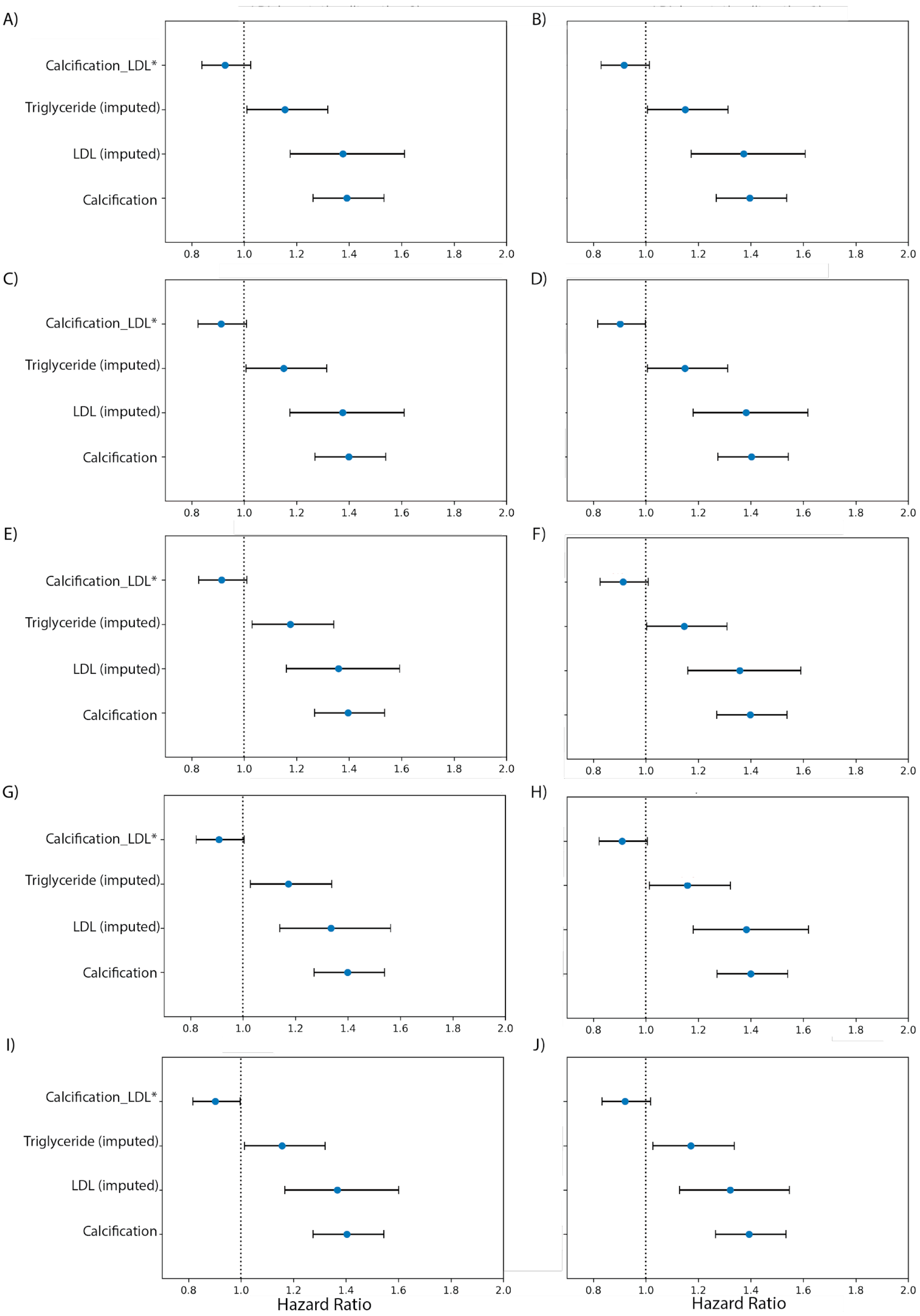

A)
B)
C)
D)
E)
F)
G)
H)
I)
J)
Calcification_LDL*
Triglyceride (imputed)
LDL (imputed)
Calcification
0.8
1.0
1.2
1.4
1.6
1.8
2.0
Hazard Ratio

Figure S25: Consistency of LDL imputation procedure for comparing LDL risk to Calcification risk.

Ten random initializations with MICE were used to impute the LDL level for statin users while LDL levels for non-statin users were used unadjusted. Cox proportional hazards were used to compare the hazard from LDL and aortic calcification over the UK biobank population. In spite of the variability from imputation, the hazard ratios for LDL and calcification risk levels were similar across all 10 models (see Methods Section Prognostic Analyses of Aortic Calcification).

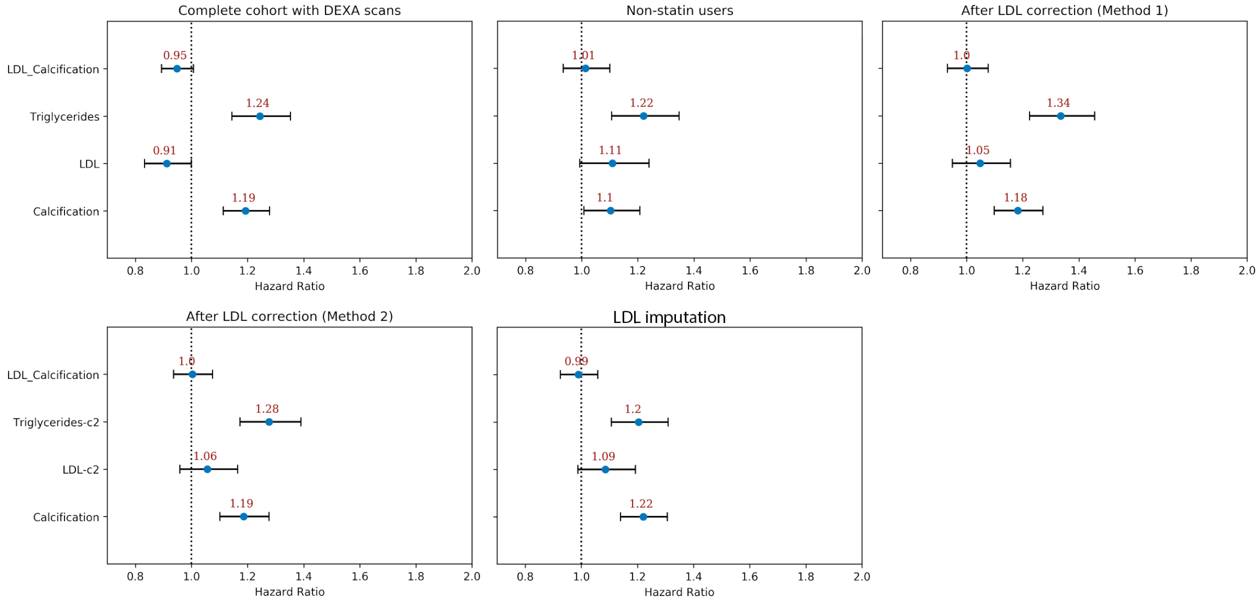

Figure S26: CoxPH association of AAC and LDL for CVD events post imaging visit.

The CVD events represent composite outcomes from MI, angina pectoris, heart failure, ischemic heart disease, and occlusion and stenosis of precerebral arteries. We compared the hazard ratios for AAC, LDL, and triglycerides using a multivariate CoxPH model with age and sex covariates. (A) Whole cohort - no statin adjustment. (B) Subset of only non-statin users. (C) Whole cohort after adjusting for statin using method 1 - assumes that LDL levels are reduced by 1.25 mmol/L in statin users. (D) Whole cohort method 2 - assumes that LDL levels are reduced by 35% in statin users. (E) Whole cohort imputation of LDL levels based on remaining biomarkers and physiological measurements (Methods). The blue dots represent mean hazard ratio, while error bars represent 95% confidence intervals. Interaction term between LDL and calcification is starred. This analysis was done with 2.9 years of median follow up time post imaging visit. LDL was measured during the baseline visit (see Methods Section Prognostic Analyses of Aortic Calcification).

Supplementary Methods

Machine Learning Methods:

We developed an ensemble machine learning approach that combined two pipelines to score aortic calcification levels based only on lumbar spine DEXA scans. Both models developed as part of this work estimate AAC scores via the following 3 steps:

1. Segmentation of the lower spine region.
2. Localization of the Aortic region using a spine-curve fitting method.
3. Regression on the localized region to predict the calcification levels from the aortic region.

The DEXA images analyzed in this manuscript were downloaded in three batches. The DEXA images were not all identical in size, but were similar in scale: 444.7(mean) +/- 27.4(SD) pixels wide and 940.0(mean) +/- 8.3(SD) pixels high. The second batch of images that we analyzed had a different distribution of dimensions: 755.1(mean) +/- 65.3(SD) pixels wide and 1665.9(mean) +/- 114.7(SD) pixels high. The third batch of images was similar to the second: 781.5(mean) +/- 43.9(SD) pixels wide and 1654.8(mean) +/- 104.5(SD) pixels high. All images in the second and third batches were rescaled to 55% of their original size along each axis.

Machine Learning Pipeline#1

Detection of the Vertebrae Using Segmentation

Previous studies (6–8) have successfully used a fully convolutional neural network to segment out the vertebrae and determine the spinal curvature. The segmentation model in Pipeline 1 employs a similar approach using a U-Net to achieve semantic segmentation of the Pelvis and the lower spine.

In order to localize the region of the DEXA scan corresponding to the Aorta, correct anatomic locations of the Pelvis (**P**) and 4 vertebrae (**L3-L5**, **S1**) are needed. The network to achieve the necessary segmentation is based on a U-Net architecture. The network architecture consists of a contracting path (left side) and an expansive path (right side) as shown in **Figure S4**. The contracting path follows the typical architecture of a convolutional network. It consists of the repeated application of two 3x3 convolutions (unpadded convolutions), each followed by a batch normalization layer, a rectified linear unit (ReLU) and a 2x2 max pooling operation with stride 2 for downsampling. Each downsampling step doubles the number of feature channels. Every step in the expansive path consists of an upsampling of the feature map followed by a 2x2 convolution ("up-convolution") that halves the number of feature channels, a concatenation with the corresponding feature map from the contracting path (residual connection), and two 3x3 convolutions, each followed by a ReLU. At the final layer a 1x1 convolution is used to map each 16-component feature vector to one of 3 classes - spine, pelvis or background.

The UKBB dataset has a total of 31,494 lumbar spine DEXA scans of patients available to score. Of those, 200 images were randomly chosen for manual annotations of **P** and **L3-L5, S1**. The png images of the scans were loaded on an open sourced annotation tool, QuPath (9), and the relevant anatomical locations were marked using the polygon tool. The user annotations were converted to binary masks for the 3 classes - Pelvis, vertebrae and background . Of the 200 images, 175 were used for training and cross-validation while the rest were reserved as an unseen test set to quantify the segmentation performance.

Simple data augmentations in the form of crop and zoom, left-right flips and slight rotations (upto +/- 10 degrees) were used to augment the images in the training dataset. The network was trained with the multi-class cross-entropy loss using an Adam optimizer. The evaluation metric is the mean IoU for each of the 3 classes which is defined as (**Figure S5**):

$$IoU = \frac{true-positives}{true-positives+false-negatives+false-positives}$$

Aortic Region Extraction

Once the segmentations of **P** and **L3-L5, S1** are completed, the aortic region is localized and extracted via the following steps:

1. Run the segmentations through post-processing steps to:
   a. Eliminate false positives for **P** (determined through co-localization with **L3-L5, S1**).
   b. Fix broken or missing vertebrae using median height and width estimates for the different vertebrae
2. Determine the centroids of each of **L3, L4, L5, S1** and **P**. Fit a spline curve to pass through each of these points to determine the spinal curvature
3. Along the spinal curvature, move to the right by a fixed offset $\mathbf{A_{off}}$ and extract a rectangular region whose width is $\mathbf{A_{width}}$ and whose height is determined by the vertical distance between the centroids of **L3** and **P**. Crop and/or pad the images to be of size 196x196 pixels.

As illustrated in **Figure S6**, at the output of this step, the Aortic regions will be localized and extracted from the original CT images. These regions are then passed on to the Regression module to be converted into a calcification score.

Regression

The final step in translating the DEXA images to a calcification score is to run a regression model that maps the extracted aortic regions to a score. The regression model consists of a backbone feature extractor followed by a fully connected layer and an output layer with a single node that outputs the calcification score. The backbone network is typically borrowed from the classification task on the ImageNet database. This paper explores 3 different backbone feature extractors:

1. InceptionV3 (10)
2. ResNet50 (11)
3. Custom convolutional neural network consisting of 4 layers of convolution, batch normalization and a ReLu activation layer each.

Of the 1300 user annotated calcification levels, 1000 images were used for training and cross-validation while the other 300 were held out as an unseen test set. As illustrated in **Figure S7**, the ground truth data is highly skewed towards the lower calcification scores creating a high degree of data imbalance. In order to introduce some degree of balance, the dataset was augmented in a stratified manner. Augmentation routines include horizontal and vertical flips, random rotations, zoom and crop, random brightness changes, random contrast changes as well as random hue changes. During agile augmentation, images with higher scores were augmented with different combinations of the routines to produce as many as 32 variations of every image while images with lower calcification scores were augmented only once or not at all.

Since the training set was quite small, both InceptionV3 and ResNet backbones overfit very readily to the dataset and performed quite poorly on the validation sets. The best results on the validation set were obtained when using the custom convolutional network as the backbone feature extractor. All results presented in this paper were, therefore, generated using this custom backbone network shown in the figure.

During training, the regression model used a weighted mean squared error metric wherein errors in the higher calcification scores were weighted higher than those in the lower scores, once again with the intention of offsetting the high degree of skew in the ground truth score distributions. The network is trained with an Adam optimizer and the evaluation metric is the correlation between the predicted scores and the median user scores. The final correlation score between the manually annotated AAC scores and the predicted AAC scores on an unseen validation set is shown in **Figure S7**.

Machine Learning Pipeline#2

The second pipeline we developed in this manuscript seeks to score the degree of abdominal aortic calcification through analysis of a DEXA image. The analysis occurs in three steps:

1. identification of the spine, and use of the labelled vertebrae to identify & extract the small region within the image that will contain the aorta;
2. classification of the aortic sub-image using three models (one classifying calcified-vs-not using a low threshold for calcification, another classifying the same using a high threshold for calcification, and a third classifying the extent of background noise in the image into low, medium, or high categories);
3. use of the scores assigned to each category by the above classification models to assign a calcification score using an ML regression model.

Detection of the Vertebrae Using Segmentation

The clinical scoring system for AAC involves the assignment of points based on the intensity with which the walls of the abdominal aorta can be visualized in an x-ray image adjacent to the L1-L4 lumbar vertebrae. Here, we attempted to isolate the relevant portion of the DEXA image for such evaluation by first mapping the spine and then capturing a smaller image adjacent to the L3 & L4 vertebrae. For our study, DEXA images were available in bulk. But they are not ideally suited to the detection of AAC: high levels of background intensity would often appear adjacent to the L1 & L2 vertebrae, often due to visualization of the rib cage. We therefore sought to isolate just the part of the image adjacent to vertebrae L3 & L4. That strategy is illustrated by the examples in Figure S8, with the lumbar vertebrae labelled in green and the desired aortic region circled in orange. This goal was achieved through two sequential processes as described below:

The foundation of this AAC scoring system is the annotation of the spine and its constituent vertebrae in the DEXA image. That annotation defines the landmarks that are used to isolate the portion of the image that contains the aorta, a task that cannot be performed independently of these landmarks because the aorta is only clearly visible in these images when it is extensively calcified.

This section describes the steps taken to annotate as many individual vertebrae as possible while minimizing the false-positive annotation of other parts of the image as vertebrae. This task was initially approached with a simple object-detection model. In order to correct mistakes made during the initial segmentation step, we chose to implement a series of analyses & models to detect & correct errors. The end product is an analysis pipeline that is summarized in **Figure S8**.

Initially, vertebrae are boxed using an object-detection model that is applied with a very low score threshold ("draw vertebra boxes"; few false negatives, many false positives). Incorrect annotations that are far from the spine are easily detected and removed using the unusual vertebra-to-vertebra angles that are produced ("trim off-axis vertebrae"). Each remaining vertebra is then scrutinized by a classification model that is trained using high-quality versus low-quality annotations that were produced by the initial object-detection model, i.e. a model to specifically address the first model's errors ("remove low-quality vertebrae"). Then, missing vertebrae (either missed initially or removed because they were poorly defined) are detected and filled in, using the context of the nearby annotations to increase sensitivity and specificity versus the initial object-detection model. In the first case, skipped vertebrae are detected ("classify vertebra pairs") and filled in by a model appropriate to the number of consecutive vertebrae that were missed ("fill-in one" or "fill-in multi"). In the second case, missing vertebrae at the bottom of the spine are detected ("have we hit bottom?") and filled in ("extend down by one"). The performance of each substep is summarized in **Figure S9**.

Aortic Region Extraction

After identifying all the vertebrae in the spine, the region of the scan adjacent to the L3 and L4 lumbar vertebrae was extracted: this was the region of the scan with the abdominal aorta. Given an outline of the spine, it is straightforward to determine which vertebra is which given a single reference point. In these DEXA images, the border between the L4 and L5 vertebrae was often coincident with the top edge of the spine. An object-detection model was trained by transfer learning from the ssd_mobilnet_v1 model**.** The best box returned by the model was used, irrespective of its score. Performance was measured using IoU (0.893 and 0.818 for training and test sets respectively).

The aortic images were created by stacking rectangular images gathered from adjacent to the L3 and L4 vertebrae, using the following four-step process:

a. definition of a vector pointing towards the aorta for each vertebra;
b. definition offset distances and image widths using the dimensions of the lumbar bounding boxes and aortic vectors;
c. definition of sub-image heights & extraction of the aortic sub-images adjacent to L3 and L4; and
d. rightening & stacking the L3- and L4-adjacent sub-images into a single output image.

For each input image (full DEXA scans), the end result of the analyses described in the links above was a smaller image depicting the regions adjacent to the L3 vertebra and the L4 vertebra. The inability to identify both of those vertebrae resulted in no output aortic image.

Regression

The overall distribution of AAC values is highly skewed towards little or no aortic calcification. This property was observable in the training set, with most rater-generated scores at zero (Figure 1). We developed two models, each described below, in order to address the problem of sparsity of training data for high-calcification scores. The first model focuses on the lower end of the distribution, and was trained to distinguish between images with zero-value versus non-zero AAC scores. The second focuses on the higher end of the score distribution, where I used a more-efficient but less-precise-than-scoring method to enrich a larger test data set for high-calcification images.

a) **Model 1:** For the training data, four raters scored calcification as an integer, and the median value was taken of those four scores. For this low-threshold AAC model, any image with a rater-median score of 0.5 or greater was considered "calcified". That approximately split the training set (264 "calcified" images, 332 "non-calcified" images). The ROC curves to measure the accuracy of this model is shown in **Figure S10** while Cohen's kappa for these models were 0.58 and 0.33 for the training and test sets respectively.
b) **Model 2:** For the purpose of helping to develop higher-threshold calcification models, we designated a set of 5000 "sandbox" images that were non-overlapping with the validation and training sets, and were therefore of potential use to the model developers as training data, but for which they would not provide manual ratings. We used those data by iteratively applying a low-threshold model to those images, sorting out the "calcified" images, then manually enriching those images for yet-higher calcification values by selecting the apparently-more-calcified images from image pairs until we had sufficient data to train another model. We repeated this process until we had arrived at a training set with 170 "calcified" and 4654 "non-calcified" images, at a threshold that we estimated to be at approximately score=5.
   For evaluation, the original training and test sets suffered from sparsity of high-scoring data, making our evaluation of their performance sensitive to statistical noise. The ROC curves and Cohen's kappa values for a) the actual, "sandbox"-enriched training set (yellow - 0.71); b) the original training set, with a score threshold of 5 (green - 0.73), and c) the original test set, again with a score threshold of 5 (blue - 0.53) (**Figure S10**).

For the final output value of the model (AAC score), we built and trained a small regression model to input the probability scores from the classification models above and output an AAC estimate. For the binary classification tasks, we used just one of the two outputs. The structure of the model is shown in **Figure S11**. This model has 52 trainable parameters. We experimented with many model structures and multiple attempts at training the model described above, evaluating performance using the "test" set. The statistics for the final model are shown for an unseen validation set in **Figure S12**.

Ensemble Model

Due to the paucity of labeled data and to avoid overfitting by using the same labeled data for training both pipelines and the ensemble model, we used an unsupervised ensemble method to combine the scores from both pipelines. In particular, we took the mean calcification levels predicted by both pipelines as the ensemble prediction. We tested the accuracy of the ensemble method on 300 test images that were not used for training either model and the accuracy of the ensemble model was higher than the accuracy of either pipeline (Table S3). Hence, we used the ensemble method to quantify calcification for all participants within the cohort and these scores were used for all downstream analysis in this article.

Biomarker Analysis Methods

We used linear regression to examine the association of aortic calcification with various biomarkers and physiological markers. For these models, univariate associations of aortic calcification with each marker were evaluated with two different models. In model 1, all univariate associations were performed after adjusting for age and sex, while in model 2, we also adjusted for BMI, Townsend deprivation index, smoking status, and race in addition to age and sex. We chose these factors based on their associations with traditional cardiovascular outcomes and/or their association with aortic calcification. All p-values were calculated using the two-tailed t-statistic of the estimated association. The associations were considered to be significant after multiple hypothesis testing (i.e., Bonferroni Correction with p-value < 1.2e-4). These estimations were calculated using the statsmodels package v.0.9.0 in python (12).

Survival Analysis Methods

Comparison of risk from Aortic Calcification and LDL

We used Cox proportional hazards models (13) to compare the risk for acute myocardial infarction from aortic calcification and LDL with the following model:

$$\lambda(t) = \lambda_0(t)\, e^{\alpha}$$

where:

$$\alpha = \alpha_0 + \alpha_{LDL} \times LDL + \alpha_{triglyceride} \times triglyceride + \alpha_{AAC} \times AAC + \alpha_{LDL.AAC} \times LDL \times AAC$$

where α_0 represents the baseline risk, α_{LDL} represents the risk due to increase of LDL, α_{AAC} represents the risk due to increase in aortic calcification, $\alpha_{triglyceride}$ represents the risk due to increase in triglycerides, and $\alpha_{LDL.AAC}$ was used to measure the risk due to the interaction between LDL and triglycerides. The LDL and triglyceride levels were logged and standardized while aortic calcification levels were standardized before measuring the risk for acute MI events.

Acute MI events were defined as the first occurence of I21, I22, I23, I24.1 or I25.2 from the ICD10 codes in the electronic health records similar to (14). Any participant diagnosed with acute MI events prior to baseline was removed from analysis before comparing risks from aortic calcification and LDL. We created four different models to correct the risk for statin usage that reduces LDL without reducing systolic blood pressure completely (Figure S24) in addition to a naive model in which no statin correction was performed. The first model was a naive model built for statin nonusers and the estimates for LDL risk are confounded by survivorship bias due to the nonrandomness of statin usage. In model 2, we adjusted the LDL and triglyceride levels for statin users by adding 1.25 mmol/L to the LDL levels for statin users while no adjustment was performed for non-statin users (15) (Figure S24). In model 3, we adjusted the LDL and triglyceride levels for statin users by dividing the measured LDL for statin users by 0.65 while no adjustment was performed for non-statin users (15) (Figure S24). Finally, in model 4, we imputed the LDL levels for statin users based on systolic blood pressure, diastolic blood pressure, pulse, age, sex, and measured blood biomarker (albumin, alkaline phosphatase, alanine aminotransferase, aspartate aminotransferase, bilirubin, urea, calcium, creatinine, cystatin C, gamma glutamyltransferase, glucose, glycated haemoglobin (HbA1c), insulin growth factor, phosphate, rheumatoid factor. Testosterone, sex hormone binding globin, total protein, urate, and vitamin D) levels in serum. Imputation was performed using multiple imputation by

chained equations (MICE) (16). In the MICE procedure a series of regression models are run whereby each variable with missing data is modeled conditional upon the other variables in the data. To evaluate the sensitivity of the results to the imputed values, we performed the risk of LDL and aortic calcification with ten different sets of LDL and triglyceride imputations and each MICE imputation was performed with 50 iterations to relax from initial estimates. The estimated risk scores were not sensitive to the imputation set as shown in Figure S25.

Genetics Methods

Rare variant association study (RVAS)

Exome sequencing variant calls from the raw FE variant calling pipeline ((17)) were downloaded from the UK Biobank website (http://biobank.ctsu.ox.ac.uk/crystal/field.cgi?id=23160). QC was performed in PLINK v.1.90 using the following criteria: removal of samples with discordant sex (no self-reported sex provided, ambiguous genetic sex, or discordance between genetic and self-reported sex), sample-level missingness <0.02, European genetic ancestry as defined by the UK Biobank ((18) Variant annotation was performed using VEP v100, filtered for rare (MAF<0.01) putative loss-of-function variants including predicted high-confidence loss-of-function variants, predicted using the LOFTEE plugin ((19)). 11,749 samples and 18,102 genes were analyzed in a generalized linear mixed model as implemented in SAIGE-GENE (Zhou et al, 2020). For the dichotomous study, AAC was binarized into 1,274 cases, defined as raw AAC score >=3, and 10,475 controls, defined as raw AAC score < 3. Rank-normalized AAC and binarized AAC were both regressed on gene carrier status, adjusted for genetic sex, age, and PC1:10 as fixed effects and genetic relatedness as a random effects term. A kinship matrix was built in SAIGE off of a filtered set of genotyped variants (r2<0.2, MAF>=0.05, HWE p>1e-10 in European population). A filtering step of at least 10 loss-of-function carriers per gene was applied, resulting in 8,794 genes.

Common variant genome wide association study (GWAS)

We used the UKBB imputed genotypes (18), excluding SNPs with a minor allele frequency <1% and poor imputation quality (info value <0.9). We removed particpants who were not Caucasian, exhibited sex chromosome aneuploidy, heterozygosity outliers, or genotype call rate outliers. In total, we considered 9,572,557 SNPs and 31,786 individuals (Supplementary Table 1) for genetic analysis.

To conduct the genetic association study, we used BOLT-LMM (20) and standardized machine-learned AAC, including genotype SNP chip (Illumina vs Affimetrix), sex, age, age^2, and recruitment center as fixed effect covariates and genetic relatedness derived from genotyped SNPs as a random effect to control for population structure and relatedness. We verified that the test statistics showed no inflation compared to the expectation using the genomic control lambda coefficient (1.056) and the intercept (1.024, s.d. 0.0086) of linkage disequilibrium (LD) score regression (LDSC) (21).

GWAS Meta-analysis

We combined the summary statistics from our UK Biobank study with those from the CHARGE consortium (22) using a fixed effects metaanalysis as implemented in the software METAL (23). As effect sizes were not comparable across studies, and not available for the CHARGE dataset, we used a sample-size weighting scheme to combine the studies. The total sample size was 41,203. For ease of interpretation, we included only SNPs present in all the

CHARGE sub-studies and the UK Biobank analysis for downstream analysis. This resulted in a total of 6,360,639 SNPs.

We estimate the effect size and standard error on a standardized scale using the formulae
$\beta = \frac{z}{\sqrt{2p(1-p)(n+z^2)}}$ and $\sigma = \frac{1}{\sqrt{2p(1-p)(n+z^2)}}$
where p is the minor allele frequency, n is the combined sample size, and z the z-statistic (24).

Genetic architecture of AAC

Identification of distinct association signals

We performed approximate conditional analysis using GCTA (25), considering all variants that passed quality control measures and were within 500kb of the locus index variant. As a reference panel for LD calculations, we used genotypes from 5,000 UKBB participants (18) that were randomly selected after filtering for unrelated, Caucasian participants. We excluded the major histocompatibility complex (MHC) region due to the complexity of LD structure at this locus (GRCh37::6:28,477,797-33,448,354; see https://www.ncbi.nlm.nih.gov/grc/human/regions/MHC). For each locus, we considered variants with locus-wide evidence of association ($\text{p-value}_{joint}<10^{-6}$) to be conditionally independent.

Construction of genetic credible sets

For each distinct signal, we calculated credible sets (26) with 95% probability of containing at least one variant with a true effect size not equal to zero. We first computed the natural log approximate Bayes factor (27), Λ_j , for the j th variant within the fine-mapping region:

$$\Lambda_j = ln\left(\sqrt{\frac{V_j}{V_{j+\omega}}}\right)\frac{\omega\beta^2}{2V_j(V_j+\omega)}$$

where β_j and V_j denote the estimated allelic effect (log odds ratio for case control studies) and corresponding variance. The parameter ω denotes the prior variance in allelic effects and is set to $(0.2)^2$ for case control studies (27) and $(0.15\sigma)^2$ for quantitative traits (28), where σ is the standard deviation of the phenotype estimated using the variance of coefficients ($Var(\beta_j)$), minor allele frequency (f_j), and sample size (n_j; see the sdY.est function from the coloc R package (28)):

$$2n_j f_j(1 - f_j) \sim \sigma^2 \frac{1}{Var(\beta_j)} - 1$$

Here, σ^2 is the coefficient of the regression, estimating σ such that $\sigma = \sqrt{\sigma^2}$.

We calculated the posterior probability, π_j, that the j th variant is driving the association, given l variants in the region, by:

$$\pi_j = \frac{(1-\gamma)\Lambda_j}{l\sum_{k=0}^{l}\Lambda_k}$$

where γ denotes the prior probability for no association at this locus and k indexes the variants in the region (with k=0 allowing for the possibility of no association in the region). We set γ=0.05 to control for the expected false discovery rate of 5%, since we used a threshold of $p\text{-value}_{marginal}<5x10^{-8}$ to identify loci for fine-mapping. We note that setting γ=0 generates credible sets as proposed by The Wellcome Trust Case Control Consortium et al. (26) and is suitable when one is very confident of the identified loci (e.g., replicated across many studies).

To construct the credible set, we (i) sorted variants by increasing Bayes factors (natural log scale), (ii) included variants until the cumulative sum of the posterior probabilities was >=1−c, where c corresponds to the credible set cutoff of 0.95.

Heritability estimates

We estimated the heritability of each trait using the restricted maximum likelihood method (29), as implemented in BOLT-LMM with the --reml option.

Genetic correlations

We estimated the genetic correlation of AAC with phenotypes using an LDSC-based method (30), as implemented in the LD Hub web resource (31). For this analysis and all other analyses using LDSC, we followed the recommendation of the developers and (i) removed variants with imputation quality (info) <0.9 because the info value is correlated with the LD score and could introduce bias, (ii) excluded the major histocompatibility complex (MHC) region due to the complexity of LD structure at this locus (GRCh37::6:28,477,797-33,448,354; see https://www.ncbi.nlm.nih.gov/grc/human/regions/MHC), and (ii) restricted to HapMap3 SNPs (32). We restricted our analysis to the 836 traits analysed in European populations. We successfully estimated genetic correlation for 754 traits. We additionally analysed 31 blood and urine biomarkers using data from the UK Biobank, to give a total of 785 traits.

Partitioning of AAC heritability

We used LDSC to partition the heritability of AAC according to functional categories (33) as well as tissue/cell type specific annotations (34).

For functional categories, we used the baseline v2.2 annotations provided by the developers (https://data.broadinstitute.org/alkesgroup/LDSCORE). Following Finucane et al. (34), we calculated tissue specific enrichments using a model that includes the full baseline annotations as well as annotations derived from (i) chromatin information from the NIH Roadmap Epigenomics (35) and ENCODE (36) projects (including the EN-TEx data subset of ENCODE which matches many of the GTEx tissues, but from different donors), (ii) tissue/cell type specific expression markers from GTEx v6p (37) and other datasets (38,39), and (iii) immune cell type expression markers from the ImmGen Consortium (40). For each annotation set, we controlled for the number of tests using the Storey and Tibshirani procedure (41). As noted by (33), although heritability is non-negative, the unbiased LDSC heritability estimate is unbounded; thus, it is possible for the estimated heritability, and therefore enrichment, to be negative (e.g., if the true heritability is near zero and/or the sampling error is large due to small sample sizes).

In order to enable visualization, we grouped tissue/cell types into systems (e.g., "blood or immune", "central nervous system"). These groupings and labels were the same as those used in Finucane et al. (34), except for (i) the immune expression labels which we extended to include "stem cells" and "stromal cells" according to the ImmGen cell type classifications (http://www.immgen.org) and (ii) the "pancreas" label which we replaced with an "endocrine" label composed of the following tissue/cell types: adrenal gland, ovary, pancreas, pituitary, prostate, testis, thyroid,

adrenal cortex, adrenal gland, endocrine gland, gonads, granulosa cells, islets of langerhans, and glucagon sensing cells.

Genetic colocalization of AAC with other phenotypes

We performed colocalization analysis using the coloc R package (28) using default priors and all variants within 500kb of the index variant. As performed by Guo et al. (42), we considered two genetic signals to have strong evidence of colocalization if PP3+PP4≥0.99 and PP4/PP3≥5 and suggestive evidence of colocalization if PP3+PP4≥0.8 and PP4/PP3≥3. For gene expression colocalizations, we used summary statistics from GTEx v7 (37). For disease and quantitative trait colocalizations, we used UKBB summary statistics of PheCodes (43), normalized quantitative traits (http://www.nealelab.is/blog/2017/7/19/rapid-gwas-of-thousands-of-phenotypes-for-337000-samples-in-the-uk-biobank). For analysis we selected UKBB phenotypes where the minimum p-value within the +-500kb region around the locus tag SNP was <$5x10^{-8}$.

Functional Analysis of GWAS Hits

At the chromosome 7 locus, we identified two independent signals. For the primary association the lead SNP, rs2107595 (p-value=$1.47x10^{-20}$) was the only SNP in the 95% credible set. There were 3 SNPs in the 95% credible set of the secondary association (Supplementary Table S8). rs2107595 lies in a non-coding region between HDAC9 and TWIST1. This locus has been linked to several cardiovascular traits including ischaemic stroke(44,45) in large and small vessels(46,47), coronary artery disease (CAD)(46), peripheral artery disease (48,49), blood pressure(48,50–52)pulse pressure(3), and MoyaMoya disease(53). This signal colocalizes with the expression of TWIST1 in the aortic artery, but not the expression of HDAC9 in any of the tissues considered (Figure S21). In addition, we found TWIST1 expression was associated with tibial artery calcification (beta=0.265, SE=0.0649, p-valueBonferroni=0.0002616), but not HDAC9 in any tissue considered (minimum p-valueBonferroni=0.2488), in histological tissues.

The second strongest association lay on chromosome 13 (lead SNP rs9510086, p-value=7.38e-10). The closest coding gene, FGF9, lies 600kb from the lead SNP. This signal colocalizes with FGF9 expression in the tibial artery (Figure S21; Supplementary Table S11). In a recent study of ascending aorta area(54), FGF9 expression has been shown to be increased in aneurysm compared to control tissue(55). This raises the possibility of a connection between vascular calcification and aneurysm.

We found an association intronic to NAV1 (lead SNP rs560804, p-value=$9.30x10^{-9}$). This signal colocalizes with NAV1 expression in heart atrial appendage, with SHISA4 in pancreas (Figure S21; Supplementary Table S11), and diastolic blood pressure (Supplementary Figure S22; Supplementary Table S12). A recent study (56) identified NAV1 as a candidate gene for aortic valve stenosis.

At the APOE rs41290120 locus (p-value=$2.9x10^{-9}$), we found no evidence for colocalization with the expression of any gene in any tissue; however, we did find strong evidence of colocalization with traits related to heart disease, red blood cell measurements, and dietary traits (Supplementary Table S12). The lead SNP at this locus, rs1065853, is in LD (r^2>0.99) with the APOE e2 allele, rs7412(57). In light of this association, the association with AAC is less clear and warrants further investigation in future studies

Follow up analysis at the rs2107595 locus

To assess the correlation between TWIST1 and HDAC9 expression and calcification visible on histological imaging, we downloaded the sample annotations from https://storage.googleapis.com/gtex_analysis_v7/annotations/GTEx_v7_Annotations_SampleAttributesDS.txt. We defined a participant's vascular tissue as calcified if the annotation 'calcification' appeared in the 'Pathology categories' field. We downloaded expression data from GTEx v7 (dbGaP Accession phs000424.v7.p2). For each arterial tissue (coronary, aorta, or tibial) and gene (TWIST1 or HDAC9), we used a logistic regression model, adjusted for age and sex, to assess the association between expression levels and calcificaiton. We used the Bonferroni procedure to correct for multiple models by multiplying each p-value by 6.
We explored *Twist1* expression patterns in single cell expression data from mouse aorta (4) (Figure S23).

In addition to the colocalizations described in the main text, we tested for colocalization with lipid-related quantitative traits directly measured in UKBB, even though no genetic association (p-value$<5x10^{-8}$) was found at this locus, due to the importance of lipids for coronary artery disease (CAD)-related phenotypes. We found no evidence of colocalization (Supplementary Tables 4, 5, 6, 7), suggesting that the genetic effect at the rs2107595 locus on AAC and CAD-related phenotypes is not directly related to lipid biology. We also repeated the colocalization analysis for CAD and blood pressure traits using genetic studies that did not include UKBB participants (2), (3). We found similarly strong evidence of colocalization for both traits (Supplementary Table 7).

Finally, given the strong colocalization of AAC with CAD and SBP signals as well as the substantially larger sample sizes of these traits ($n_{effective}$ for CAD=76,054 and SBP=340,159), we performed fine-mapping at this locus using associations with CAD and SBP in UKBB. Two SNPs, rs2107595 and rs57301765, that are in strong LD with each other (1000GENOMES:phase_3:GBR $r^2>0.99$) constituted the 95% credible sets for both traits.

References

1. Grubb A, Horio M, Hansson L-O, Björk J, Nyman U, Flodin M, Larsson A, Bökenkamp A, Yasuda Y, Blufpand H, et al. Generation of a new cystatin C-based estimating equation for glomerular filtration rate by use of 7 assays standardized to the international calibrator. *Clin Chem* (2014) 60:974–986.

2. Nikpay M, Goel A, Won H-H, Hall LM, Willenborg C, Kanoni S, Saleheen D, Kyriakou T, Nelson CP, Hopewell JC, et al. A comprehensive 1,000 Genomes-based genome-wide association meta-analysis of coronary artery disease. *Nat Genet* (2015) 47:1121–1130.

3. Hoffmann TJ, Ehret GB, Nandakumar P, Ranatunga D, Schaefer C, Kwok P-Y, Iribarren C, Chakravarti A, Risch N. Genome-wide association analyses using electronic health records identify new loci influencing blood pressure variation. *Nat Genet* (2017) 49:54–64.

4. Kalluri AS, Vellarikkal SK, Edelman ER, Nguyen L, Subramanian A, Ellinor PT, Regev A, Kathiresan S, Gupta RM. Single-Cell Analysis of the Normal Mouse Aorta Reveals Functionally Distinct Endothelial Cell Populations. *Circulation* (2019) 140:147–163.

5. Lukowski SW, Patel J, Andersen SB, Sim S-L, Wong HY, Tay J, Winkler I, Powell JE, Khosrotehrani K. Single-Cell Transcriptional Profiling of Aortic Endothelium Identifies a Hierarchy from Endovascular Progenitors to Differentiated Cells. *Cell Rep* (2019) 27:2748–2758.e3.

6. Han Z, Wei B, Mercado A, Leung S, Li S. Spine-GAN: Semantic segmentation of multiple spinal structures. *Med Image Anal* (2018) 50:23–35.

7. Fan G, Liu H, Wu Z, Li Y, Feng C, Wang D, Luo J, Wells WM 3rd, He S. Deep Learning-Based Automatic Segmentation of Lumbosacral Nerves on CT for Spinal Intervention: A Translational Study. *AJNR Am J Neuroradiol* (2019) 40:1074–1081.

8. Lessmann N, van Ginneken B, de Jong PA, Išgum I. Iterative fully convolutional neural networks for automatic vertebra segmentation and identification. *Med Image Anal* (2019) 53:142–155.

9. Bankhead P, Loughrey MB, Fernández JA, Dombrowski Y, McArt DG, Dunne PD, McQuaid S, Gray RT, Murray LJ, Coleman HG, et al. QuPath: Open source software for digital pathology image analysis. *Sci Rep* (2017) 7:16878.

10. Szegedy C, Vanhoucke V, Ioffe S, Shlens J, Wojna Z. Rethinking the Inception Architecture for Computer Vision. *arXiv [csCV]* (2015) http://arxiv.org/abs/1512.00567

11. He K, Zhang X, Ren S, Sun J. Deep Residual Learning for Image Recognition. *arXiv [csCV]* (2015) http://arxiv.org/abs/1512.03385

12. Seabold S, Perktold J. Statsmodels: Econometric and statistical modeling with python. *of the 9th Python in Science Conference* (2010)

https://www.researchgate.net/profile/Josef_Perktold/publication/264891066_Statsmodels_Econometric_and_Statistical_Modeling_with_Python/links/5667ca9308ae34c89a0261a8/Statsmodels-Econometric-and-Statistical-Modeling-with-Python.pdf

13. Cox DR. *Analysis of survival data*. Chapman and Hall/CRC (2018).

14. Millett ERC, Peters SAE, Woodward M. Sex differences in risk factors for myocardial infarction: cohort study of UK Biobank participants. *BMJ* (2018) 363:k4247.

15. Nissen SE, Tuzcu EM, Schoenhagen P, Crowe T, Sasiela WJ, Tsai J, Orazem J, Magorien RD, O'Shaughnessy C, Ganz P, et al. Statin therapy, LDL cholesterol, C-reactive protein, and coronary artery disease. *N Engl J Med* (2005) 352:29–38.

16. Azur MJ, Stuart EA, Frangakis C, Leaf PJ. Multiple imputation by chained equations: what is it and how does it work? *Int J Methods Psychiatr Res* (2011) 20:40–49.

17. Abel HJ, Larson DE, Regier AA, Chiang C, Das I, Kanchi KL, Layer RM, Neale BM, Salerno WJ, Reeves C, et al. Mapping and characterization of structural variation in 17,795 human genomes. *Nature* (2020) 583:83–89.

18. Bycroft C, Freeman C, Petkova D, Band G, Elliott LT, Sharp K, Motyer A, Vukcevic D, Delaneau O, O'Connell J, et al. The UK Biobank resource with deep phenotyping and genomic data. *Nature* (2018) 562:203–209.

19. Karczewski KJ, Francioli LC, Tiao G, Cummings BB, Alföldi J, Wang Q, Collins RL, Laricchia KM, Ganna A, Birnbaum DP, et al. Variation across 141,456 human exomes and genomes reveals the spectrum of loss-of-function intolerance across human protein-coding genes. *bioRxiv* (2019)531210. doi: 10.1101/531210

20. Loh P-R, Tucker G, Bulik-Sullivan BK, Vilhjálmsson BJ, Finucane HK, Salem RM, Chasman DI, Ridker PM, Neale BM, Berger B, et al. Efficient Bayesian mixed-model analysis increases association power in large cohorts. *Nat Genet* (2015) 47:284–290.

21. Bulik-Sullivan BK, Loh P-R, Finucane HK, Ripke S, Yang J, Schizophrenia Working Group of the Psychiatric Genomics Consortium, Patterson N, Daly MJ, Price AL, Neale BM. LD Score regression distinguishes confounding from polygenicity in genome-wide association studies. *Nat Genet* (2015) 47:291–295.

22. Malhotra R, Mauer AC, Lino Cardenas CL, Guo X, Yao J, Zhang X, Wunderer F, Smith AV, Wong Q, Pechlivanis S, et al. HDAC9 is implicated in atherosclerotic aortic calcification and affects vascular smooth muscle cell phenotype. *Nat Genet* (2019) 51:1580–1587.

23. Willer CJ, Li Y, Abecasis GR. METAL: fast and efficient meta-analysis of genomewide association scans. *Bioinformatics* (2010) 26:2190–2191.

24. Zhu Z, Zhang F, Hu H, Bakshi A, Robinson MR, Powell JE, Montgomery GW, Goddard ME, Wray NR, Visscher PM, et al. Integration of summary data from GWAS and eQTL studies predicts complex trait gene targets. *Nat Genet* (2016) 48:481–487.

25. Yang J, Ferreira T, Morris AP, Medland SE, Genetic Investigation of ANthropometric Traits

(GIANT) Consortium, DIAbetes Genetics Replication And Meta-analysis (DIAGRAM) Consortium, Madden PAF, Heath AC, Martin NG, Montgomery GW, et al. Conditional and joint multiple-SNP analysis of GWAS summary statistics identifies additional variants influencing complex traits. *Nat Genet* (2012) 44:369–75, S1–3.

26. Wellcome Trust Case Control Consortium, Maller JB, McVean G, Byrnes J, Vukcevic D, Palin K, Su Z, Howson JMM, Auton A, Myers S, et al. Bayesian refinement of association signals for 14 loci in 3 common diseases. *Nat Genet* (2012) 44:1294–1301.

27. Wakefield J. A Bayesian measure of the probability of false discovery in genetic epidemiology studies. *Am J Hum Genet* (2007) 81:208–227.

28. Giambartolomei C, Vukcevic D, Schadt EE, Franke L, Hingorani AD, Wallace C, Plagnol V. Bayesian test for colocalisation between pairs of genetic association studies using summary statistics. *PLoS Genet* (2014) 10:e1004383.

29. Yang J, Benyamin B, McEvoy BP, Gordon S, Henders AK, Nyholt DR, Madden PA, Heath AC, Martin NG, Montgomery GW, et al. Common SNPs explain a large proportion of the heritability for human height. *Nat Genet* (2010) 42:565–569.

30. Bulik-Sullivan B, Finucane HK, Anttila V, Gusev A, Day FR, Loh P-R, ReproGen Consortium, Psychiatric Genomics Consortium, Genetic Consortium for Anorexia Nervosa of the Wellcome Trust Case Control Consortium 3, Duncan L, et al. An atlas of genetic correlations across human diseases and traits. *Nat Genet* (2015) 47:1236–1241.

31. Zheng J, Erzurumluoglu AM, Elsworth BL, Kemp JP, Howe L, Haycock PC, Hemani G, Tansey K, Laurin C, Early Genetics and Lifecourse Epidemiology (EAGLE) Eczema Consortium, et al. LD Hub: a centralized database and web interface to perform LD score regression that maximizes the potential of summary level GWAS data for SNP heritability and genetic correlation analysis. *Bioinformatics* (2017) 33:272–279.

32. International HapMap 3 Consortium, Altshuler DM, Gibbs RA, Peltonen L, Altshuler DM, Gibbs RA, Peltonen L, Dermitzakis E, Schaffner SF, Yu F, et al. Integrating common and rare genetic variation in diverse human populations. *Nature* (2010) 467:52–58.

33. Finucane HK, Bulik-Sullivan B, Gusev A, Trynka G, Reshef Y, Loh P-R, Anttila V, Xu H, Zang C, Farh K, et al. Partitioning heritability by functional annotation using genome-wide association summary statistics. *Nat Genet* (2015) 47:1228–1235.

34. Finucane HK, Reshef YA, Anttila V, Slowikowski K, Gusev A, Byrnes A, Gazal S, Loh P-R, Lareau C, Shoresh N, et al. Heritability enrichment of specifically expressed genes identifies disease-relevant tissues and cell types. *Nat Genet* (2018) 50:621–629.

35. Roadmap Epigenomics Consortium, Kundaje A, Meuleman W, Ernst J, Bilenky M, Yen A, Heravi-Moussavi A, Kheradpour P, Zhang Z, Wang J, et al. Integrative analysis of 111 reference human epigenomes. *Nature* (2015) 518:317–330.

36. ENCODE Project Consortium. An integrated encyclopedia of DNA elements in the human genome. *Nature* (2012) 489:57–74.

37. GTEx Consortium, Laboratory, Data Analysis &Coordinating Center (LDACC)—Analysis Working Group, Statistical Methods groups—Analysis Working Group, Enhancing GTEx (eGTEx) groups, NIH Common Fund, NIH/NCI, NIH/NHGRI, NIH/NIMH, NIH/NIDA, Biospecimen Collection Source Site—NDRI, et al. Genetic effects on gene expression across human tissues. *Nature* (2017) 550:204–213.

38. Fehrmann RSN, Karjalainen JM, Krajewska M, Westra H-J, Maloney D, Simeonov A, Pers TH, Hirschhorn JN, Jansen RC, Schultes EA, et al. Gene expression analysis identifies global gene dosage sensitivity in cancer. *Nat Genet* (2015) 47:115–125.

39. Pers TH, Karjalainen JM, Chan Y, Westra H-J, Wood AR, Yang J, Lui JC, Vedantam S, Gustafsson S, Esko T, et al. Biological interpretation of genome-wide association studies using predicted gene functions. *Nat Commun* (2015) 6:5890.

40. Heng TSP, Painter MW, Immunological Genome Project Consortium. The Immunological Genome Project: networks of gene expression in immune cells. *Nat Immunol* (2008) 9:1091–1094.

41. Storey JD, Tibshirani R. Statistical significance for genomewide studies. *Proc Natl Acad Sci U S A* (2003) 100:9440–9445.

42. Guo H, Fortune MD, Burren OS, Schofield E, Todd JA, Wallace C. Integration of disease association and eQTL data using a Bayesian colocalisation approach highlights six candidate causal genes in immune-mediated diseases. *Hum Mol Genet* (2015) 24:3305–3313.

43. Zhou W, Nielsen JB, Fritsche LG, Dey R, Gabrielsen ME, Wolford BN, LeFaive J, VandeHaar P, Gagliano SA, Gifford A, et al. Efficiently controlling for case-control imbalance and sample relatedness in large-scale genetic association studies. *Nat Genet* (2018) 50:1335–1341.

44. Malik R, Chauhan G, Traylor M, Sargurupremraj M, Okada Y, Mishra A, Rutten-Jacobs L, Giese AK, van der Laan SW, Gretarsdottir S, et al. Multiancestry genome-wide association study of 520,000 subjects identifies 32 loci associated with stroke and stroke subtypes. *Nat Genet* (2018) 50: doi: 10.1038/s41588-018-0058-3

45. Loci associated with ischaemic stroke and its subtypes (SiGN): a genome-wide association study. *Lancet Neurol* (2016) 15: doi: 10.1016/S1474-4422(15)00338-5

46. von Berg J, van der Laan SW, McArdle PF, Malik R, Kittner SJ, Mitchell BD, Worrall BB, de Ridder J, Pulit SL. Alternate approach to stroke phenotyping identifies a genetic risk locus for small vessel stroke. *Eur J Hum Genet* (2020) 28:963–972.

47. van der Harst P, Verweij N. Identification of 64 Novel Genetic Loci Provides an Expanded View on the Genetic Architecture of Coronary Artery Disease. *Circ Res* (2018) 122: doi: 10.1161/CIRCRESAHA.117.312086

48. Klarin D, Lynch J, Aragam K, Chaffin M, Assimes TL, Huang J, Lee KM, Shao Q, Huffman JE, Natarajan P, et al. Genome-wide association study of peripheral artery disease in the Million Veteran Program. *Nat Med* (2019) 25:1274–1279.

49. Nelson CP, Goel A, Butterworth AS, Kanoni S, Webb TR, Marouli E, Zeng L, Ntalla I, Lai FY, Hopewell JC, et al. Association analyses based on false discovery rate implicate new loci for

coronary artery disease. *Nat Genet* (2017) 49: doi: 10.1038/ng.3913

50. Giri A, Hellwege JN, Keaton JM, Park J, Qiu C, Warren HR, Torstenson ES, Kovesdy CP, Sun YV, Wilson OD, et al. Trans-ethnic association study of blood pressure determinants in over 750,000 individuals. *Nat Genet* (2019) 51: doi: 10.1038/s41588-018-0303-9

51. Takeuchi F, Akiyama M, Matoba N, Katsuya T, Nakatochi M, Tabara Y, Narita A, Saw WY, Moon S, Spracklen CN, et al. Interethnic analyses of blood pressure loci in populations of East Asian and European descent. *Nat Commun* (2018) 9: doi: 10.1038/s41467-018-07345-0

52. Kato N, Loh M, Takeuchi F, Verweij N, Wang X, Zhang W, Kelly TN, Saleheen D, Lehne B, Leach IM, et al. Trans-ancestry genome-wide association study identifies 12 genetic loci influencing blood pressure and implicates a role for DNA methylation. *Nat Genet* (2015) 47: doi: 10.1038/ng.3405

53. Duan L, Wei L, Tian Y, Zhang Z, Hu P, Wei Q, Liu S, Zhang J, Wang Y, Li D, et al. Novel Susceptibility Loci for Moyamoya Disease Revealed by a Genome-Wide Association Study. *Stroke* (2018) 49: doi: 10.1161/STROKEAHA.117.017430

54. Pirruccello JP, Chaffin MD, Fleming SJ, Arduini A, Lin H, Khurshid S, Chou EL, Friedman SN, Bick AG, Weng L-C, et al. Deep learning enables genetic analysis of the human thoracic aorta. (2020)2020.05.12.091934. doi: 10.1101/2020.05.12.091934

55. Pinard A, Jones GT, Milewicz DM. Genetics of Thoracic and Abdominal Aortic Diseases. *Circ Res* (2019) 124:588–606.

56. Thériault Sébastien, Dina Christian, Messika-Zeitoun David, Le Scouarnec Solena, Capoulade Romain, Gaudreault Nathalie, Rigade Sidwell, Li Zhonglin, Simonet Floriane, Lamontagne Maxime, et al. Genetic Association Analyses Highlight IL6, ALPL, and NAV1 As 3 New Susceptibility Genes Underlying Calcific Aortic Valve Stenosis. *Circulation: Genomic and Precision Medicine* (2019) 12:e002617.

57. Eichner JE, Dunn ST, Perveen G, Thompson DM, Stewart KE, Stroehla BC. Apolipoprotein E polymorphism and cardiovascular disease: a HuGE review. *Am J Epidemiol* (2002) 155:487–495.
