# Supplementary material for: Calcification of the abdominal aorta is an under-appreciated cardiovascular disease risk factor in the general population
Source: Front Cardiovasc Med. 2022 Oct 6;9:1003246. doi: 10.3389/fcvm.2022.1003246 (PMC9582957; doi:10.3389/fcvm.2022.1003246)
Supplement: Supplementary file 2 [file Data_Sheet_2.PDF]

AAC Analysis Supporting Tables

Table S1: Comparison of Manual Annotation on Training Set **2**

Table S2: Comparison of Manual Annotation on Validation Set **3**

Table S3: Accuracy of machine learning pipelines. **4**

Table S4: Comparison of Phenotypic Correlation to AAC in UKBB and MrOS **5**

Table S5: Univariate Associations of Biomarkers to AAC **6**

Table S6: Univariate Associations of Physiological Function to AAC **8**

Table S7: Association of predicted AAC with complete blood count markers. **17**

Table S8: Fine Mapping of GWAS Signal **18**

Table S9: Meta-analysis **21**

Table S10: Genetic correlation between AAC and 773 complex traits calculated using LD Score Regression. **22**

Table S11: Colocalization of AAC signals with expression in different tissues. **23**

Table S12: Colocalization of AAC with complex traits in UK Biobank. **25**

Table S13: Colocalization of AAC signal with cardiovascular traits from independent studies. **26**

Table S14: Disease codes and prognostic event counts **28**

Table S1: Comparison of Manual Annotation on Training Set

Variability of manual annotation over the training set (n=1000) for the 4 different annotators measured using (A) Pearson Correlation and (B) Spearman correlation.

A)

	Annotator 1	Annotator 2	Annotator 3	Annotator 4	Median
Annotator 1	1	0.84	0.72	0.74	0.92
Annotator 2	0.84	1	0.75	0.78	0.93
Annotator 3	0.72	0.75	1	0.69	0.85
Annotator 4	0.74	0.78	0.69	1	0.86
Median	0.92	0.93	0.85	0.86	1

B)

	Annotator 1	Annotator 2	Annotator 3	Annotator 4	Median
Annotator 1	1	0.61	0.56	0.53	0.74
Annotator 2	0.61	1	0.57	0.53	0.8
Annotator 3	0.56	0.57	1	0.55	0.79
Annotator 4	0.53	0.53	0.55	1	0.75
Median	0.74	0.8	0.79	0.75	1

Table S2: Comparison of Manual Annotation on Validation Set

Variability of manual annotation over the validation set (n=300) for the 4 different annotators measured using (A) Pearson Correlation and (B) Spearman correlation.

A.

	Annotator 1	Annotator 2	Annotator 3	Annotator 4	Median
Annotator 1	1	0.86	0.78	0.75	0.94
Annotator 2	0.86	1	0.73	0.67	0.93
Annotator 3	0.78	0.73	1	0.7	0.85
Annotator 4	0.75	0.67	0.7	1	0.73
Median	0.94	0.93	0.85	0.73	1

B.

	Annotator 1	Annotator 2	Annotator 3	Annotator 4	Median
Annotator 1	1	0.68	0.67	0.56	0.83
Annotator 2	0.68	1	0.71	0.51	0.88
Annotator 3	0.67	0.71	1	0.55	0.84
Annotator 4	0.56	0.51	0.55	1	0.57
Median	0.83	0.88	0.84	0.57	1

Table S3: Accuracy of machine learning pipelines.

The accuracy of the machine learning pipelines and the ensemble model were calculated using Pearsons' and Spearman's correlation coefficient over the test dataset (300 images). Correlation of each individual annotator with the median for the test dataset is also shown for comparison.

	**Pearson correlation with median score**	**Spearman correlation with median score**
Annotator 1	0.94	0.83
Annotator 2	0.93	0.88
Annotator 3	0.85	0.84
Annotator 4	0.73	0.57
Machine Learning Models		
Model 1	0.62	0.41
Model 2	0.59	0.47
Ensemble Model	0.67	0.49

Table S4: Comparison of Phenotypic Correlation to AAC in UKBB and MrOS

Correlation of annotated abdominal aortic calcification with different phenotypes across MrOS (n=4800) and UKBB cohorts (n=38,264). Only phenotypes significantly correlated with aortic calcification in MrOS cohort are displayed in the above table.

	MrOS Cohort	UKBB Cohort
Phenotype	Correlation	Correlation
Age	0.3	0.28
Systolic Blood Pressure	0.15	0.18
Pulse	0.09	0.02
Glucose	0.08	0.06
Cystatin C	0.12	0.09
HDL	-0.06	-0.01
Phosphate	0.17	0.05
Triglycerides	0.1	0.04

Table S5: Univariate Associations of Biomarkers to AAC

Association of predicted AAC with biomarkers Univariate regression analysis of risk factors at baseline for predicted AAC (n=38,264) after adjusting for age and sex in model 1 and after adjusting for socioeconomic factors, BMI, and smoking status in addition to adjusting for age and sex in model 2. The estimate of the effect size and the standard error of the estimated effect size are given along with p-values for each coefficient in a univariate fit.

	Model 1		Model 2	
Biomarker	Effect size	P-value	Effect size	P-value
HDL cholesterol	-0.041 (-0.05)	2.46E-09	-0.061 (-0.07)	1.33E-19
Creatinine	-0.040 (-0.05)	3.03E-07	-0.026 (-0.04)	3.53E-02
Apolipoprotein A	-0.031 (-0.04)	4.77E-05	-0.046 (-0.06)	1.60E-11
Testosterone	-0.027 (-0.06)	1.00E+00	-0.044 (-0.08)	1.00E+00
Urea	-0.016 (-0.03)	1.00E+00	-0.009 (-0.02)	1.00E+00
Total bilirubin	-0.014 (-0.02)	1.00E+00	-0.010 (-0.02)	1.00E+00
Rheumatoid factor	-0.005 (-0.04)	1.00E+00	-0.004 (-0.04)	1.00E+00
Cholesterol	-0.005 (-0.01)	1.00E+00	-0.006 (-0.02)	1.00E+00
Direct bilirubin	-0.005 (-0.02)	1.00E+00	-0.001 (-0.01)	1.00E+00
Vitamin D	-0.004 (-0.01)	1.00E+00	-0.009 (-0.02)	1.00E+00
IGF-1	-0.003 (-0.01)	1.00E+00	0.000 (-0.01)	1.00E+00
Oestradiol	-0.003 (-0.02)	1.00E+00	-0.006 (-0.03)	1.00E+00
Alkaline phosphatase	-0.000 (-0.01)	1.00E+00	0.005 (-0.01)	1.00E+00
LDL direct	0.001 (-0.01)	1.00E+00	0.004 (-0.01)	1.00E+00
Urate	0.003 (-0.01)	1.00E+00	0.018 (0.01)	1.00E+00
Lipoprotein A	0.003 (-0.01)	1.00E+00	0.004 (-0.01)	1.00E+00
Aspartate aminotransferase	0.005 (-0.01)	1.00E+00	0.011 (0.00)	1.00E+00
Cystatin C	0.007 (-0.00)	1.00E+00	0.011 (-0.00)	1.00E+00
Alanine aminotransferase	0.007 (-0.00)	1.00E+00	0.019 (0.01)	3.27E-01
C-reactive protein	0.010 (0.00)	1.00E+00	0.020 (0.01)	1.20E-01
Albumin	0.014 (0.00)	1.00E+00	0.012 (0.00)	1.00E+00

SHBG	0.016 (0.00)	1.00E+00	-0.001 (-0.01)	1.00E+00
Total protein	0.019 (0.01)	1.40E-01	0.028 (0.02)	8.58E-05
Gamma glutamyltransferase	0.023 (0.01)	1.53E-02	0.028 (0.02)	2.85E-04
Apolipoprotein B	0.024 (0.01)	1.31E-03	0.027 (0.02)	8.03E-05
Triglycerides	0.027 (0.02)	1.51E-04	0.036 (0.02)	6.95E-08
Calcium	0.028 (0.02)	3.51E-05	0.028 (0.02)	4.20E-05
Glucose	0.035 (0.02)	2.02E-08	0.040 (0.03)	1.53E-11
Phosphate	0.056 (0.05)	1.97E-22	0.051 (0.04)	2.17E-18
Glycated haemoglobin (HbA1c)	0.070 (0.06)	2.43E-39	0.075 (0.06)	5.58E-43

Table S6: Univariate Associations of Physiological Function to AAC

Association of predicted AAC with physiological markers. Univariate regression analysis of risk factors at baseline for predicted AAC (n=38,264) after adjusting for age and sex in model 1 and after adjusting for socioeconomic factors, BMI, and smoking status in addition to adjusting for age and sex in model 2. The estimate of the effect size and the standard error of the estimated effect size are given along with p-values for each coefficient in a univariate fit.

	Model 1		Model 2	
Physiological Measure	Effect size	P-value	Effect size	P-value
Heel bone mineral density (BMD) T-score, manual entry (right)	-0.158 (-0.35)	1.00E+00	-0.131 (-0.33)	1.00E+00
Heel bone mineral density (BMD) T-score, manual entry (left)	-0.143 (-0.33)	1.00E+00	-0.086 (-0.28)	1.00E+00
Amount of tobacco currently smoked	-0.112 (-0.23)	1.00E+00	-0.101 (-0.22)	1.00E+00
Whole body fat-free mass	-0.105 (-0.12)	1.05E-26	-0.115 (-0.14)	3.10E-19
Leg predicted mass (right)	-0.105 (-0.12)	1.61E-29	-0.121 (-0.14)	7.60E-21
Whole body water mass	-0.104 (-0.12)	1.26E-26	-0.114 (-0.14)	3.71E-19
Leg fat-free mass (right)	-0.103 (-0.12)	1.45E-29	-0.119 (-0.14)	7.25E-21
Forced expiratory volume in 1-second (FEV1), predicted	-0.100 (-0.14)	3.18E-03	-0.095 (-0.14)	5.83E-03
Arm predicted mass (right)	-0.100 (-0.12)	2.38E-22	-0.108 (-0.13)	1.54E-15
Leg predicted mass (left)	-0.098 (-0.12)	9.96E-27	-0.119 (-0.14)	6.99E-19
Arm fat-free mass (right)	-0.098 (-0.12)	3.98E-22	-0.106 (-0.13)	2.98E-15
Trunk predicted mass	-0.098 (-0.12)	2.53E-22	-0.095 (-0.12)	2.08E-15
Trunk fat-free mass	-0.097 (-0.12)	9.34E-23	-0.097 (-0.12)	2.69E-16
Leg fat-free mass (left)	-0.096 (-0.11)	1.79E-26	-0.116 (-0.14)	1.29E-18
Basal metabolic rate	-0.090 (-0.11)	8.99E-25	-0.108 (-0.13)	9.76E-18
Arm fat-free mass (left)	-0.088 (-0.11)	1.52E-19	-0.094 (-0.12)	8.52E-13
Number of cigarettes previously smoked daily (current cigar/pipe smokers)	-0.084 (-0.32)	1.00E+00	-0.099 (-0.36)	1.00E+00
Arm predicted mass (left)	-0.081 (-0.10)	9.48E-18	-0.081 (-0.10)	1.05E-10
Forced expiratory volume in 1-second (FEV1), Best measure	-0.077 (-0.09)	2.28E-20	-0.073 (-0.09)	2.19E-18

Heel quantitative ultrasound index (QUI), manual entry (left)	-0.072 (-0.27)	1.00E+00	-0.080 (-0.28)	1.00E+00
Maximum workload during fitness test	-0.071 (-0.10)	5.42E-04	-0.074 (-0.10)	1.00E+00
Heel bone mineral density (BMD), manual entry (left)	-0.070 (-0.26)	1.00E+00	-0.077 (-0.27)	1.00E+00
Heel broadband ultrasound attenuation (left)	-0.064 (-0.08)	8.04E-11	-0.058 (-0.08)	3.54E-08
Weight (pre-imaging)	-0.064 (-0.08)	1.34E-15	-0.094 (-0.12)	3.45E-12
Forced expiratory volume in 1-second (FEV1), predicted percentage	-0.064 (-0.08)	1.43E-10	-0.038 (-0.05)	6.32E-03
Heel bone ultrasound T-score, manual entry	-0.063 (-0.09)	1.12E-02	-0.052 (-0.08)	2.21E-01
Heel bone mineral density (BMD) (left)	-0.062 (-0.08)	2.09E-10	-0.055 (-0.07)	8.32E-08
Heel quantitative ultrasound index (QUI), direct entry (left)	-0.062 (-0.08)	2.04E-10	-0.055 (-0.07)	8.51E-08
Heel bone mineral density (BMD) T-score, automated (left)	-0.062 (-0.08)	2.05E-10	-0.055 (-0.07)	8.53E-08
Forced expiratory volume in 1-second (FEV1)	-0.062 (-0.08)	4.50E-17	-0.064 (-0.08)	2.21E-17
Heel broadband ultrasound attenuation (right)	-0.060 (-0.08)	3.67E-09	-0.051 (-0.07)	4.91E-06
Forced vital capacity (FVC), Best measure	-0.060 (-0.08)	1.98E-11	-0.069 (-0.08)	1.01E-14
Heel bone mineral density (BMD) T-score, automated (right)	-0.056 (-0.07)	3.23E-08	-0.048 (-0.06)	1.20E-05
Heel quantitative ultrasound index (QUI), direct entry (right)	-0.056 (-0.07)	3.23E-08	-0.048 (-0.06)	1.20E-05
Heel bone mineral density (BMD) (right)	-0.056 (-0.07)	3.49E-08	-0.049 (-0.07)	9.31E-06
Weight	-0.053 (-0.06)	1.13E-17	-0.103 (-0.13)	7.32E-14
Standing height	-0.052 (-0.07)	9.29E-11	-0.058 (-0.07)	4.23E-13
Height	-0.051 (-0.07)	2.01E-05	-0.055 (-0.07)	2.55E-06
Weight, manual entry	-0.047 (-0.15)	1.00E+00	-0.060 (-0.32)	1.00E+00
Forced vital capacity (FVC)	-0.047 (-0.06)	8.27E-09	-0.058 (-0.07)	1.34E-13
Non-cancer illness year/age first occurred	-0.044 (-0.06)	9.60E-10	-0.048 (-0.06)	4.59E-12
Heel broadband ultrasound attenuation (BUA), manual entry (left)	-0.044 (-0.25)	1.00E+00	-0.039 (-0.25)	1.00E+00
Number of trend entries	-0.043 (-0.07)	3.54E-01	-0.046 (-0.07)	1.74E-01
Father's age at death	-0.042 (-0.05)	2.94E-08	-0.040 (-0.05)	1.79E-07
Ankle spacing width	-0.041 (-0.06)	1.20E-04	-0.034 (-0.05)	1.44E-01

Heel Broadband ultrasound attenuation, direct entry	-0.040 (-0.05)	3.65E-06	-0.030 (-0.04)	7.92E-03
Heel bone mineral density (BMD)	-0.040 (-0.05)	2.10E-06	-0.031 (-0.04)	3.85E-03
Heel bone mineral density (BMD) T-score, automated	-0.040 (-0.05)	3.00E-06	-0.030 (-0.04)	5.13E-03
Heel quantitative ultrasound index (QUI), direct entry	-0.040 (-0.05)	3.00E-06	-0.030 (-0.04)	5.13E-03
Heel quantitative ultrasound index (QUI), manual entry	-0.039 (-0.07)	1.00E+00	-0.029 (-0.06)	1.00E+00
Hip circumference	-0.038 (-0.05)	2.93E-12	-0.037 (-0.05)	2.04E-02
Heel bone mineral density (BMD), manual entry	-0.038 (-0.07)	1.00E+00	-0.027 (-0.06)	1.00E+00
Hand grip strength (left)	-0.037 (-0.05)	3.17E-04	-0.038 (-0.05)	1.79E-04
Sitting height	-0.035 (-0.05)	1.73E-05	-0.033 (-0.05)	1.59E-04
Leg fat mass (left)	-0.035 (-0.05)	4.75E-06	-0.004 (-0.04)	1.00E+00
Arm fat mass (left)	-0.035 (-0.04)	3.03E-09	-0.030 (-0.06)	1.00E+00
Number of symbol digit matches attempted	-0.035 (-0.05)	2.07E-03	-0.030 (-0.05)	2.97E-02
Arm fat mass (right)	-0.034 (-0.04)	6.29E-09	-0.029 (-0.06)	1.00E+00
Number of symbol digit matches made correctly	-0.034 (-0.05)	2.22E-03	-0.031 (-0.05)	1.76E-02
Ankle spacing width (right)	-0.034 (-0.05)	2.11E-01	-0.035 (-0.06)	1.00E+00
Errors before selecting correct item in alphanumeric path (trail #2)	-0.034 (-0.09)	1.00E+00	-0.036 (-0.09)	1.00E+00
Leg fat mass (right)	-0.033 (-0.05)	2.61E-05	0.007 (-0.03)	1.00E+00
Hand grip strength (right)	-0.033 (-0.05)	3.34E-03	-0.033 (-0.05)	4.33E-03
Peak expiratory flow (PEF)	-0.032 (-0.04)	9.61E-05	-0.028 (-0.04)	3.38E-03
Genetic relatedness pairing	-0.031 (-0.08)	1.00E+00	-0.026 (-0.08)	1.00E+00
Whole body fat mass	-0.031 (-0.04)	8.44E-07	-0.013 (-0.04)	1.00E+00
Ankle spacing width (left)	-0.030 (-0.05)	1.00E+00	-0.030 (-0.05)	1.00E+00
Trunk fat mass	-0.029 (-0.04)	1.87E-06	-0.010 (-0.03)	1.00E+00
Duration of fitness test	-0.028 (-0.05)	1.00E+00	-0.031 (-0.05)	1.00E+00
Arm fat percentage (left)	-0.028 (-0.04)	1.69E-02	0.049 (0.02)	2.72E-01
Years of bringing up phlegm/sputum/mucus on most days	-0.025 (-0.08)	1.00E+00	-0.012 (-0.06)	1.00E+00
Waist circumference	-0.025 (-0.04)	6.75E-03	0.008 (-0.01)	1.00E+00
Arm fat percentage (right)	-0.024 (-0.04)	1.76E-01	0.060 (0.03)	7.38E-03

Pulse wave reflection index	-0.021 (-0.04)	1.00E+00	-0.022 (-0.04)	1.00E+00
Cascot confidence score	-0.021 (-0.06)	1.00E+00	-0.017 (-0.06)	1.00E+00
Fluid intelligence score.1	-0.020 (-0.03)	1.00E+00	-0.018 (-0.03)	1.00E+00
Fluid intelligence score	-0.020 (-0.04)	1.00E+00	-0.018 (-0.03)	1.00E+00
Heel Broadband ultrasound attenuation (BUA), manual entry	-0.019 (-0.05)	1.00E+00	-0.009 (-0.04)	1.00E+00
Thursday average acceleration	-0.019 (-0.03)	1.00E+00	-0.024 (-0.04)	6.50E-01
Average acceleration 12:00 - 12:59	-0.019 (-0.03)	1.00E+00	-0.021 (-0.04)	1.00E+00
QRS duration	-0.019 (-0.03)	1.00E+00	-0.015 (-0.03)	1.00E+00
Errors before selecting correct item in numeric path (trail 1)	-0.018 (-0.11)	1.00E+00	-0.010 (-0.10)	1.00E+00
Average acceleration 23:00 - 23:59	-0.018 (-0.03)	1.00E+00	-0.019 (-0.03)	1.00E+00
Body fat percentage	-0.017 (-0.03)	1.00E+00	0.057 (0.03)	1.05E-03
Friday average acceleration	-0.016 (-0.03)	1.00E+00	-0.020 (-0.03)	1.00E+00
Wednesday average acceleration	-0.016 (-0.03)	1.00E+00	-0.019 (-0.03)	1.00E+00
Overall acceleration average	-0.016 (-0.03)	1.00E+00	-0.020 (-0.04)	1.00E+00
Average acceleration 22:00 - 22:59	-0.016 (-0.03)	1.00E+00	-0.016 (-0.03)	1.00E+00
Mother's age at death	-0.016 (-0.03)	1.00E+00	-0.017 (-0.03)	1.00E+00
Average acceleration 10:00 - 10:59	-0.015 (-0.03)	1.00E+00	-0.018 (-0.03)	1.00E+00
Average acceleration 17:00 - 17:59	-0.015 (-0.03)	1.00E+00	-0.018 (-0.03)	1.00E+00
Sunday average acceleration	-0.015 (-0.03)	1.00E+00	-0.018 (-0.03)	1.00E+00
Leg fat percentage (left)	-0.015 (-0.03)	1.00E+00	0.119 (0.08)	2.13E-07
ECG, number of stages in a phase	-0.014 (-0.04)	1.00E+00	-0.014 (-0.04)	1.00E+00
No-wear time bias adjusted average acceleration	-0.014 (-0.03)	1.00E+00	-0.019 (-0.03)	1.00E+00
Saturday average acceleration	-0.014 (-0.03)	1.00E+00	-0.015 (-0.03)	1.00E+00
Trunk fat percentage	-0.014 (-0.02)	1.00E+00	0.029 (0.01)	3.20E-01
Average acceleration 14:00 - 14:59	-0.014 (-0.03)	1.00E+00	-0.016 (-0.03)	1.00E+00
Tuesday average acceleration	-0.013 (-0.03)	1.00E+00	-0.016 (-0.03)	1.00E+00
Average acceleration 11:00 - 11:59	-0.013 (-0.03)	1.00E+00	-0.016 (-0.03)	1.00E+00
Monday average acceleration	-0.013 (-0.03)	1.00E+00	-0.016 (-0.03)	1.00E+00
Genetic principal components	-0.012 (-0.02)	1.00E+00	0.012 (-0.02)	1.00E+00
Pulse wave peak to peak time	-0.012 (-0.03)	1.00E+00	-0.009 (-0.03)	1.00E+00
Average acceleration 16:00 - 16:59	-0.012 (-0.03)	1.00E+00	-0.013 (-0.03)	1.00E+00
Maximum digits remembered correctly.1	-0.012 (-0.03)	1.00E+00	-0.013 (-0.03)	1.00E+00

Average acceleration 15:00 - 15:59	-0.012 (-0.03)	1.00E+00	-0.014 (-0.03)	1.00E+00
Total errors traversing numeric path (trail #1)	-0.012 (-0.04)	1.00E+00	-0.011 (-0.04)	1.00E+00
Number of correct matches in round.1	-0.012 (-0.03)	1.00E+00	-0.010 (-0.02)	1.00E+00
Years of cough on most days	-0.011 (-0.05)	1.00E+00	-0.001 (-0.04)	1.00E+00
Average acceleration 09:00 - 09:59	-0.011 (-0.03)	1.00E+00	-0.015 (-0.03)	1.00E+00
P duration	-0.011 (-0.02)	1.00E+00	-0.008 (-0.02)	1.00E+00
No-wear time bias adjusted acceleration standard deviation	-0.011 (-0.03)	1.00E+00	-0.014 (-0.03)	1.00E+00
Genetic relatedness IBS0	-0.010 (-0.06)	1.00E+00	-0.013 (-0.07)	1.00E+00
Standard deviation of acceleration	-0.010 (-0.02)	1.00E+00	-0.013 (-0.03)	1.00E+00
Average acceleration 13:00 - 13:59	-0.010 (-0.02)	1.00E+00	-0.013 (-0.03)	1.00E+00
Average acceleration 21:00 - 21:59	-0.010 (-0.02)	1.00E+00	-0.012 (-0.03)	1.00E+00
Leg fat percentage (right)	-0.008 (-0.03)	1.00E+00	0.120 (0.09)	4.28E-09
Number of times snap-button pressed	-0.007 (-0.02)	1.00E+00	-0.007 (-0.02)	1.00E+00
Number of fluid intelligence questions attempted within time limit	-0.007 (-0.02)	1.00E+00	-0.003 (-0.02)	1.00E+00
Value entered	-0.007 (-0.02)	1.00E+00	-0.004 (-0.02)	1.00E+00
Number of incorrect matches in round	-0.006 (-0.02)	1.00E+00	-0.006 (-0.02)	1.00E+00
Heel bone mineral density (BMD), manual entry (right)	-0.005 (-0.20)	1.00E+00	-0.052 (-0.25)	1.00E+00
Number of days/week of vigorous physical activity 10+ minutes	-0.005 (-0.01)	1.00E+00	-0.008 (-0.02)	1.00E+00
Number of days/week of moderate physical activity 10+ minutes	-0.005 (-0.01)	1.00E+00	-0.011 (-0.02)	1.00E+00
Duration of vigorous activity	-0.004 (-0.02)	1.00E+00	-0.006 (-0.02)	1.00E+00
Maximum heart rate during fitness test	-0.004 (-0.03)	1.00E+00	0.000 (-0.02)	1.00E+00
Average acceleration 03:00 - 03:59	-0.004 (-0.02)	1.00E+00	-0.006 (-0.02)	1.00E+00
Diastolic blood pressure, automated reading	-0.004 (-0.01)	1.00E+00	0.009 (-0.00)	1.00E+00
Average acceleration 00:00 - 00:59	-0.004 (-0.02)	1.00E+00	-0.007 (-0.02)	1.00E+00
Genetic relatedness factor	-0.004 (-0.06)	1.00E+00	-0.001 (-0.05)	1.00E+00
Average acceleration 20:00 - 20:59	-0.004 (-0.02)	1.00E+00	-0.005 (-0.02)	1.00E+00
Missingness	-0.003 (-0.01)	1.00E+00	-0.003 (-0.01)	1.00E+00
Average acceleration 18:00 - 18:59	-0.003 (-0.02)	1.00E+00	-0.004 (-0.02)	1.00E+00
Maximum digits remembered correctly	-0.003 (-0.03)	1.00E+00	-0.003 (-0.03)	1.00E+00
Average acceleration 07:00 - 07:59	-0.003 (-0.02)	1.00E+00	-0.000 (-0.01)	1.00E+00

Average Y chromosome intensities for determining sex	-0.003 (-0.03)	1.00E+00	-0.005 (-0.03)	1.00E+00
Time to complete round	-0.003 (-0.01)	1.00E+00	-0.002 (-0.01)	1.00E+00
Duration to first press of snap-button in each round	-0.002 (-0.02)	1.00E+00	-0.002 (-0.02)	1.00E+00
Average acceleration 01:00 - 01:59	-0.002 (-0.02)	1.00E+00	-0.006 (-0.02)	1.00E+00
Average acceleration 08:00 - 08:59	-0.002 (-0.02)	1.00E+00	-0.004 (-0.02)	1.00E+00
No-wear time bias adjusted acceleration minimum	-0.002 (-0.02)	1.00E+00	-0.003 (-0.02)	1.00E+00
Time to answer	-0.001 (-0.02)	1.00E+00	-0.003 (-0.02)	1.00E+00
Average acceleration 19:00 - 19:59	-0.001 (-0.02)	1.00E+00	-0.001 (-0.02)	1.00E+00
Average acceleration 02:00 - 02:59	-0.001 (-0.01)	1.00E+00	-0.005 (-0.02)	1.00E+00
No-wear time bias adjusted acceleration median	-0.001 (-0.02)	1.00E+00	-0.002 (-0.02)	1.00E+00
Number of days/week walked 10+ minutes	-0.000 (-0.01)	1.00E+00	-0.006 (-0.02)	1.00E+00
Average acceleration 04:00 - 04:59	-0.000 (-0.01)	1.00E+00	-0.001 (-0.02)	1.00E+00
Birth weight	-0.000 (-0.01)	1.00E+00	-0.004 (-0.02)	1.00E+00
No-wear time bias adjusted acceleration maximum	0.000 (-0.01)	1.00E+00	-0.001 (-0.02)	1.00E+00
Number of correct matches in round	0.001 (-0.01)	1.00E+00	0.001 (-0.01)	1.00E+00
Average X chromosome intensities for determining sex	0.002 (-0.02)	1.00E+00	-0.001 (-0.02)	1.00E+00
Time to complete round.1	0.002 (-0.01)	1.00E+00	0.000 (-0.01)	1.00E+00
Average acceleration 06:00 - 06:59	0.002 (-0.01)	1.00E+00	0.002 (-0.01)	1.00E+00
Interval between previous point and current one in alphanumeric path (trail #2)	0.003 (-0.01)	1.00E+00	0.002 (-0.01)	1.00E+00
Heterozygosity, PCA corrected	0.003 (-0.01)	1.00E+00	0.004 (-0.01)	1.00E+00
Number of incorrect matches in round.1	0.003 (-0.01)	1.00E+00	0.001 (-0.01)	1.00E+00
Particulate matter air pollution (pm10); 2010	0.003 (-0.01)	1.00E+00	-0.002 (-0.01)	1.00E+00
Mean time to correctly identify matches	0.003 (-0.01)	1.00E+00	0.003 (-0.01)	1.00E+00
Duration to complete numeric path (trail #1)	0.004 (-0.01)	1.00E+00	0.002 (-0.01)	1.00E+00
Maximum carotid IMT (intima-medial thickness) at 150 degrees	0.004 (-0.04)	1.00E+00	0.002 (-0.04)	1.00E+00
Particulate matter air pollution 2.5-10um; 2010	0.004 (-0.01)	1.00E+00	0.002 (-0.01)	3.23E+02
Interval between previous point and current one in numeric path (trail #1)	0.004 (-0.01)	1.00E+00	0.005 (-0.01)	1.00E+00

Total errors traversing alphanumeric path (trail #2)	0.004 (-0.02)	1.00E+00	0.003 (-0.02)	1.00E+00
Diastolic blood pressure, manual reading	0.005 (-0.03)	1.00E+00	0.017 (-0.02)	1.00E+00
Duration of moderate activity	0.005 (-0.01)	1.00E+00	-0.001 (-0.01)	1.00E+00
Signal-to-noise-ratio (SNR) of triplet (left)	0.006 (-0.01)	1.00E+00	0.007 (-0.01)	1.00E+00
Signal-to-noise-ratio (SNR) of triplet (right)	0.006 (-0.01)	1.00E+00	0.007 (-0.01)	1.00E+00
ECG, phase time	0.006 (-0.02)	1.00E+00	0.004 (-0.02)	1.00E+00
Average acceleration 05:00 - 05:59	0.006 (-0.01)	1.00E+00	0.005 (-0.01)	1.00E+00
logMAR, final (right)	0.007 (-0.01)	1.00E+00	0.009 (-0.01)	1.00E+00
Heterozygosity	0.007 (-0.00)	1.00E+00	-0.002 (-0.02)	1.00E+00
Number of full sisters	0.008 (-0.00)	1.00E+00	0.003 (-0.01)	1.00E+00
Nitrogen dioxide air pollution; 2010	0.008 (-0.00)	1.00E+00	-0.008 (-0.02)	1.00E+00
Interpolated Year when non-cancer illness first diagnosed	0.009 (-0.00)	1.00E+00	0.008 (-0.00)	1.00E+00
Particulate matter air pollution (pm2.5) absorbance; 2010	0.009 (-0.00)	1.00E+00	-0.002 (-0.01)	1.00E+00
Interpolated Year when cancer first diagnosed	0.009 (-0.03)	1.00E+00	0.018 (-0.02)	1.00E+00
Nitrogen oxides air pollution; 2010	0.009 (-0.00)	1.00E+00	-0.005 (-0.02)	1.00E+00
Time spent driving	0.010 (-0.00)	1.00E+00	0.010 (-0.00)	1.00E+00
Sleep duration	0.010 (0.00)	1.00E+00	0.009 (-0.00)	1.00E+00
Particulate matter air pollution (pm2.5); 2010	0.010 (0.00)	1.00E+00	-0.003 (-0.01)	1.00E+00
Number of attempts	0.010 (-0.01)	1.00E+00	0.010 (-0.01)	1.00E+00
Time to complete test	0.011 (-0.02)	1.00E+00	0.008 (-0.02)	1.00E+00
Duration of walks	0.011 (0.00)	1.00E+00	0.006 (-0.00)	1.00E+00
Duration to entering value	0.011 (-0.00)	1.00E+00	0.010 (-0.00)	1.00E+00
Number of self-reported cancers	0.012 (0.00)	1.00E+00	0.010 (0.00)	1.00E+00
Pulse rate	0.013 (-0.00)	1.00E+00	0.018 (0.00)	1.00E+00
Number of triplets attempted (right)	0.013 (-0.00)	1.00E+00	0.016 (-0.00)	1.00E+00
Number of triplets attempted (left)	0.014 (-0.00)	1.00E+00	0.016 (-0.00)	1.00E+00
Number of automated diagnostic comments recorded during 12-lead ECG	0.014 (0.00)	1.00E+00	0.012 (-0.00)	1.00E+00
Number of full brothers	0.014 (0.00)	1.00E+00	0.009 (-0.00)	1.00E+00
Speech-reception-threshold (SRT) estimate (left)	0.014 (-0.00)	1.00E+00	0.013 (-0.00)	1.00E+00

Minimum carotid IMT (intima-medial thickness) at 150 degrees	0.014 (-0.03)	1.00E+00	0.011 (-0.03)	1.00E+00
Time spent using computer	0.015 (0.01)	1.00E+00	0.013 (0.00)	1.00E+00
Pulse wave Arterial Stiffness index	0.016 (-0.00)	1.00E+00	0.013 (-0.00)	1.00E+00
Number of operations, self-reported	0.016 (0.01)	6.01E-01	0.016 (0.01)	8.55E-01
Speech-reception-threshold (SRT) estimate (right)	0.017 (0.00)	1.00E+00	0.015 (-0.00)	1.00E+00
Duration screen displayed	0.017 (-0.02)	1.00E+00	0.018 (-0.02)	1.00E+00
Pulse rate, automated reading	0.018 (0.01)	1.64E-01	0.022 (0.01)	8.92E-03
Number of older siblings	0.018 (0.00)	1.00E+00	0.010 (-0.01)	1.00E+00
Pulse rate (during blood-pressure measurement)	0.019 (-0.02)	1.00E+00	0.019 (-0.02)	1.00E+00
Cancer year/age first occurred	0.020 (-0.02)	1.00E+00	0.021 (-0.02)	1.00E+00
ECG, phase duration	0.022 (-0.00)	1.00E+00	0.023 (-0.00)	1.00E+00
Ventricular rate	0.022 (0.01)	5.10E-01	0.024 (0.01)	2.32E-01
logMAR, final (left)	0.022 (0.00)	1.00E+00	0.020 (0.00)	1.00E+00
Time spent watching television (TV)	0.024 (0.01)	3.74E-04	0.025 (0.02)	1.37E-04
Duration to complete alphanumeric path (trail #2)	0.025 (0.01)	4.27E-01	0.022 (0.01)	1.00E+00
Heel quantitative ultrasound index (QUI), manual entry (right)	0.025 (-0.17)	1.00E+00	-0.012 (-0.21)	1.00E+00
Mean carotid IMT (intima-medial thickness) at 150 degrees	0.026 (-0.01)	1.00E+00	0.023 (-0.02)	1.00E+00
Reported occurrences of cancer	0.030 (-0.00)	1.00E+00	0.029 (-0.00)	1.00E+00
Minimum carotid IMT (intima-medial thickness) at 120 degrees	0.035 (-0.00)	1.00E+00	0.029 (-0.01)	1.00E+00
Minimum carotid IMT (intima-medial thickness) at 240 degrees	0.037 (-0.00)	1.00E+00	0.025 (-0.01)	1.00E+00
Impedance of arm (left)	0.039 (0.02)	7.07E-05	0.027 (0.01)	8.39E-01
Spells in hospital	0.041 (0.03)	5.97E-08	0.038 (0.03)	9.54E-07
Maximum carotid IMT (intima-medial thickness) at 120 degrees	0.043 (0.00)	1.00E+00	0.038 (-0.00)	1.00E+00
Impedance of arm (right)	0.045 (0.03)	4.83E-07	0.035 (0.02)	2.59E-02
ECG, heart rate	0.048 (0.03)	2.39E-02	0.053 (0.03)	5.20E-03
Mean carotid IMT (intima-medial thickness) at 120 degrees	0.048 (0.01)	1.00E+00	0.043 (0.00)	1.00E+00
Impedance of whole body	0.052 (0.04)	1.05E-11	0.044 (0.03)	1.06E-04

Impedance of leg (left)	0.053 (0.04)	3.56E-19	0.045 (0.03)	8.48E-09
Mean carotid IMT (intima-medial thickness) at 240 degrees	0.054 (0.01)	1.00E+00	0.040 (-0.00)	1.00E+00
Number of self-reported non-cancer illnesses	0.054 (0.04)	1.77E-25	0.058 (0.05)	9.44E-29
Impedance of leg (right)	0.057 (0.05)	4.75E-22	0.050 (0.04)	5.29E-11
Heel broadband ultrasound attenuation (BUA), manual entry (right)	0.058 (-0.15)	1.00E+00	0.020 (-0.19)	1.00E+00
Systolic blood pressure, manual reading	0.059 (0.02)	1.00E+00	0.070 (0.03)	1.98E-01
Medication for cholesterol, blood pressure, diabetes, or take exogenous hormones	0.060 (0.05)	1.74E-16	0.060 (0.05)	8.81E-17
Pulse wave velocity (manual entry)	0.061 (-0.71)	1.00E+00	0.019 (-0.50)	1.00E+00
Maximum carotid IMT (intima-medial thickness) at 240 degrees	0.062 (0.02)	1.00E+00	0.048 (0.01)	1.00E+00
Systolic blood pressure, automated reading	0.062 (0.05)	6.29E-28	0.074 (0.06)	5.77E-39
Maximum carotid IMT (intima-medial thickness) at 210 degrees	0.074 (0.03)	1.10E-01	0.061 (0.02)	1.00E+00
Number of treatments/medications taken	0.080 (0.07)	6.66E-54	0.084 (0.07)	6.17E-59
Minimum carotid IMT (intima-medial thickness) at 210 degrees	0.085 (0.05)	1.17E-02	0.073 (0.03)	1.24E-01
Mean carotid IMT (intima-medial thickness) at 210 degrees	0.085 (0.05)	1.43E-02	0.072 (0.03)	1.84E-01

Table S7: Association of predicted AAC with complete blood count markers.

Univariate regression analysis of risk factors at baseline for predicted AAC (n=38,264) after adjusting for age and sex in model 1 and after adjusting for socioeconomic factors, BMI, and smoking status in addition to adjusting for age and sex in model 2. The estimate of the effect size and the standard error of the estimated effect size are given along with p-values for each coefficient in a univariate fit.

	Model 1		Model 2	
CBC Measure	Effect size	P-value	Effect size	P-value
Red blood cell (erythrocyte) count	-0.025 (-0.04)	2.07E-02	-0.010 (-0.02)	1.00E+00
Lymphocyte percentage	-0.015 (-0.03)	1.00E+00	-0.014 (-0.02)	1.00E+00
Haemoglobin concentration	-0.011 (-0.02)	1.00E+00	-0.008 (-0.02)	1.00E+00
Platelet distribution width	-0.009 (-0.02)	1.00E+00	-0.006 (-0.02)	1.00E+00
Haematocrit percentage	-0.007 (-0.02)	1.00E+00	-0.003 (-0.02)	1.00E+00
Mean corpuscular haemoglobin concentration	-0.006 (-0.02)	1.00E+00	-0.007 (-0.02)	1.00E+00
Mean platelet (thrombocyte) volume	-0.006 (-0.02)	1.00E+00	-0.007 (-0.02)	1.00E+00
Nucleated red blood cell percentage	-0.002 (-0.01)	1.00E+00	-0.003 (-0.01)	1.00E+00
Monocyte percentage	-0.001 (-0.01)	1.00E+00	0.002 (-0.01)	1.00E+00
Nucleated red blood cell count	0.000 (-0.01)	1.00E+00	-0.001 (-0.01)	1.00E+00
Red blood cell (erythrocyte) distribution width	0.002 (-0.01)	1.00E+00	0.004 (-0.01)	1.00E+00
Eosinophill percentage	0.004 (-0.01)	1.00E+00	0.004 (-0.01)	1.00E+00
Mean reticulocyte volume	0.009 (-0.00)	1.00E+00	0.003 (-0.01)	1.00E+00
Basophill percentage	0.012 (0.00)	1.00E+00	0.009 (-0.00)	1.00E+00
Neutrophill percentage	0.012 (0.00)	1.00E+00	0.010 (0.00)	1.00E+00

Reticulocyte count	0.013 (0.00)	1.00E+00	0.021 (0.01)	3.12E-02
Mean sphered cell volume	0.015 (0.01)	1.00E+00	-0.000 (-0.01)	1.00E+00
Reticulocyte percentage	0.016 (0.01)	5.24E-01	0.023 (0.01)	2.75E-03
Mean corpuscular haemoglobin	0.019 (0.01)	7.10E-02	0.005 (-0.01)	1.00E+00
High light scatter reticulocyte count	0.020 (0.01)	3.23E-02	0.035 (0.02)	5.42E-08
Eosinophill count	0.021 (0.01)	2.07E-02	0.018 (0.01)	1.11E-01
Immature reticulocyte fraction	0.021 (0.01)	2.15E-02	0.028 (0.02)	3.12E-05
Basophill count	0.021 (0.01)	2.58E-02	0.017 (0.01)	4.40E-01
High light scatter reticulocyte percentage	0.023 (0.01)	1.87E-03	0.035 (0.02)	2.96E-08
Platelet count	0.024 (0.01)	1.48E-03	0.023 (0.01)	4.37E-03
Platelet crit	0.025 (0.01)	1.17E-03	0.023 (0.01)	4.64E-03
Mean corpuscular volume	0.027 (0.02)	5.82E-05	0.010 (0.00)	1.00E+00
Lymphocyte count	0.027 (0.02)	5.53E-05	0.024 (0.01)	1.44E-03
Monocyte count	0.034 (0.02)	1.54E-08	0.033 (0.02)	1.25E-07
Neutrophill count	0.050 (0.04)	4.64E-21	0.044 (0.03)	1.71E-15
White blood cell (leukocyte) count	0.056 (0.05)	1.34E-25	0.049 (0.04)	3.97E-19

Table S8: Fine Mapping of GWAS Signal

SNPs in 95% credible sets. Where there is a secondary signal, the var_conditional column gives the ID of the SNP conditioned on.

var index	var conditional	var id	rs id	var chr	var pos	effect allele	other allele	beta	ste	pv	pp
1:201690989:C:T	NA	1:201690545:G:T	rs475210	1	201690545	T	G	-0.041	0.007	1.21E-08	0.145
1:201690989:C:T	NA	1:201690989:C:T	rs560804	1	201690989	T	C	-0.041	0.007	9.30E-09	0.188
1:201690989:C:T	NA	1:201742382:G:T	rs480937	1	201742382	T	G	0.040	0.007	2.14E-08	0.084
1:201690989:C:T	NA	1:201743185:G:A	rs631556	1	201743185	A	G	0.040	0.007	2.13E-08	0.084
1:201690989:C:T	NA	1:201743938:G:A	rs516705	1	201743938	A	G	0.040	0.007	2.03E-08	0.089
1:201690989:C:T	NA	1:201745951:G:T	rs1256358	1	201745951	T	G	0.038	0.007	8.05E-08	0.023
1:201690989:C:T	NA	1:201748087:C:A	rs665834	1	201748087	A	C	0.040	0.007	1.92E-08	0.093
1:201690989:C:T	NA	1:201748124:G:A	rs665770	1	201748124	A	G	0.040	0.007	2.20E-08	0.082
1:201690989:C:T	NA	1:201748715:G:A	rs511763	1	201748715	A	G	0.040	0.007	2.07E-08	0.087
1:201690989:C:T	NA	1:201749156:G:T	rs650720	1	201749156	T	G	0.040	0.007	2.32E-08	0.077
13:22862729:C:T	NA	13:22862729:C:T	rs12863716	13	22862729	T	C	-0.051	0.008	1.21E-09	0.211
13:22862729:C:T	NA	13:22872349:C:T	rs12869493	13	22872349	T	C	-0.051	0.008	1.73E-09	0.149
13:22862729:C:T	NA	13:22877125:C:T	rs12876505	13	22877125	T	C	-0.051	0.008	1.86E-09	0.139
13:22862729:C:T	NA	13:22877463:G:A	rs12875918	13	22877463	A	G	-0.051	0.008	1.81E-09	0.143
13:22862729:C:T	NA	13:22880051:C:T	rs34348807	13	22880051	T	C	-0.051	0.008	1.67E-09	0.154
13:22862729:C:T	NA	13:22881442:C:T	rs113648283	13	22881442	T	C	-0.051	0.008	2.50E-09	0.105
13:22862729:	NA	13:22884524	rs1507721	13	22884524	T	C	-0.051	0.0	2.65	0.09

C:T		:C:T							08	E-09	9
19:45382675:<br>G:A	NA	19:45382675<br>:G:A	rs41290120	19	45382675	A	G	-0.099	0.0<br>17	2.85<br>E-09	0.97<br>4
7:19045397:A:<br>G	7:190493<br>88:G:A	7:19037661:<br>A:G	rs7798197	7	19037661	G	A	-0.044	0.0<br>09	9.32<br>E-07	0.04<br>5
7:19045397:A:<br>G	7:190493<br>88:G:A	7:19045397:<br>A:G	rs2526620	7	19045397	G	A	-0.049	0.0<br>09	4.14<br>E-08	0.90<br>6
7:19049388:G:<br>A	NA	7:19049388:<br>G:A	rs2107595	7	19049388	A	G	0.090	0.0<br>10	1.47<br>E-20	0.98<br>6

Table S9: Meta-analysis

Standardized effect size and p-value for the lead SNP at each locus in this study, the CHARGE consortium study, as well as the meta analysis of both studies. Values are also given for the lead SNP at the RAP1GAP locus identified in CHARGE even though this locus is not significant in our study or the meta analysis (see Methods section GWAS).

Study	var_id	rs_id	beta	ste	pv	n_samples
Meta	1:201690989:C:T:b37	rs560804	-0.0408	0.0071	9.30E-09	41203
Charge	1:201690989:C:T:b37	rs560804	-0.0240	0.0149	1.07E-01	9417
UKBB	1:201690989:C:T:b37	rs560804	-0.0430	0.0076	1.50E-08	31786
Meta	1:21974834:C:T:b37	rs4654975	-0.0151	0.0074	4.06E-02	41203
Charge	1:21974834:C:T:b37	rs4654975	-0.0858	0.0155	2.82E-08	9417
UKBB	1:21974834:C:T:b37	rs4654975	0.0056	0.0080	4.90E-01	31786
Meta	13:22862729:C:T:b37	rs12863716	-0.0514	0.0085	1.21E-09	41203
Charge	13:22862729:C:T:b37	rs12863716	-0.0698	0.0176	7.40E-05	9417
UKBB	13:22862729:C:T:b37	rs12863716	-0.0432	0.0091	1.90E-06	31786
Meta	19:45382675:G:A:b37	rs41290120	-0.0995	0.0167	2.85E-09	41203
Charge	19:45382675:G:A:b37	rs41290120	-0.1331	0.0374	3.70E-04	9417
UKBB	19:45382675:G:A:b37	rs41290120	-0.0857	0.0177	1.40E-06	31786
Meta	7:19049388:G:A:b37	rs2107595	0.0899	0.0097	1.47E-20	41203
Charge	7:19049388:G:A:b37	rs2107595	0.1179	0.0196	1.79E-09	9417
UKBB	7:19049388:G:A:b37	rs2107595	0.0762	0.0104	2.70E-13	31786

Table S10: Genetic correlation between AAC and 773 complex traits calculated using LD Score Regression.

(see attached Excel sheets)

Table S11: Colocalization of AAC signals with expression in different tissues.

var index (b37 build)	tissue	gene	PP0	PP1	PP2	PP3	PP4	PP3 + PP4	log(PP4/PP3)
1:201690989:C:T	pancreas	SHISA4	4.30E-05	1.38E-02	8.16E-05	0.025	0.961	0.986	5.246
1:201690989:C:T	heart atrial appendage	NAV1	6.61E-07	2.13E-04	2.48E-04	0.079	0.921	1.000	3.548
1:201690989:C:T	heart left ventricle	NAV1	5.18E-04	1.67E-01	2.57E-04	0.082	0.750	0.832	3.193
1:201690989:C:T	adrenal gland	NAV1	6.92E-04	2.23E-01	2.56E-04	0.082	0.695	0.776	3.090
1:201690989:C:T	colon transverse	CACNA1S	6.93E-04	2.23E-01	3.11E-04	0.100	0.676	0.776	2.765
13:22862729:C:T	tibial artery	FGF9	1.20E-05	3.20E-02	6.16E-06	0.015	0.953	0.968	5.946
13:22862729:C:T	aorta	FGF9	1.02E-04	2.71E-01	3.58E-05	0.095	0.634	0.729	2.741
13:22862729:C:T	tibial artery	MICU2	1.92E-04	5.11E-01	3.19E-05	0.085	0.404	0.488	2.256
13:22862729:C:T	ovary	LINC00621	2.06E-04	5.50E-01	4.37E-05	0.116	0.334	0.450	1.523
13:22862729:C:T	artery aorta	SGCG	2.17E-04	5.77E-01	3.53E-05	0.094	0.329	0.422	1.808
19:45382675:G:A	lung	BCAM	2.67E-10	1.12E-07	8.92E-04	0.375	0.625	0.999	0.738
19:45382675:G:A	muscle skeletal	CKM	9.90E-04	4.16E-01	2.09E-04	0.088	0.495	0.583	2.498
19:45382675:G:A	adipose subcutaneous	AC092301.3	1.13E-03	4.76E-01	7.45E-05	0.031	0.492	0.522	3.994
19:45382675:G:A	heart left ventricle	PVRL2	3.19E-05	1.34E-02	1.50E-03	0.631	0.354	0.985	-0.832
19:45382675:G:A	artery tibial	AC069278.4	1.41E-03	5.94E-01	2.02E-04	0.084	0.320	0.404	1.920
7:19049388:G:A	artery aorta	TWIST1	1.03E-15	3.22E-02	3.92E-16	0.011	0.956	0.968	6.394
7:19049388:G:A	artery aorta	AC003986.6	2.69E-15	8.45E-02	1.18E-15	0.036	0.879	0.915	4.603
7:19049388:G:A	minor salivary gland	RPL21P75	1.55E-14	4.87E-01	1.92E-15	0.060	0.454	0.513	2.921
7:19049388:G:A	adipose subcutaneous	HDAC9	1.88E-14	5.91E-01	2.25E-15	0.071	0.339	0.409	2.265
7:19049388:G:A	testis	AC007091.1	1.74E-14	5.46E-01	3.81E-15	0.120	0.334	0.454	1.484

Table S12: Colocalization of AAC with complex traits in UK Biobank.

(See attached Excel file)

Table S13: Colocalization of AAC signal with cardiovascular traits from independent studies.

var index	var conditional	trait	PP0	PP1	PP2	PP3	PP4	PP3 + PP4	log(PP4/PP3)
1:201690989:C:T	NA	diastolic blood pressure	1.77E-05	6.23E-03	2.53E-04	0.088	0.905	0.994	3.356
1:201690989:C:T	NA	systolic blood pressure	2.75E-03	9.69E-01	6.78E-05	0.024	0.005	0.029	-2.316
1:201690989:C:T	NA	pulse pressure	1.55E-03	5.47E-01	7.29E-04	0.257	0.194	0.450	-0.406
1:201690989:C:T	NA	coronary artery disease	2.00E-03	7.06E-01	7.87E-04	0.277	0.014	0.291	-4.337
13:22862729:C:T	NA	diastolic blood pressure	3.63E-04	9.68E-01	1.03E-05	0.028	0.004	0.032	-2.714
13:22862729:C:T	NA	systolic blood pressure	3.54E-04	9.45E-01	1.76E-05	0.047	0.008	0.055	-2.512
13:22862729:C:T	NA	pulse pressure	3.28E-04	8.74E-01	1.05E-05	0.028	0.098	0.126	1.810
13:22862729:C:T	NA	coronary artery disease	3.61E-04	9.61E-01	6.88E-06	0.018	0.020	0.038	0.119
19:45382675:G:A	NA	diastolic blood pressure	2.16E-03	9.07E-01	1.21E-04	0.051	0.040	0.091	-0.349
19:45382675:G:A	NA	systolic blood pressure	2.31E-03	9.73E-01	4.26E-05	0.018	0.007	0.025	-1.325
19:45382675:G:A	NA	pulse pressure	2.07E-03	8.73E-01	1.81E-04	0.076	0.049	0.125	-0.626
19:45382675:G:A	NA	coronary artery disease	4.68E-10	1.97E-07	7.30E-06	0.002	0.998	1.000	8.913
7:19045397:A:G	7:19049388:G:A	diastolic blood pressure	4.35E-02	9.14E-01	1.51E-03	0.032	0.009	0.041	-1.828
7:19045397:A:G	7:19049388:G:A	systolic blood pressure	5.00E-03	1.05E-01	7.83E-04	0.016	0.874	0.889	5.810
7:19045397:A:G	7:19049388:G:A	pulse pressure	4.01E-11	8.43E-10	4.54E-04	0.009	0.991	1.000	6.858
7:19045397:A:G	7:19049388:G:A	coronary artery disease	7.27E-07	1.53E-05	1.18E-03	0.024	0.975	0.999	5.357
7:19049388:G:A	NA	diastolic blood pressure	3.01E-14	9.48E-01	1.05E-15	0.033	0.019	0.052	-0.756

7:19049388:G:A	NA	systolic blood pressure	2.04E-15	6.42E-02	3.80E-16	0.011	0.925	0.936	6.390
7:19049388:G:A	NA	pulse pressure	2.97E-25	9.35E-12	4.80E-17	0.001	0.999	1.000	10.942
7:19049388:G:A	NA	coronary artery disease	8.32E-21	2.61E-07	5.01E-17	0.001	0.999	1.000	10.764

Table S14: Disease codes and prognostic event counts

The PheWAS codes for which the CoxPH model was applied. We also show the number of people with the disease at baseline as well as the number of events during the follow up time. Participants who had events prior to baseline are not included in the CoxPH analysis.

Phecode	Phenotype	Prior Events	Number of Events
185	Cancer of prostate	547	260
366	Cataract	1150	563
401.1	Essential hypertension	5317	1834
411.3	Angina pectoris	1098	329
411.8	Other chronic ischemic heart disease, unspecified	1549	505
428.2	Heart failure NOS	191	185
596	Other disorders of bladder	674	159
600	Hyperplasia of prostate	1142	473
174.11	Malignant neoplasm of female breast	775	154
198.1	Secondary malignancy of lymph nodes	220	168
228	Hemangioma and lymphangioma, any site	156	44
220	Benign neoplasm of ovary	141	26
278.1	Obesity	687	622
531.2	Gastric ulcer	304	83
574.1	Cholelithiasis	1092	243
574.3	Cholecystitis without cholelithiasis	195	46
364	Corneal opacity and other disorders of cornea	73	92
366.2	Senile cataract	852	611
362	Other retinal disorders	310	205
367	Disorders of refraction and accommodation; blindness and low vision	239	268
519.8	Other diseases of respiratory system, NEC	343	156
535	Gastritis and duodenitis	2653	718
550.2	Diaphragmatic hernia	2347	641
455	Hemorrhoids	2230	41
495	Asthma	2118	499

564	Functional digestive disorders	802	449
280.1	Iron deficiency anemias, unspecified or not due to blood loss	645	255
250.1	Type 1 diabetes	106	38
250.2	Type 2 diabetes	990	443
361.1	Retinal detachment with retinal defect	293	72
530.11	GERD	2210	921
562.1	Diverticulosis	2518	1143
569	Other disorders of intestine	1298	561
727.1	Synovitis and tenosynovitis	422	98
728.7	Fasciitis	374	145
471	Nasal polyps	311	40
411.2	Myocardial infarction	558	167
602	Other disorders of prostate	147	25
8	Intestinal infection	544	316
300.13	Phobia	39	38
565	Anal and rectal conditions	356	42
573	Other disorders of liver	236	196
760	Back pain	998	379
454.1	Varicose veins of lower extremity	932	110
189.21	Malignant neoplasm of bladder	117	57
550.5	Ventral hernia	176	38
375	Disorders of lacrimal system	179	66
835	Internal derangement of knee	1473	162
415.11	Pulmonary embolism and infarction, acute	186	109
706.2	Sebaceous cyst	865	86
681	Superficial cellulitis and abscess	471	134
618.1	Prolapse of vaginal walls	860	129
622.1	Polyp of corpus uteri	1051	76
726	Peripheral enthesopathies and allied syndromes	798	124
218.1	Uterine leiomyoma	1085	63
214	Lipoma	683	96
216	Benign neoplasm of skin	859	113
427.2	Atrial fibrillation and flutter	877	495

472	Chronic pharyngitis and nasopharyngitis	109	36
389	Hearing loss	65	43
458.9	Hypotension NOS	270	211
496.21	Obstructive chronic bronchitis	294	210
496.3	Bronchiectasis	122	89
716.2	Unspecified monoarthritis	1081	430
785	Abdominal pain	1138	69
537	Other disorders of stomach and duodenum	662	302
540.11	Acute appendicitis	259	54
208	Benign neoplasm of colon	1180	556
340	Migraine	310	139
506	Empyema and pneumothorax	37	32
427.1	Paroxysmal tachycardia, unspecified	225	76
557	Intestinal malabsorption (non-celiac)	202	47
575	Other biliary tract disease	142	44
550.1	Inguinal hernia	1519	313
275	Disorders of mineral metabolism	130	119
619.1	Noninflammatory disorders of ovary, fallopian tube, and broad ligament	535	53
374	Other disorders of eyelids	524	126
564.1	Irritable Bowel Syndrome	547	219
342	Hemiplegia	56	52
433.2	Occlusion of cerebral arteries	152	124
433.8	Late effects of cerebrovascular disease	27	30
599.1	Urinary obstruction	182	93
601.1	Prostatitis	178	44
480	Pneumonia	400	296
433.1	Occlusion and stenosis of precerebral arteries	53	31
585.1	Acute renal failure	148	247
531.3	Duodenal ulcer	255	49
433.3	Cerebral ischemia	99	102
79	Viral infection	174	51
440	Atherosclerosis	33	26
443.8	Other specified peripheral vascular diseases	168	91

585.3	Chronic renal failure [CKD]	244	270
608	Other disorders of male genital organs	272	34
530.1	Esophagitis, GERD and related diseases	782	126
198	Secondary malignant neoplasm	30	170
172.3	Carcinoma in situ of skin	68	32
287	Purpura and other hemorrhagic conditions	93	61
451	Phlebitis and thrombophlebitis	232	89
274.1	Gout	232	181
362.7	Hereditary retinal dystrophies	51	29
365	Glaucoma	371	255
475	Chronic sinusitis	241	50
604.1	Redundant prepuce and phimosis/BXO	207	34
351	Other peripheral nerve disorders	894	162
938.2	Chronic dermatitis due to solar radiation	297	118
368	Visual disturbances	222	118
327	Sleep disorders	422	126
721	Spondylosis and allied disorders	469	278
261.2	Vitamin B-complex deficiencies	62	56
597.1	Urethral stricture (not specified as infectious)	266	48
507	Pleurisy; pleural effusion	204	206
367.9	Blindness and low vision	42	32
316	Substance addiction and disorders	253	80
345	Epilepsy, recurrent seizures, convulsions	179	56
702.2	Seborrheic keratosis	375	95
740.1	Osteoarthritis; localized	117	316
696.4	Psoriasis	189	98
594	Urinary calculus	407	86
172.11	Melanomas of skin	262	92
521.1	Dental caries	300	45
694.2	Other dyschromia	111	25
701	Other hypertrophic and atrophic conditions of skin	352	46
242	Thyrotoxicosis with or without goiter	133	45
476	Allergic rhinitis	180	179
577.1	Acute pancreatitis	93	40

333	Extrapyramidal disease and abnormal movement disorders	36	42
353	Nerve root and plexus disorders	338	110
568	Other disorders of peritoneum	224	141
285	Other anemias	670	309
529	Diseases and other conditions of the tongue	111	32
395.2	Nonrheumatic aortic valve disorders	95	122
174.1	Breast cancer [female]	185	35
202.2	Non-Hodgkins lymphoma	106	39
522	Diseases of pulp and periapical tissues	245	36
386	Vertiginous syndromes and other disorders of vestibular system	100	60
288.11	Neutropenia	144	68
592.1	Cystitis	223	46
153.3	Malignant neoplasm of rectum, rectosigmoid junction, and anus	37	43
251	Other disorders of pancreatic internal secretion	26	31
715	Other inflammatory spondylopathies	87	76
112	Candidiasis	123	63
395.1	Nonrheumatic mitral valve disorders	131	110
512	Other symptoms of respiratory system	152	171
480.2	Viral pneumonia	2	32
575.8	Other disorders of biliary tract	79	68
276.5	Hypovolemia	99	111
555.2	Ulcerative colitis	221	61
686	Other local infections of skin and subcutaneous tissue	91	37
496.1	Emphysema	50	43
733	Other disorders of bone and cartilage	123	36
555.1	Regional enteritis	93	27
379.2	Disorders of vitreous body	107	76
153.2	Colon cancer	31	82
261.4	Vitamin D deficiency	63	90
425.1	Primary/intrinsic cardiomyopathies	55	42
550.4	Umbilical hernia	286	70

150	Cancer of esophagus	7	26
736	Other acquired deformities of limbs	64	49
411.9	Other acute and subacute forms of ischemic heart disease	111	56
722.9	Other and unspecified disc disorder	88	37
420.2	Pericarditis	61	44
590	Pyelonephritis	80	31
447	Other disorders of arteries and arterioles	62	31
195.1	Malignant neoplasm, other	51	94
737.3	Kyphoscoliosis and scoliosis	21	27
465	Acute upper respiratory infections of multiple or unspecified sites	73	30
157	Pancreatic cancer	4	38
189.11	Malignant neoplasm of kidney, except pelvis	35	31
441	Vascular insufficiency of intestine	61	35
707.1	Decubitus ulcer	7	25
225.1	Benign neoplasm of brain, cranial nerves, meninges	8	33
165.1	Cancer of bronchus; lung	21	46
939	Atopic/contact dermatitis due to other or unspecified	40	28
352.1	Trigeminal nerve disorders [CN5]	12	30
276.5	Hypovolemia	18	87
345	Epilepsy, recurrent seizures, convulsions	80	115
736	Other acquired deformities of limbs	25	46
261.4	Vitamin D deficiency	3	59
512	Other symptoms of respiratory system	36	123
281.12	Other vitamin B12 deficiency anemia	10	39
585.2	Renal failure NOS	17	26
585.1	Acute renal failure	19	147
737.3	Kyphoscoliosis and scoliosis	7	37
585.3	Chronic renal failure [CKD]	10	229
362.7	Hereditary retinal dystrophies	8	35
496.1	Emphysema	6	44
332	Parkinson's disease	3	25
300.13	Phobia	8	30
433.8	Late effects of cerebrovascular disease	8	26
